# Supplementary material for: Impact of chronic kidney disease on case ascertainment for hospitalised acute myocardial infarction: an English cohort study
Source: BMJ Open. 2022 Mar 28;12(3):e057909. doi: 10.1136/bmjopen-2021-057909 (PMC8961119; doi:10.1136/bmjopen-2021-057909)
Supplement: Supplementary data [file bmjopen-2021-057909supp001.pdf]

Supplementary materials

Supplementary tables

Supplementary table 1: ICD-10 codes for AMI identified in HES

Supplementary table 2: CALIBER definition of AMI subtypes (STEMI, NSTEMI) using MINAP data.

Supplementary table 3: Variables used to define death in-hospital and post-AMI discharge.

Supplementary table 4: Details on data sources for covariates.

Supplementary table 5: AMI database capture by CKD status (binary) stratified by AMI subtype (STEMI or NSTEMI)

Supplementary table 6: Serum creatinine (SCr) measured in the first 24 hours of AMI hospitalization recorded in MINAP, stratified by CKD stage defined in primary care (NCKDA)

Supplementary table 7: AMI hospitalisations occurring before study start – description of people in the NCKDA who experienced AMI hospitalisation recorded in MINAP or HES before the study start. n (column %) unless specified otherwise.

Supplementary table 8: AMI hospitalisations occurring before study start - multinomial logistic regression comparing the relative risk ratios (RRR) of AMI recording across HES and MINAP according to CKD stage. The comparator outcome is people with AMI recorded in both HES and MINAP databases.

Supplementary table 9: AMI hospitalisations occurring before study start - agreement between primary care-derived CKD stage (NCKDA) and secondary care-derived CKD stage (MINAP)

Supplementary table 10: Multinomial logistic regression comparing the relative risk ratios (RRR) of AMI recording across HES and MINAP according to CKD stage, after excluding those with a history of dialysis. The comparator outcome is people with AMI recorded in both HES and MINAP databases.

Supplementary table 11: Death during and after first AMI hospitalisation in study population at risk of or with CKD, excluding those with a history of dialysis.

Supplementary table 12: Sensitivity analysis describing the ICD-10 diagnoses (first episode, first diagnostic position) of non-AMI HES cases that are within ±30 days of MINAP only AMI cases from the main analyses

Supplementary figures

BMJ Publishing Group Limited (BMJ) disclaims all liability and responsibility arising from any reliance placed on this supplemental material which has been supplied by the author(s)

Supplementary figure 1: Flow diagram illustrating the NCKDA extracts, and resulting contribution of AMI events to this study

Supplementary figure 2: AMI case ascertainment in MINAP and HES datasets over time.

Supplementary figure 3: Bland-Altman plot comparing the mean eGFR (NCKDA eGFR and MINAP eGFR, x-axis) and the difference between the NCKDA eGFR and the MINAP eGFR (y-axis).

Supplementary figure 4: AMI hospitalisations occurring before study start - comparing AMI case ascertainment overall and by CKD status for AMI hospitalisations in the main analysis and AMI hospitalisations which occurred prior to the last NCKDA extract (sensitivity analysis).

Supplementary figure 5: Sensitivity analyses compared to main analysis, describing AMI recording in MINAP and HES in the overall study population at risk of or with CKD. Areas of Venn diagrams are proportional to the number of cases.

Supplementary table 1: ICD-10 codes for AMI identified in HES

AMI subtype	ICD-10 codes
Overall	I21, I22, I23
STEMI	I21.0, I21.1, I21.2, I21.3, I22.0, I22.1, I22.8
NSTEMI	I21.4, I21.9, I22.9
Missing	I23

AMI: acute myocardial infarction; HES: Hospital Episode Statistics; ICD-10: International Classification of Diseases 10th Edition; STEMI: ST elevated myocardial infarction; NSTEMI: Non-ST elevated myocardial infarction

BMJ Publishing Group Limited (BMJ) disclaims all liability and responsibility arising from any reliance placed on this supplemental material which has been supplied by the author(s)

Supplementary table 2: CALIBER definition of AMI subtypes (STEMI, NSTEMI) using MINAP data.

	**MINAP variable**		
**AMI subtype**	**Discharge diagnosis**	**Markers elevated?**	**ECG result**
Other	Threatened MI, Chest pain uncertain cause, MI unconfirmed, other diagnosis	-	-
STEMI	STEMI	Raised or missing	ST elevation, LBBB, or ST elevation
	NSTEMI/Troponin positive ACS	Raised or missing	ST elevation
	ACS troponin negative	Raised	ST elevation
	ACS troponin unspecified	Raised	ST elevation
NSTEMI	NSTEMI/Troponin positive ACS	Raised or missing	ST depression, T wave changes only, other abnormality, Normal ECG, or LBBB
	ACS troponin negative	Raised	LBBB, ST depression, T wave changes only, Other abnormality, normal ECG, or missing
	ACS troponin unspecified	Raised	LBBB, ST depression, T wave changes only, Other abnormality, normal ECG, or missing
Unstable angina	*Any remaining hospitalisations not assigned as STEMI, NSTEMI, or other diagnosis		

ACS: acute coronary syndrome, AMI: acute myocardial infarction, ECG: electrocardiogram, LBBB: left bundle branch block, MI: myocardial infarction, MINAP: Myocardial Ischaemia National Audit Project, NSTEMI: Non ST-elevation myocardial infarction, STEMI: ST-elevation myocardial infarction

Supplementary table 3: Variables used to define death in-hospital and post-AMI discharge.

**Data source**	**Variables to define in-hospital death**	**Variables to define death post-AMI discharge (in those who survive first AMI hospitalization)**
ONS	Date of death (falling on or in between first AMI admission and discharge dates)	Date of death (falling after first AMI discharge date)
MINAP	Reason for no angiography, no intervention, admission ward, discharge destination	-
HES	Discharge location, discharge method	-

AMI: acute myocardial infarction, HES: Hospital Episode Statistics, MINAP: Myocardial Ischaemia National Audit Project, ONS: Office of National Statistics

Supplementary table 4: Details on data sources for covariates.

Category	Covariate	Data source
Sociodemographic and lifestyle variables	Age at AMI admission	NCKDA
	Sex	NCKDA
	IMD quintiles	NCKDA
	Smoking status	NCKDA, MINAP
Comorbidities	Angina	MINAP and HES
	Cerebrovascular disease	MINAP and HES
	COPD	MINAP and HES
	Diabetes mellitus	MINAP and HES
	Heart failure	MINAP and HES
	Hypertension	NCKDA, MINAP, and HES
	Previous myocardial infarction	MINAP, and HES
	Peripheral vascular disease	NCKDA, MINAP, and HES
	Dialysis	NCKDA
	Kidney transplant	NCKDA

AMI: Acute Myocardial Infarction, COPD: Chronic Obstructive Pulmonary Disease, HES: Hospital Episode Statistics, IMD: Index of Multiple Deprivation, NCKDA: National Chronic Kidney Disease Audit, MINAP: Myocardial Ischaemia National Audit Project

Supplementary table 5: AMI database capture by CKD status (binary) stratified by AMI subtype (STEMI or NSTEMI)

**AMI subtype**	**Database** (outcome, compared to people with AMI recorded in MINAP & HES)	**CKD stage** (exposure)	**Number of AMI admissions**	**Unadjusted RRR (95% CI)**	**Adjusted RRR[1] (95% CI)**
**STEMI**	**MINAP only** (N=115)	At risk of CKD & Stages 1-2	31	1	1
		Stage 3a-5	74	1.08 (0.68-1.72)	0.88 (0.52-1.49)
	**HES only** (N=1,482)	At risk of CKD & Stages 1-2	1,306	1	1
		Stage 3a-5	194	0.07 (0.05-0.09)	0.07 (0.05-0.09)
**NSTEMI**	**MINAP only** (N=666)	At risk of CKD & Stages 1-2	167	1	1
		Stage 3a-5	474	1.16 (0.93-1.45)	1.11 (0.88-1.41)
	**HES only** (N=2,834)	At risk of CKD & Stages 1-2	2,164	1	1
		Stage 3a-5	717	0.14 (0.12-0.16)	0.15 (0.12-0.17)

[1] adjusted for sex, age group at AMI admission, ethnicity, previous AMI, and clustering by patient

AMI: Acute Myocardial Infarction, CKD: chronic kidney disease, HES: Hospital Episode Statistics, MINAP: Myocardial Ischaemia National Audit Project, NSTEMI: non-ST elevation myocardial infarction, RRR: relative risk ratio, STEMI: ST-elevation myocardial infarction

Supplementary table 6: Serum creatinine (SCr) measured in the first 24 hours of AMI hospitalization recorded in MINAP, stratified by CKD stage defined in primary care (NCKDA)

**CKD status (NCKDA)**	**SCr recorded in MINAP at AMI admission, n (%)**	**MINAP median SCr, umol (IQR)**	**MINAP Median SCr value*, mg/dL (IQR)**	**MINAP Median eGFR, mL/min/1.73m^2 (IQR)**
All stages	2,240 (97)	115 (91-152)	1.30 (1.03-1.72)	47.6 (33.5-61.6)
At risk of CKD & Stages 1-2	630 (97)	88 (74-104)	1.00 (0.84-1.18)	67.0 (56.0-80.2)
3a	770 (97)	109 (94-128)	1.23 (1.06-1.45)	50.8 (41.9-59.2)
3b	587 (98)	140 (117-174)	1.58 (1.32-1.97)	36.7 (29.4-43.4)
4-5	253 (95)	255 (190-407)	2.88 (2.15-4.60)	19.8 (12.0-25.5)

*Multiplied by 0.95 for SCr taken before 2013

AMI: acute myocardial infarction, CKD: chronic kidney disease, eGFR: estimated glomerular filtration rate, NCKDA: National Chronic Kidney Disease Audit, MINAP: Myocardial Ischaemia National Audit Project, SCr: serum creatinine

Supplementary table 7: AMI hospitalisations occurring before study start – description of people in the NCKDA who experienced AMI hospitalisation recorded in MINAP or HES before the study start. n (column %) unless specified otherwise.

**CKD status** (main exposure)	**No CKD**	**CKD**			**Total**
**CKD stage** (secondary exposure)	**At-risk of CKD & 1-2**	**3a**	**3b**	**4-5**	
**Unique individuals**	27,909	4,884	2,083	726	34,860
**Total number of AMI events, N**	32,041	5,641	2,460	926	41,068
**Age at AMI event, years, mean (SD)**	64 (13)	75 (10)	78 (10)	75 (12)	66 (13)
**Age category at AMI event, years**					
18-50	4,506 (14)	83 (1)	22 (1)	34 (4)	4,645 (11)
50-64	11,964 (37)	829 (15)	207 (8)	130 (14)	13,130 (32)
65-79	11,517 (36)	2,777 (49)	986 (40)	405 (44)	15,685 (38)
80+	3,922 (12)	1,952 (35)	1,245 (51)	357 (39)	7,476 (18)
Missing	132 (0)	0 (0)	0 (0)	0 (0)	132 (0)
**Female**	9,288 (29)	2,482 (44)	1,209 (49)	394 (43)	13,373 (33)
**Ethnicity**					
White	29,016 (91)	5,228 (93)	2,257 (92)	846 (91)	37,347 (91)
Other	2,612 (8)	295 (5)	139 (6)	71 (8)	3,117 (8)
Missing	413 (1)	118 (2)	64 (3)	9 (1)	604 (1)
**IMD quintile**					
1 (least deprived)	5,808 (18)	1,121 (20)	456 (19)	184 (20)	7,569 (18)
2	6,716 (21)	1,284 (23)	523 (21)	182 (20)	8,705 (21)
3	6,922 (22)	1,297 (23)	542 (22)	180 (19)	8,941 (22)
4	6,820 (21)	1,134 (20)	525 (21)	216 (23)	8,695 (21)
5 (most deprived)	5,531 (17)	787 (14)	406 (17)	161 (17)	6,885 (17)
Missing	244 (1)	18 (0)	8 (0)	<5	273 (1)
**Dialysis in primary care**					
Peritoneal dialysis	0 (0)	0 (0)	0 (0)	12 (1)	12 (0)
Haemodialysis	0 (0)	0 (0)	0 (0)	24 (3)	24 (0)
Renal dialysis, unspecified	0 (0)	0 (0)	0 (0)	11 (1)	11 (0)
**Kidney transplant**	<5	<5	8 (0)	11 (1)	26 (0)
**Comorbidities**					
Angina	5,001 (16)	1,630 (29)	882 (36)	335 (36)	7,848 (19)
Cerebrovascular disease	1,405 (4)	497 (9)	309 (13)	135 (15)	2,346 (6)

CKD status (main exposure)	No CKD	CKD			Total
**CKD stage** (secondary exposure)	**At-risk of CKD & 1-2**	**3a**	**3b**	**4-5**	
**Unique individuals**	27,909	4,884	2,083	726	34,860
**Total number of AMI events, N**	32,041	5,641	2,460	926	41,068
COPD	2,059 (6)	680 (12)	368 (15)	147 (16)	3,254 (8)
Diabetes	5,823 (18)	1,500 (27)	873 (35)	463 (50)	8,659 (21)
Heart failure	1,228 (4)	456 (8)	353 (14)	199 (21)	2,236 (5)
Hypertension	10,556 (33)	3,287 (58)	1,721 (70)	673 (73)	16,237 (40)
Myocardial infarction	5,563 (17)	1,510 (27)	832 (34)	380 (41)	8,285 (20)
Peripheral vascular disease	799 (2)	258 (5)	181 (7)	106 (11)	1,344 (3)
**Smoking status**					
non-smoker	12,945 (40)	2,049 (36)	835 (34)	301 (33)	16,130 (39)
ever-smoker	16,626 (52)	2,477 (44)	983 (40)	382 (41)	20,468 (50)
Missing	2,470 (8)	1,115 (20)	642 (26)	243 (26)	4,470 (11)

AMI: Acute Myocardial Infarction, CKD: chronic kidney disease, COPD: Chronic Obstructive Pulmonary Disease, HES: Hospital Episode Statistics, IMD: Index of Multiple Deprivation, NCKDA: National Chronic Kidney Disease Audit, MINAP: Myocardial Ischaemia National Audit Project

Supplementary table 8: AMI hospitalisations occurring before study start - multinomial logistic regression comparing the relative risk ratios (RRR) of AMI recording across HES and MINAP according to CKD stage. The comparator outcome is people with AMI recorded in both HES and MINAP databases.

**AMI recording** (outcome, compared to people with AMI recorded in MINAP & HES)	**CKD stage** (exposure)	**Number of AMI admissions, n=**	**Unadjusted[1] RRR (95% CI)**	**Partially adjusted[2] RRR (95% CI)**	**Adjusted[3] RRR (95% CI)**
**MINAP only** (N=3,957)	At-risk/Stages 1-2	2,147	1	1	1
	Stage 3a	999	0.96 (0.87-1.05)	0.90 (0.82-0.99)	0.88 (0.80-0.97)
	Stage 3b	578	1.22 (1.09-1.36)	1.12 (0.99-1.26)	1.05 (0.93-1.19)
	Stages 4-5	233	1.35 (1.14-1.60)	1.27 (1.07-1.51)	1.13 (0.94-1.35)
**HES only** (N=26,697)	At-risk/Stages 1-2	23,904	1	1	1
	Stage 3a	1,911	0.16 (0.15-0.18)	0.23 (0.22-0.25)	0.24 (0.22-0.25)
	Stage 3b	630	0.12 (0.11-0.13)	0.20 (0.17-0.22)	0.21 (0.18-0.23)
	Stages 4-5	252	0.13 (0.11-0.15)	0.18 (0.15-0.22)	0.20 (0.17-0.24)

[1]Complete cases for adjusted model
[2]Adjusted for sex, age at AMI admission, ethnicity (white, other), IMD quintile, clustering by participant
[3]Additionally adjusted for comorbidities previous AMI, heart failure, COPD, diabetes mellitus

AMI: Acute Myocardial Infarction, CKD: chronic kidney disease, COPD: Chronic Obstructive Pulmonary Disease, HES: Hospital Episode Statistics, IMD: Index of Multiple Deprivation, MINAP: Myocardial Ischaemia National Audit Project, RRR: relative risk ratio

Supplementary table 9: AMI hospitalisations occurring before study start - agreement between primary care-derived CKD stage (NCKDA) and secondary care-derived CKD stage (MINAP)

	% agreement	Kappa statistic (SE)
**Overall[1]**	66.4%	0.51 (0.007)
**CKD stages 3a, 3b, 4-5[2]**	57.8%	0.37 (0.008)
**CKD stages 1-2, 3a-5[3]**	80.3%	0.59 (0.010)
**Overall[1], by time from NCKDA SCr test (primary care) to MINAP SCr test (at AMI secondary care admission)**		
0-5 months	66.7%	0.52 (0.009)
6-11 months	65.6%	0.49 (0.013)
12-23 months	63.3%	0.42 (0.023)
24-36 months	65.0%	0.45 (0.034)

[1]Overall agreement when grouping as (1) Stages 1-2 (eGFR 60-120mL/min/1.73m^2), (2) Stage 3a (eGFR 45-59mL/min/1.73m^2), (3) Stage 3b (eGFR 30-44 mL/min/1.73m^2), (4) Stages 4-5 (eGFR 0-30 mL/min/1.73m^2)
[2]Agreement when restricting to people with CKD stages 3a-5, grouped as (1) Stage 3a (eGFR 45-59mL/min/1.73m^2), (2) Stage 3b (eGFR 30-44 mL/min/1.73m^2), (3) Stages 4-5 (eGFR 0-30 mL/min/1.73m^2)
[3]Agreement when grouping as (1) Stages 1-2 (eGFR 60-120mL/min/1.73m^2) and (2) Stages 3a-5 (eGFR 0-59mL/min/1.73m^2)

AMI: Acute Myocardial Infarction, CKD: chronic kidney disease, NCKDA: National Chronic Kidney Disease Audit, MINAP: Myocardial Ischaemia National Audit Project, SCr: serum creatinine

Supplementary table 10: Multinomial logistic regression comparing the relative risk ratios (RRR) of AMI recording across HES and MINAP according to CKD stage, after excluding those with a history of dialysis. The comparator outcome is people with AMI recorded in both HES and MINAP databases.

**AMI recording** (outcome, compared to people with AMI recorded in MINAP & HES)	**CKD stage** (exposure)	**Number of AMI admissions, n=**	**Unadjusted[1] RRR (95% CI)**	**Partially adjusted[2] RRR (95% CI)**	**Adjusted[3] RRR (95% CI)**
**MINAP only** (N=731)	At-risk/Stages 1-2	196	1	1	1
	Stage 3a	245	1.07 (0.85-1.34)	0.98 (0.77-1.25)	0.98 (0.77-1.25)
	Stage 3b	197	1.17 (0.92-1.49)	1.04 (0.80-1.36)	1.03 (0.79-1.35)
	Stages 4-5	93	1.54 (1.14-1.60)	1.41 (1.01-1.97)	1.36 (0.97-1.92)
**HES only** (N=4,353)	At-risk/Stages 1-2	3,456	1	1	1
	Stage 3a	557	0.14 (0.12-0.16)	0.14 (0.12-0.17)	0.14 (0.12-0.16)
	Stage 3b	224	0.08 (0.06-0.09)	0.08 (0.07-0.10)	0.08 (0.06-0.10)
	Stages 4-5	116	0.11 (0.08-0.14)	0.12 (0.09-0.15)	0.12 (0.09-0.16)

[1]Complete cases for adjusted model
[2]Adjusted for sex, age at AMI admission, ethnicity (white, other), IMD quintile, clustering by participant
[3]Additionally adjusted for comorbidities previous AMI, heart failure, COPD, diabetes mellitus

AMI: Acute Myocardial Infarction, CKD: chronic kidney disease, COPD: Chronic Obstructive Pulmonary Disease, HES: Hospital Episode Statistics, IMD: Index of Multiple Deprivation, MINAP: Myocardial Ischaemia National Audit Project, RRR: relative risk ratio

Supplementary table 11: Death during and after first AMI hospitalisation in study population at risk of or with CKD, excluding those with a history of dialysis.

**Death during first AMI hospitalisation (N=5,919)[1]**	**Number who died, n (%)**	-	**Unadjusted OR (95% CI)**	**Adjusted[2] OR (95% CI)**
MINAP & HES	208 (15)	-	1	1
MINAP only	148 (23)	-	1.65 (1.31-2.09)	1.59 (1.25-2.03)
HES only	575 (15)	-	0.97 (0.82-1.15)	1.61 (1.32-1.96)
**Death during complete follow-up in those who survive first AMI hospitalisation (N=5,009)[1]**	**Number who died during follow-up, n**	**Rate per 100 person-years (95% CI)**	**Unadjusted HR (95% CI)**	**Adjusted[2] HR (95% CI)**
MINAP & HES	445	17.5 (16.0-19.2)	1	1
MINAP only	234	23.4 (20.6-26.6)	1.30 (1.11-1.52)	1.14 (0.97-1.34)
HES only	841	10.3 (9.58-11.0)	0.61 (0.54-0.68)	1.09 (0.96-1.24)

[1]Complete cases for adjusted model

[2]Adjusted for sex, age at AMI admission, ethnicity (white, other), IMD quintile, previous AMI, heart failure, COPD, diabetes mellitus

AMI: Acute Myocardial Infarction, CKD: chronic kidney disease, COPD: Chronic Obstructive Pulmonary Disease, HES: Hospital Episode Statistics, HR: hazard ratio, IMD: Index of Multiple Deprivation, NCKDA: National Chronic Kidney Disease Audit, MINAP: Myocardial Ischaemia National Audit Project, OR: odds ratio

Supplementary table 12: Sensitivity analysis describing the ICD-10 diagnoses (first episode, first diagnostic position) of non-AMI HES cases that are within ±30 days of MINAP only AMI cases from the main analyses

**Main analysis**		
**MINAP only AMI hospitalisations matched with non-AMI HES hospitalisations**		677/766 (88%)
**Short ICD-10 code (1st episode, 1st diagnostic position)**		**n (% of matched)**
I24	Other acute ischaemic heart diseases	114 (17)
I25	Chronic ischaemic heart disease	84 (12)
R07	Pain in throat and chest	63 (9)
J18	Pneumonia, organism unspecified	56 (8)
I20	Angina pectoris	51 (8)
I50	Heart failure	39 (6)
S72	Fracture of femur	18 (3)
N39	Other disorders of urinary system	14 (2)
A41	Other sepsis	11 (2)
R06	Abnormalities of breathing	11 (2)
**Sensitivity analysis – AMI hospitalisations occurring before study start**		
**MINAP only AMI hospitalisations matched with non-AMI HES hospitalisations**		3,908/4,470 (87%)
**Short ICD-10 code (1st episode, 1st diagnostic position)**		**n (% of matched)**
I25	Chronic ischaemic heart disease	996 (25)
I20	Angina pectoris	760 (19)
R07	Pain in throat and chest	588 (15)
I24	Other acute ischaemic heart diseases	271 (7)
I50	Heart failure	129 (3)

J18	Pneumonia, organism unspecified	86 (2)
I48	Atrial fibrillation and flutter	73 (2)
R06	Abnormalities of breathing	49 (1)
I46	Cardiac arrest	42 (1)
S72	Fracture of femur	40 (1)

AMI: Acute Myocardial Infarction, HES: Hospital Episode Statistics, ICD-10: International Classification of Diseases – 10th Edition, MINAP: Myocardial Ischaemia National Audit Project

Supplementary figure 1: Flow diagram illustrating the NCKDA extracts, and resulting contribution of AMI events to this study

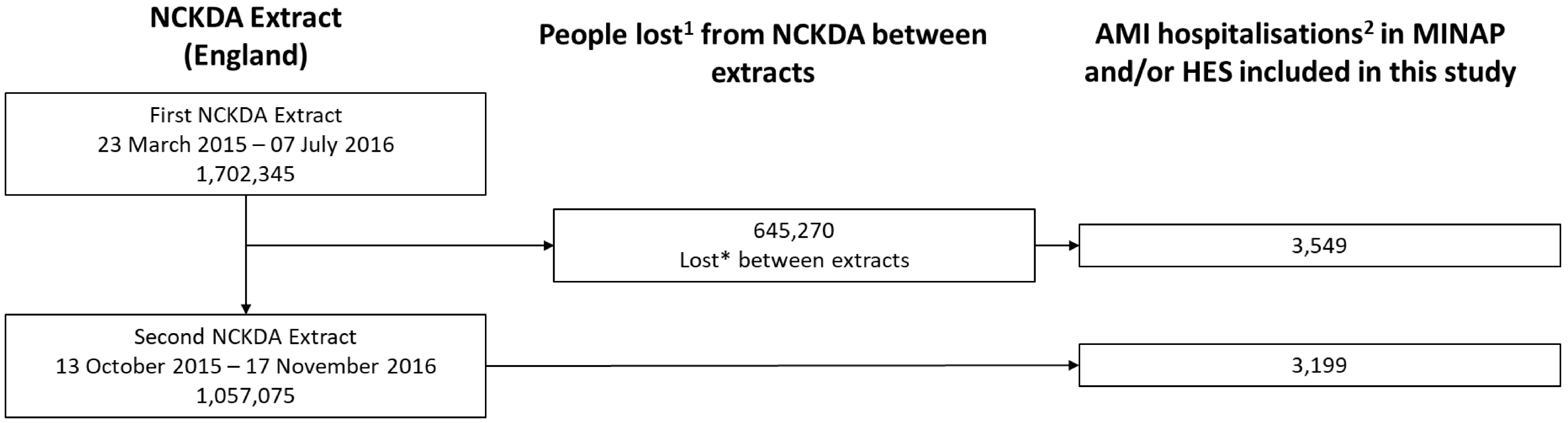

[1]Lost due to death, GP opted out, or person moved GPs
[2]AMI hospitalisations occurring after final NCKDA extract in which each person appears

AMI: acute myocardial infarction, GP: General Practice, NCKDA: National Chronic Kidney Disease Audit, MINAP: Myocardial Ischaemia National Audit Project

Supplementary figure 2: AMI case ascertainment in MINAP and HES datasets over time.

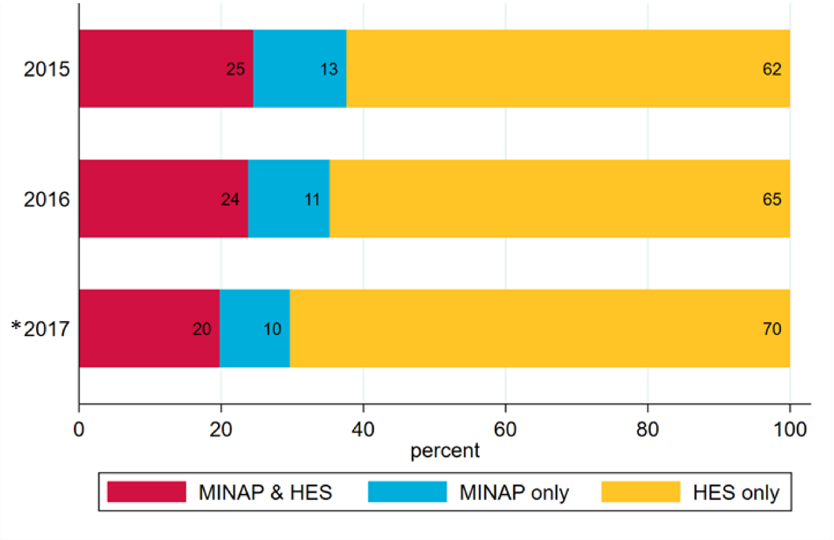

*2017 includes AMI hospitalisations up to 31 March only

AMI: acute myocardial infarction, HES: Hospital Episode Statistics, MINAP: Myocardial Ischaemia National Audit Project

Supplementary figure 3: Bland-Altman plot comparing the mean eGFR (NCKDA eGFR and MINAP eGFR, x-axis) and the difference between the NCKDA eGFR and the MINAP eGFR (y-axis).

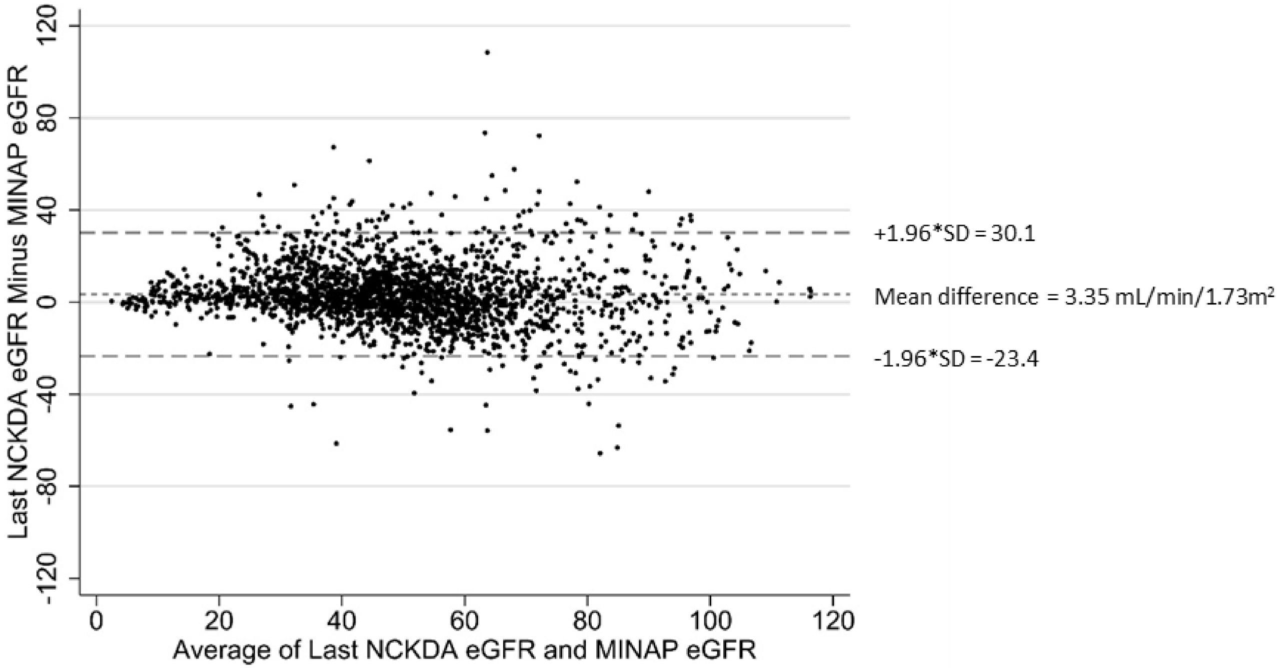

eGFR: estimated glomerular filtration rate, NCKDA: National Chronic Kidney Disease Audit, MINAP: Myocardial Ischaemia National Audit Project

Supplementary figure 4: AMI hospitalisations occurring before study start - comparing AMI case ascertainment overall and by CKD status for AMI hospitalisations in the main analysis and AMI hospitalisations which occurred prior to the last NCKDA extract (sensitivity analysis).

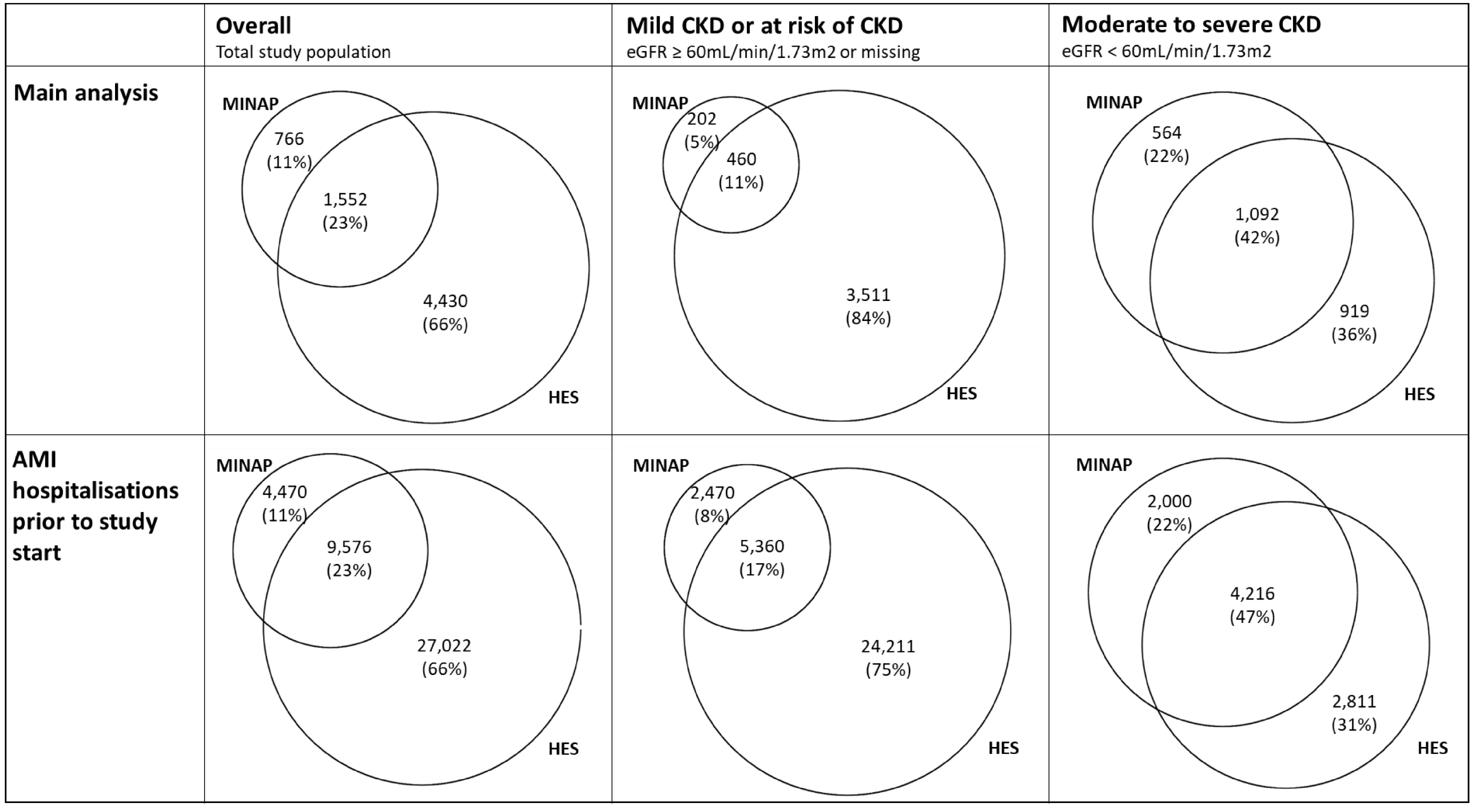

AMI: Acute Myocardial Infarction, CKD: chronic kidney disease, eGFR: estimated glomerular filtration rate, NCKDA: National Chronic Kidney Disease Audit, MINAP: Myocardial Ischaemia National Audit Project

Supplementary figure 5: Sensitivity analyses compared to main analysis, describing AMI recording in MINAP and HES in the overall study population at risk of or with CKD. Areas of Venn diagrams are proportional to the number of cases.

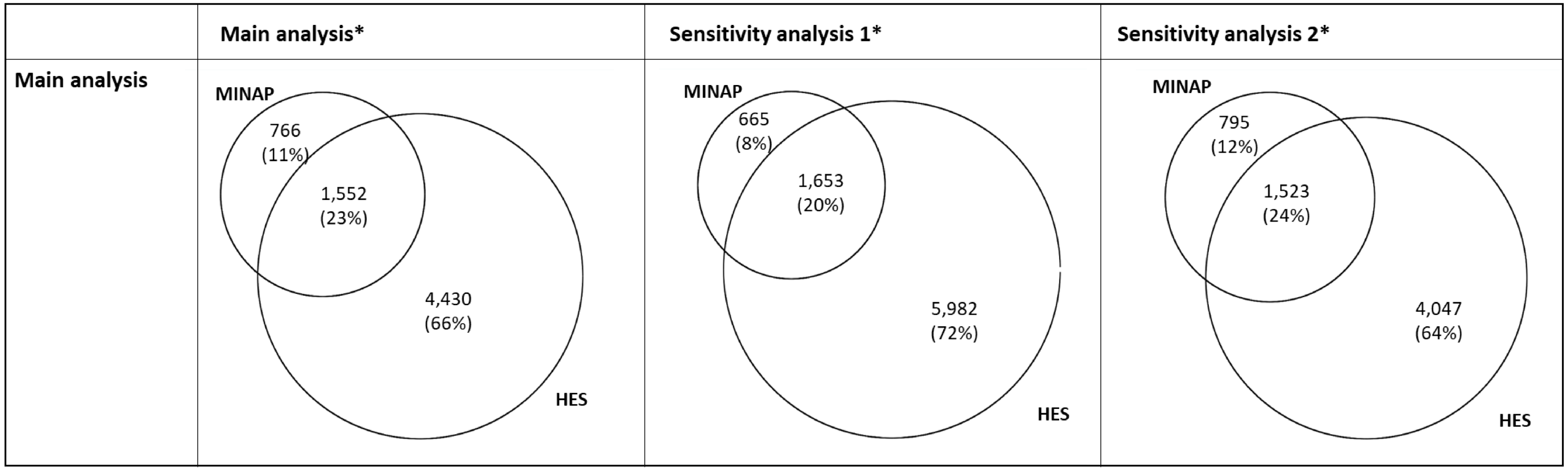

***Main analysis:** HES AMI cases defined as AMI recorded in 1st diagnostic position, 1st episode of hospitalisation
***Sensitivity analysis 1:** HES AMI cases defined as AMI recorded in 1st or 2nd diagnostic position, 1st episode of hospitalisation
***Sensitivity analysis 2:** HES AMI cases in 1st diagnostic position, 1st episode of hospitalisation (as per main analysis) within 30 days of each other counted as a single AMI case, then matched to MINAP AMI cases

AMI: Acute Myocardial Infarction, CKD: chronic kidney disease, HES: Hospital Episode Statistics, MINAP: Myocardial Ischaemia National Audit Project
