# Supplementary material for: Impact of chronic kidney disease on case ascertainment for hospitalised acute myocardial infarction: an English cohort study
Source: BMJ Open. 2022 Mar 28;12(3):e057909. doi: 10.1136/bmjopen-2021-057909 (PMC8961119; doi:10.1136/bmjopen-2021-057909)
Supplement: Author's manuscript [file bmjopen-2021-057909.draft_revisions.pdf]

BMJ Open is committed to open peer review. As part of this commitment we make the peer review history of every article we publish publicly available.

When an article is published we post the peer reviewers' comments and the authors' responses online. We also post the versions of the paper that were used during peer review. These are the versions that the peer review comments apply to.

The versions of the paper that follow are the versions that were submitted during the peer review process. They are not the versions of record or the final published versions. They should not be cited or distributed as the published version of this manuscript.

BMJ Open is an open access journal and the full, final, typeset and author-corrected version of record of the manuscript is available on our site with no access controls, subscription charges or pay-per-view fees (http://bmjopen.bmj.com).

If you have any questions on BMJ Open's open peer review process please email info.bmjopen@bmj.com

BMJ Open

The impact of chronic kidney disease on case ascertainment for hospitalised acute myocardial infarction: An English cohort study

Journal:	*BMJ Open*
Manuscript ID	bmjopen-2021-057909
Article Type:	Original research
Date Submitted by the Author:	04-Oct-2021

Complete List of Authors:	Bidulka, Patrick; London School of Hygiene and Tropical Medicine Faculty of Epidemiology and Population Health, Non-Communicable Disease Epidemiology<br>Scott, Jemima; University of Bristol, Population Health Sciences; North Bristol NHS Trust, Richard Bright Renal Service<br>Taylor, Dominic ; University of Bristol, Population Health Sciences; North Bristol NHS Trust<br>Udayaraj, Udaya; Churchill Hospital, Oxford Kidney Unit; University of Oxford, Nuffield Department of Medicine<br>Caskey, Fergus; University of Bristol, Population Health Sciences; North Bristol NHS Trust, Richard Bright Renal Service<br>Teece, Lucy; University of Leicester, Biostatistics Research Group<br>Sweeting, Michael; University of Leicester, Biostatistics Research Group<br>Deanfield, John; Barts Health NHS Trust, National Institute for Cardiovascular Outcomes Research (NICOR); University College London, Institute of Cardiovascular Sciences<br>de Belder, Mark; Barts Health NHS Trust, National Institute for Cardiovascular Outcomes Research (NICOR)<br>Denaxas, S; University College London, Institute of Health Informatics, Faculty of Population Health Sciences; Health Data Research UK<br>Weston, Clive; Glangwili General Hospital<br>Adlam, David; University of Leicester, Department of Sciences and NIHR Biomedical Research Centre<br>Nitsch, Dorothea; London School of Hygiene & Tropical Medicine, Department of Non-Communicable Disease Epidemiology

Keywords:	CARDIOLOGY, Audit < CARDIOLOGY, Myocardial infarction < CARDIOLOGY, NEPHROLOGY, STATISTICS & RESEARCH METHODS

SCHOLARONE™
Manuscripts

I, the Submitting Author has the right to grant and does grant on behalf of all authors of the Work (as defined in the below author licence), an exclusive licence and/or a non-exclusive licence for contributions from authors who are: i) UK Crown employees; ii) where BMJ has agreed a CC-BY licence shall apply, and/or iii) in accordance with the terms applicable for US Federal Government officers or employees acting as part of their official duties; on a worldwide, perpetual, irrevocable, royalty-free basis to BMJ Publishing Group Ltd ("BMJ") its licensees and where the relevant Journal is co-owned by BMJ to the co-owners of the Journal, to publish the Work in this journal and any other BMJ products and to exploit all rights, as set out in our licence.

The Submitting Author accepts and understands that any supply made under these terms is made by BMJ to the Submitting Author unless you are acting as an employee on behalf of your employer or a postgraduate student of an affiliated institution which is paying any applicable article publishing charge ("APC") for Open Access articles. Where the Submitting Author wishes to make the Work available on an Open Access basis (and intends to pay the relevant APC), the terms of reuse of such Open Access shall be governed by a Creative Commons licence – details of these licences and which Creative Commons licence will apply to this Work are set out in our licence referred to above.

Other than as permitted in any relevant BMJ Author's Self Archiving Policies, I confirm this Work has not been accepted for publication elsewhere, is not being considered for publication elsewhere and does not duplicate material already published. I confirm all authors consent to publication of this Work and authorise the granting of this licence.

For peer review only

Title:

The impact of chronic kidney disease on case ascertainment for hospitalised acute myocardial infarction: An English cohort study

Authors:

Patrick Bidulka* (1), Jemima Scott* (2,3), Dominic M Taylor (2,3), Udaya Udayaraj (4), Fergus Caskey (2,3), Lucy Teece (5), Michael Sweeting (5), John Deanfield (6,7), Mark de Belder (6), Spiros Denaxas (8,9), Clive Weston (10), David Adlam* (11), Dorothea Nitsch* (1)

*Equal contribution/joint-authorship

Order	Author	Affiliation	Job title at time of writing	Email
1	Bidulka P*	1	Research Fellow	patrick.bidulka1@lshtm.ac.uk
2	Scott J*	2,3	NIHR Doctoral Research Fellow	Js15922@bristol.ac.uk
3	Taylor DM	2,3	Consultant Nephrologist (NBT), Honorary Lecturer (UoB)	dominic.taylor@nbt.nhs.uk
4	Udayarai U	4,5	Consultant Nephrologist Honorary Senior Lecturer	Udaya.Udayaraj@ouh.nhs.uk
5	Caskey FJ	2,3	Consultant senior lecturer	fergus.caskey@bristol.ac.uk
6	Teece L	6	Research Fellow in Medical Statistics	lucy.teece@leicester.ac.uk
7	Sweeting MJ	6	Associate Professor in Biostatistics	michael.sweeting@leicester.ac.uk
8	Deanfield J	7,8	Professor of Cardiology	j.deanfield@ucl.ac.uk
9	de Belder M	7	Professor of Cardiology	mark.debelder@nhs.net
10	Denaxas S	9,10	Professor of Biomedical Informatics	s.denaxas@ucl.ac.uk
11	Weston C	11	Consultant Cardiologist	cliveweston@doctors.org.uk
12	Adlam D	12	Associate Professor in acute and interventional cardiology; Honorary consultant	da134@leicester.ac.uk
13	Nitsch D	1	Professor	dorothea.nitsch@lshtm.ac.uk

For peer review only - http://bmjopen.bmj.com/site/about/guidelines.xhtml

Affiliations:

(1) Department of Non-Communicable Disease Epidemiology, London School of Hygiene & Tropical Medicine, Keppel Street, London UK WC1E 7HT

(2) Population Health Sciences, University of Bristol, Bristol, UK BS8 2PS

(3) Richard Bright Renal Service, North Bristol NHS Trust, Southmead Hospital, Bristol BS10 5NB

(4) Oxford Kidney Unit, Churchill Hospital, Oxford OX3 7LE

(5) Nuffield Department of Medicine, University of Oxford, Oxford, OX3 7BN

(6) Biostatistics Research Group, Department of Health Sciences, University of Leicester, University Road, Leicester, UK, LE1 7RH

(7) National Institute for Cardiovascular Outcomes Research (NICOR), Barts Health NHS Trust, 2nd Floor, 1 St Martin's-Le-Grand, London EC1A 4AS

(8) Institute of Cardiovascular Sciences, University College London. London, UK

(9) Institute of Health Informatics, Faculty of Population Health Sciences, University College London, London UK NW1 2DA

(10) Health Data Research UK, London UK

(11) Glangwili General Hospital, Dolgwili Road, Carmarthen, Wales SA31 2AF

(12) Department of Sciences and NIHR Biomedical Research Centre, University of Leicester, University Road, Leicester UK, LE1 7RH

For peer review only

For peer review only - http://bmjopen.bmj.com/site/about/guidelines.xhtml

Word count (abstract): 300

Word count (main text): 4059

Figures: 1

Tables: 4

Corresponding author: patrick.bidulka1@lshtm.ac.uk

For peer review only

For peer review only - http://bmjopen.bmj.com/site/about/guidelines.xhtml

ABSTRACT

Objectives

To determine: (1) The impact of chronic kidney disease (CKD) severity on the completeness and validity of case ascertainment for hospitalised acute myocardial infarction (AMI) between two secondary care datasets in England; (2) whether risk of death is associated with the dataset in which AMI is recorded; (3) agreement between primary and secondary care estimations of CKD severity.

Methods

A historical cohort study. Primary care records for people with CKD or risk factors for CKD, identified using the National CKD Audit (2015-2017), were linked to the Myocardial Ischaemia National Audit Programme (MINAP, 2007-2017) and Hospital Episode Statistics (HES, 2007-2017) secondary care registries. People with an AMI recorded in either MINAP, HES, or both were included in the study cohort.

Results

We identified 6,748 AMIs. Of these events, 23% were recorded in both MINAP and HES, 66% in HES only and 11% in MINAP only. Compared to people at-risk of CKD or with mild CKD, AMIs in people with moderate-severe CKD were more likely to be recorded in both MINAP and HES (42% vs. 11% respectively), or MINAP only (22% vs. 5%), and less likely to be recorded in HES only (36% vs. 84%). People with AMIs recorded in a HES only or MINAP only had increased odds of death during hospitalisation compared to those recorded in both (adjusted odds ratio (OR)

For peer review only - http://bmjopen.bmj.com/site/about/guidelines.xhtml

1.61, 95% confidence interval (CI) 1.32-1.96 and OR 1.60, 95% CI 1.26-2.04, respectively). Agreement between CKD severity at AMI admission (MINAP) and in primary care was poor (kappa (K) 0.42, standard error (SE) 0.012).

Conclusions

Case ascertainment for AMI hospitalisations is incomplete in both MINAP and HES and is associated with CKD severity. Future observational research in England studying the interaction between cardiovascular and kidney disease should use linked health data to study disease incidence, prevalence, and treatments.

For peer review only

ARTICLE SUMMARY

- Our study includes a large sample size of 6,748 acute myocardial infarction (AMI) events.
- We have assessed the completeness of AMI hospitalisations recorded in two healthcare datasets widely used in observational research in England.
- We evaluated, for the first time, the validity of using serum creatinine recorded in secondary care at the time of an AMI to estimate pre-AMI CKD stage.
- Generalisability to the general population is limited as the National Chronic Kidney Disease Audit only included people with CKD and/or risk factors for CKD.

For peer review only

For peer review only - http://bmjopen.bmj.com/site/about/guidelines.xhtml

INTRODUCTION

Prognosis following acute myocardial infarction (AMI) has improved considerably over the past 50 years such that 85% of individuals now live longer than one year post-AMI.[1] Improved survival is the result of advances in AMI management, driven by evidence from large-scale randomised controlled trials (RCTs).[2-6] Of those admitted to hospital with AMI, 30-40% have chronic kidney disease (CKD):[7] a sustained reduction in kidney function associated with poor outcomes.[8,9] Amongst those with dialysis-dependent CKD only 40% will survive their first year post-AMI.[10] These inferior outcomes may result from higher prevalence of comorbidity,[2] calcific coronary artery disease[3] and the pro-inflammatory effects of uraemia.[4]

Most major RCTs investigating AMI interventions excluded patients with advanced CKD.[11] However, current AMI guidelines from Europe and the United States apply the results of these RCTs to those with or without CKD.[5-7] Clinicians' unease with the dearth of evidence may explain diversion from these AMI guidelines when treating people with CKD.[2,10,11] In the absence of specific RCTs in CKD populations, well-conducted observational analyses can contribute significantly to our understanding and improved management of AMI.

In the UK, data on AMI treatment and outcomes is collected in unlinked, disease-specific registries or in broad registration databases. While there are known differences in the reliability and validity of AMI case ascertainment using these resources in the general population,[12] it is unclear to what extent these differences persist in people with underlying CKD. Multimorbidity and differences in admission and treatment pathways in people with CKD may influence AMI case recording. Reliably identifying which AMI patients have CKD using AMI audit data is also

For peer review only - http://bmjopen.bmj.com/site/about/guidelines.xhtml

difficult; previous studies used admission serum creatinine (SCr) as a proxy for pre-admission CKD stage.[13-15] This unvalidated method risks misclassifying people as having CKD because of the co-incidence of acute kidney injury (AKI) and AMI.[16]

In this study we linked records from the National Chronic Kidney Disease Audit (NCKDA) to the Myocardial Ischaemia National Audit Project (MINAP) and Hospital Episode Statistics (HES) to determine the reliability of these data sources to investigate cardiovascular disease comorbidity and outcomes in people with or at risk of CKD in England. Our objectives were to: (1) Compare case ascertainment of AMI hospitalisations in secondary care datasets (MINAP and HES); (2) determine if MINAP and/or HES case ascertainment defines populations of CKD patients with different risks of death during and after AMI; and (3) compare CKD stage classification using admission serum creatinine recorded in secondary care (MINAP) versus primary care (NCKDA).

For peer review only

METHODS

Data sources

Data from all sources were restricted to patients treated in England. People with or at risk of CKD were identified using primary care data from the NCKDA.[17,18] The NCKDA aimed to optimise the identification and management of people with CKD and/or risk factors for CKD in primary care, and included 10% of English General Practices (GP).[17,18] NCKDA data were collected between 2015 and 2016 in two main cross-sectional data extracts for people with either blood or urine laboratory results indicating CKD and/or risk factors for CKD (prevalent

For peer review only - http://bmjopen.bmj.com/site/about/guidelines.xhtml

hypertension, diabetes mellitus, cardiovascular disease, connective tissue disorders, kidney stones, prostatic disease, family history of kidney disease, previous acute kidney injury (AKI), and users of kidney-damaging medications such as lithium or calcineurin inhibitors).[17] People without an eGFR recorded in primary care were included in the NCKDA only if they were at risk of CKD.

The population identified from NCKDA were linked with Office of National Statistics (ONS) data, January 1998 to September 2019, as well as secondary care data from HES Admitted patient Care (APC) and MINAP, April 2007 to April 2017. HES APC includes hospital admission data for National Health Service (NHS)-treated patients in England, including admission and discharge dates, and diagnoses recorded using International Classification of Diseases 10th Edition (ICD-10) codes.[19] MINAP is an on-going AMI audit in England, Wales, and Northern Ireland which was designed to optimise the care of patients with type one AMI by evaluating the patient pathway from hospital admission with AMI[20] to discharge. MINAP includes admission and discharge dates, treatments, and comorbidities.[21]

Study design

Cohort study.

Study participants

We included people in the NCKDA registered with a GP in England, with one or more AMI hospitalisation recorded in HES or MINAP. People in each NCKDA extract must have been alive according to GP records at the time of that extract. We therefore included people with an AMI

For peer review only - http://bmjopen.bmj.com/site/about/guidelines.xhtml

hospitalisation recorded in MINAP or HES only after the date of their GP's final NCKDA extract. People with an AMI hospitalisation that started prior to the extract date and ended after the extract date were added to the cohort, since they were at risk of death (n=183). In addition, people with an ONS death date indicating death during an AMI hospitalisation that occurred within 90 days prior to the NCKDA extract date were included (n=96), since they were likely misclassified as alive at the time of the extract because of delays in updating the death date in the GP systems. People with an ONS death date earlier than 90 days prior to the extract were excluded (n=4).

Exposures

Our main exposure variable was moderate to severe CKD (eGFR<60mL/min/1.73m^2, CKD stages 3-5), defined using the most recent eGFR recorded in primary care (NCKDA data) prior to the AMI hospitalisation. People with no eGFR recorded in primary care or an eGFR≥60mL/min/1.73m^2 were categorised as at risk of CKD or having mild CKD, respectively. eGFR was calculated using primary care SCr measures and the revised Modification of Diet in Renal Disease (MDRD) equation.[22,23]

Our secondary exposure was CKD stage, defined by the Kidney Disease Improving Global Outcomes CKD staging, based on a single eGFR record without the requirement for two measures three months apart.[24] We combined some CKD stages due to low numbers of AMI cases. Therefore, CKD stage was categorised as (1) At risk of CKD & Stages 1-2 (eGFR missing or

For peer review only - http://bmjopen.bmj.com/site/about/guidelines.xhtml

≥60mL/min/1.73m^2), (2) Stage 3a (eGFR 45-59mL/min/1.73m^2), (3) Stage 3b (eGFR 30-44mL/min/1.73m^2), and (4) Stages 4-5 (eGFR<30mL/min/1.73m^2).

We used the latest eGFR recorded prior to the AMI hospitalisation to categorise people with a history of kidney transplant into the primary and secondary exposure groups. We categorised people with a history of dialysis prior to the AMI hospitalisation into the moderate to severe CKD category (main exposure) and the CKD stages 4-5 category (secondary exposure), even if the latest eGFR did not correspond with these categories.

Outcomes

Primary outcome

The primary outcome was AMI case ascertainment, defined as the dataset(s) in which the AMI hospitalisation was recorded. We defined an AMI as being recorded in both HES and MINAP if an AMI hospitalisation in HES was within 30 days of an AMI hospitalisation in MINAP. Where multiple HES AMI hospitalisations fell within 30 days of a MINAP AMI hospitalisation, the HES AMI hospitalisation closest in time to the MINAP AMI admission was selected as the single matched event. AMI hospitalisations without a match were categorised as HES or MINAP only. Study participants could contribute multiple AMI hospitalisations.

We defined an AMI in HES data using ICD-10 codes I.21, I.22, or I.23 in the primary admission diagnosis field (first diagnostic position in the first episode of an admission).[25] We categorised AMI subtype (ST-segment elevation myocardial infarction (STEMI) and non-ST-segment

For peer review only - http://bmjopen.bmj.com/site/about/guidelines.xhtml

elevation myocardial infarction (NSTEMI)) using the UK Biobank coding definitions[26] (**Supplementary table 1**). We defined AMI in MINAP using a previously developed algorithm which uses the discharge diagnosis, electrocardiogram (ECG) results, and the presence of elevated cardiac markers to identify acute coronary syndrome events and subtypes (**Supplementary table 2**). We excluded MINAP hospitalisations classified as unstable angina or other from the analysis.[12]

Secondary outcomes

We investigated in-hospital mortality during each person's first AMI hospitalisation within the study period. In those who survived and were discharged from their first AMI hospitalisation, we also investigated post-discharge mortality using the ONS death date (up to 15 September 2019). Variables in HES, MINAP, and ONS used to define death are described in **Supplementary table 3**. People were considered to have died during AMI hospitalisation if any of these variables indicated in-hospital death, or the ONS death date fell on or between the admission and discharge dates. We used the earliest of the HES or MINAP admission dates and the latest of the HES or MINAP discharge dates to define these dates for AMI hospitalisations recorded in both datasets.

We investigated the agreement between CKD stage derived from the most recent primary care SCr test (NCKDA data) and CKD stage derived from the secondary care SCr test conducted within 24 hours of AMI hospitalisation (MINAP data). We used the same methods to determine CKD stage in MINAP data as we did for NCKDA data.

For peer review only - http://bmjopen.bmj.com/site/about/guidelines.xhtml

Covariates

We described age at AMI admission (mean and standard deviation (SD) as well as age category in years: 18-49, 50-64, 65-79, 80+), sex, ethnicity (white or other), index of multiple deprivation quintiles (IMD, as a proxy for socioeconomic status), and relevant comorbidities including angina, cerebrovascular disease, chronic obstruction pulmonary disease (COPD), diabetes mellitus (type 1 and 2), heart failure, hypertension, previous myocardial infarction, and peripheral vascular disease. We also described dialysis and transplant status, and smoking status. Data sources for each key covariate are described in **Supplementary table 4**.

Data analysis

Objective 1 – AMI case ascertainment

We summarised key covariates by CKD status. We used Venn diagrams to describe AMI case ascertainment overall and stratified by CKD status (at risk of CKD or mild CKD vs. moderate to severe CKD). We used multinomial, multivariable logistic regression to quantify the association between CKD stage and AMI case ascertainment. We used the 'HES and MINAP' category as the base outcome and reported crude and adjusted relative risk ratios (RRR) and 95% confidence intervals (CI), using 'At risk of CKD & Stages 1-2' as the reference exposure category. We adjusted for sex, age category, ethnicity, IMD quintile, previous AMI, heart failure, COPD, diabetes mellitus, and clustering by participant (using cluster-robust standard errors (SE)). We used a complete case analysis since we could not assume that missing values for ethnicity were

For peer review only - http://bmjopen.bmj.com/site/about/guidelines.xhtml

missing at random. In a secondary analysis, we stratified these regressions by AMI subtype (STEMI and NSTEMI).

Objective 2 – Risk of death

We used multivariable logistic regression to calculate the odds of death in hospital during each person's first AMI hospitalisation in people with AMI recorded in MINAP only or HES only, relative to MINAP and HES. After confirming the proportional hazards assumption with a Schoenfeld Residuals test on the full multivariable model (p=0.35), we used multivariable Cox regression to estimate hazards ratios (HR) for death during total follow-up in those who survived their first AMI hospitalisation with AMI recorded in MINAP only or HES only, relative to MINAP and HES.

Objective 3 – Agreement between CKD staging

Finally, to assess the validity of using MINAP-recorded eGFR at AMI admission as a proxy for pre-admission CKD status, we compared eGFR and its corresponding CKD stage within 24 hours of AMI admission (MINAP data) to the most recent eGFR and its corresponding CKD stage in primary care (NCKDA data). In this analysis, we excluded people with eGFR measures greater than 120mL/min/1.73m^2 in either NCKDA or MINAP as these are unlikely to be true values. We drew a Bland-Altman plot to describe differences in the distribution of eGFR measures in primary and secondary care[27] and calculated the percent agreement and kappa agreement statistics between CKD stage derived using primary and secondary care eGFRs. Secondary analyses re-calculated agreements and kappa statistics restricting to people with CKD Stages

For peer review only - http://bmjopen.bmj.com/site/about/guidelines.xhtml

3a-5 (eGFR<60mL/min/1.73m^2) in primary care and grouped by time between the most recent primary care eGFR measure and the AMI hospitalisation (0-5, 6-11, 12-23, 24-36-month gaps).

Sensitivity analyses

We repeated the main analyses for AMI events occurring prior to the study start date (the latest NCKDA extract). People who experienced AMI hospitalisation before the study start were survivors, since only people alive at the time of the NCKDA were included in the study.

In addition, we re-drew the Venn diagrams after including HES AMI hospitalisations recorded in both the first and second diagnostic positions of the first episode to include AMIs recorded as co-primary diagnoses.[25] We also repeated the matching process between MINAP and HES AMI hospitalisations after combining all HES AMIs within 30 days of each other into a single HES 16hospitalisation, since it is likely that some AMI events in our dataset have multiple HES hospitalisations recorded if, for example, a patient is transferred between hospitals for treatment. Finally, to understand why people may have an AMI hospitalisation recorded in MINAP but not HES, we searched for non-AMI HES hospitalisations within 30 days of the AMI recorded in MINAP and described the ICD-10 diagnoses in the first episode of the first diagnostic position.

For peer review only - http://bmjopen.bmj.com/site/about/guidelines.xhtml

Missing data

We used discharge dates to help re-categorise people who were in-hospital at the time of the NCKDA extract into the cohort, as well as to determine death in hospital and the start of follow-up in those who survived their first AMI hospitalisation. Discharge date was missing in 19% and 1% of the MINAP and HES datasets, respectively. We assumed these dates were missing at random and used the median length of admissions in those without missing admission and discharge dates (5 and 4 days in MINAP and HES, respectively) to impute the missing discharge dates.

Patient and public involvement

The Kidney Care UK patient organisation (https://www.kidneycareuk.org/) supported the research questions, grant applications, and the related record linkage application for section 251 permissions critical to the development of the National Chronic Kidney Disease Audit (NCKDA). Patient members of the UK Renal Registry Patient Council (https://renal.org/patients/patient-council) reviewed the study results. Their feedback supported a further planned record linkage of renal and cardiac data to look at patient outcomes.

For peer review only - http://bmjopen.bmj.com/site/about/guidelines.xhtml

RESULTS

Study population and baseline characteristics

From 1,702,345 people in England included in the NCKDA, we identified 6,042 (0.4%) people with or at risk of CKD who experienced 6,748 AMIs between the final NCKDA extract and 1 April 2017 (**Supplementary figure 1**). Baseline characteristics stratified by CKD stage are described in **Table 1**. People with moderate to severe CKD accounted for 38% of AMI hospitalisations (2,575). Average age at the time of AMI was 73 years (SD 13). People with moderate to severe CKD were older on average than people with mild CKD or at risk of CKD. Most people were white (92%) and male (61%). The most prevalent comorbidities were hypertension (61%) and diabetes mellitus (35%).

Table 1: Baseline characteristics by CKD stage for all AMI events captured after the study start. n (column %) unless specified otherwise.

**CKD status** (main exposure)	**At risk of CKD or mild CKD**	**Moderate to severe CKD**			**Total**
**CKD stage** (secondary exposure)	**At risk of CKD & 1-2**	**3a**	**3b**	**4-5**	
**Unique individuals**	3,751	1,210	732	349	6,042
**Total number of AMI events, N**	4,173	1,353	825	397	6,748
**Age at AMI event, years, mean (SD)**	70 (13)	79 (10)	82 (9)	79 (12)	73 (13)
**Age category at AMI event, years**					
18-50	299 (7)	20 (1)	1 (0)	10 (3)	330 (5)
50-64	1,163 (28)	91 (7)	44 (5)	38 (10)	1,336 (20)
65-79	1,655 (40)	531 (39)	251 (30)	122 (31)	2,559 (38)
80+	1,056 (25)	711 (53)	529 (64)	227 (57)	2,523 (37)
**Female**	1,430 (34)	638 (47)	416 (50)	168 (42)	2,652 (39)
**Ethnicity**					
White	3,807 (91)	1,263 (93)	773 (94)	361 (91)	6,204 (92)
Other	323 (8)	73 (5)	44 (5)	34 (9)	474 (7)
Missing	43 (1)	17 (1)	8 (1)	2 (1)	70 (1)
**IMD quintile**					
1 (least deprived)	732 (18)	252 (19)	152 (18)	68 (17)	1,204 (18)
2	843 (20)	306 (23)	176 (21)	85 (21)	1,410 (21)

For peer review only - http://bmjopen.bmj.com/site/about/guidelines.xhtml

CKD status (main exposure)	At risk of CKD or mild CKD	Moderate to severe CKD			Total
**CKD stage (secondary exposure)**	**At risk of CKD & 1-2**	**3a**	**3b**	**4-5**	
**Unique individuals**	3,751	1,210	732	349	6,042
**Total number of AMI events, N**	4,173	1,353	825	397	6,748
3	934 (22)	331 (24)	193 (23)	79 (20)	1,537 (23)
4	951 (23)	255 (19)	185 (22)	96 (24)	1,487 (22)
5 (most deprived)	690 (17)	206 (15)	114 (14)	69 (17)	1,079 (16)
Missing	23 (1)	3 (0)	5 (1)	0 (0)	31 (0)
**Dialysis in primary care**					
Peritoneal dialysis	0 (0)	0 (0)	0 (0)	15 (4)	15 (0)
Haemodialysis	0 (0)	0 (0)	0 (0)	24 (6)	24 (0)
Renal dialysis, unspecified	0 (0)	0 (0)	0 (0)	7 (2)	7 (0)
**Kidney transplant**	5 (0)	0 (0)	4 (0)	17 (4)	26 (0)
**Comorbidities**					
Angina	959 (23)	399 (29)	275 (33)	155 (39)	1,788 (26)
Cerebrovascular disease	390 (9)	178 (13)	139 (17)	81 (20)	788 (12)
COPD	514 (12)	209 (15)	168 (20)	58 (15)	949 (14)
Diabetes mellitus	1,293 (31)	465 (34)	356 (43)	233 (59)	2,347 (35)
Heart failure	400 (10)	234 (17)	211 (26)	123 (31)	968 (14)
Hypertension	2,333 (56)	884 (65)	583 (71)	322 (81)	4,122 (61)
Myocardial infarction	1,050 (25)	430 (32)	274 (33)	163 (41)	1,917 (28)
Peripheral vascular disease	229 (5)	108 (8)	74 (9)	47 (12)	458 (7)
**Smoking status**					
non-smoker	1,953 (47)	566 (42)	306 (37)	151 (38)	2,976 (44)
ever-smoker	2,018 (48)	530 (39)	318 (39)	140 (35)	3,006 (45)
Missing	202 (5)	257 (19)	201 (24)	106 (27)	766 (11)

AMI: Acute Myocardial Infarction, CKD: chronic kidney disease, COPD: Chronic Obstructive Pulmonary Disease, HES: Hospital Episode Statistics, IMD: Index of Multiple Deprivation, NCKDA: National Chronic Kidney Disease Audit, MINAP: Myocardial Ischaemia National Audit Project

For peer review only

AMI recording in HES and MINAP

Overall, 23% of AMI hospitalisations were captured by both MINAP and HES datasets (1,552 AMI hospitalisations) (**Figure 1**). There was no substantial change in AMI case ascertainment over time (**Supplementary figure 2**). In people with moderate to severe CKD, 42% of all AMI hospitalisations were captured by both MINAP and HES (1,092 AMI hospitalisations). In people with mild CKD or at risk of CKD, 11% of all AMI hospitalisations were captured by MINAP and HES (460 AMI hospitalisations) (**Figure 1**).

For peer review only - http://bmjopen.bmj.com/site/about/guidelines.xhtml

Relative association between CKD stage and AMI recording

Crude and adjusted relative risk ratios (RRR) and 95% confidence intervals (CI) describing the association between CKD stage and AMI case ascertainment are presented in **Table 2**. After adjusting for key covariates, we observed weak evidence of an increased likelihood of AMI recorded in MINAP only, compared to MINAP and HES, in people with CKD stages 4-5 vs. the at risk of CKD/stages 1-2 group (RRR 1.34, 95% CI 0.97-1.85). Furthermore, compared to the at-risk of CKD/stages 1-2 group, people with CKD stages 3a, 3b, and 4-5 were less likely to have an AMI hospitalisation recorded in HES only vs. MINAP and HES. We did not observe any differences in the likelihood of recording of AMI hospitalisation when stratifying by AMI subtype (**Supplementary table 5**).

Table 2: Multinomial logistic regression comparing the relative risk ratios (RRR) of AMI recording across HES and MINAP according to CKD stage. The comparator outcome is people with AMI recorded in both HES and MINAP databases.

**AMI recording** (outcome, compared to people with AMI recorded in MINAP & HES)	**CKD stage** (exposure)	**Number of AMI admissions, n=**	**Unadjusted[1] RR (95% CI)**	**Partially adjusted[2] RRR (95% CI)**	**Adjusted[3] RRR (95% CI)**
**MINAP only** (N=742)	At risk of CKD/Stages 1-2	196	1	1	1
	Stage 3a	245	1.07 (0.85-1.34)	0.98 (0.77-1.25)	0.98 (0.77-1.25)
	Stage 3b	197	1.17 (0.92-1.49)	1.04 (0.80-1.36)	1.03 (0.79-1.34)
	Stages 4-5	104	1.50 (1.11-2.03)	1.38 (1.01-1.90)	1.34 (0.97-1.85)
**HES only** (N=4,367)	At risk of CKD/Stages 1-2	3,456	1	1	1

For peer review only - http://bmjopen.bmj.com/site/about/guidelines.xhtml

	Stage 3a	557	0.14 (0.12-0.16)	0.14 (0.12-0.17)	0.14 (0.12-0.16)
	Stage 3b	224	0.08 (0.06-0.09)	0.08 (0.07-0.10)	0.08 (0.06-0.10)
	Stages 4-5	130	0.11 (0.08-0.14)	0.11 (0.09-0.15)	0.12 (0.09-0.16)

[1]Complete cases for adjusted model
[2]Adjusted for sex, age at AMI admission, ethnicity (white, other), IMD quintile, clustering by participant
[3]Additionally adjusted for comorbidities previous AMI, heart failure, COPD, diabetes mellitus

AMI: Acute Myocardial Infarction, CKD: chronic kidney disease, COPD: Chronic Obstructive Pulmonary Disease, HES: Hospital Episode Statistics, IMD: Index of Multiple Deprivation, MINAP: Myocardial Ischaemia National Audit Project, RRR: relative risk ratio

For peer review only

Mortality during AMI hospitalisation and post-discharge

Of those with a first AMI recorded in both HES and MINAP, 209 people (15%) died during the AMI hospitalisation, compared to 151 (23%) with a first AMI recorded in MINAP only and 579 (15%) recorded in HES only (**Table 3**). After adjusting for key covariates, people with AMI recorded in MINAP only and HES only had increased odds of in-hospital death compared to people with AMI recorded in both MINAP and HES (odds ratio (OR) 1.60, 95% CI 1.26-2.04 and OR 1.61, 95% CI 1.32-1.96, respectively).

Mean follow-up among people who survived a first AMI hospitalisation was 2.4 years. The rate of death per 100 person-years (PY) during complete follow-up was 18.0 (95% CI 16.4-19.7) for AMI recorded in MINAP and HES, 23.3 (95% CI 20.6-26.5) for AMI recorded in MINAP only, and 10.3 (95% CI 9.61-11.0) for AMI recorded in HES only (**Table 3**). After adjusting for key covariates, there was no evidence of a difference in death during follow-up based on which dataset(s) captured the first AMI hospitalisation.

For peer review only - http://bmjopen.bmj.com/site/about/guidelines.xhtml

Table 3: Death during and after first AMI hospitalisation in total study population at risk of or with CKD.

Death during first AMI hospitalisation (N=5,919)[1]	Number who died, n (%)	-	Unadjusted OR (95% CI)	Adjusted[2] OR (95% CI)
MINAP & HES	209 (15)	-	1	1
MINAP only	151 (23)	-	1.67 (1.32-2.11)	1.60 (1.26-2.04)
HES only	579 (15)	-	0.98 (0.82-1.16)	1.61 (1.32-1.96)
**Death during complete follow-up in those who survive first AMI hospitalisation (N=5,009)[1]**	**Number who died during follow-up, n**	**Rate per 100 person-years (95% CI)**	**Unadjusted HR (95% CI)**	**Adjusted[2] HR (95% CI)**
MINAP & HES	456	18.0 (16.4-19.7)	1	1
MINAP only	237	23.3 (20.6-26.5)	1.27 (1.08-1.48)	1.12 (0.96-1.31)
HES only	847	10.3 (9.61-11.0)	0.59 (0.53-0.67)	1.07 (0.94-1.21)

[1]Complete cases for adjusted model
[2]Adjusted for sex, age at AMI admission, ethnicity (white, other), IMD quintile, previous AMI, heart failure, COPD, diabetes mellitus

AMI: Acute Myocardial Infarction, CKD: chronic kidney disease, COPD: Chronic Obstructive Pulmonary Disease, HES: Hospital Episode Statistics, HR: hazard ratio, IMD: Index of Multiple Deprivation, NCKDA: National Chronic Kidney Disease Audit, MINAP: Myocardial Ischaemia National Audit Project, OR: odds ratio

Agreement between eGFR derived from secondary care vs. primary care data

Of the AMI hospitalisations recorded in MINAP, 2,240 (97%) had SCr recorded within 24 hours of AMI admission (**Supplementary table 6**). Median eGFR at time of admission was 47.6 mL/min/1.73m^2 (IQR 33.5-61.6). The Bland-Altman plot comparing the primary care eGFR and

For peer review only - http://bmjopen.bmj.com/site/about/guidelines.xhtml

the secondary care eGFR indicated a negligible mean difference but wide variation (mean difference 3.35 mL/min/1.73m^2, 95% CI -23.4 to 30.1) (**Supplementary figure 3**).

The percent agreements and kappa statistics between CKD stage derived from MINAP eGFR at AMI admission and the CKD stage derived from NCKDA using primary care data are shown in **Table 4**. Overall, there was 57.2% agreement in CKD staging (kappa statistic (K) 0.42 (SE 0.012)). When restricting to people with NCKDA-derived CKD stages 3-5, the % agreement and K indicated worse agreement (**Table 4**). However, when looking at agreement in categorising people as having moderate to severe CKD (stages 3-5) vs mild CKD (stages 1-2), agreement improved (82.1% agreement, K 0.55 (SE 0.021)).

When stratifying by months between the primary and secondary care eGFR measures, we observed the best agreement in CKD staging within a 0-5-month gap between the primary and secondary care eGFR measures: 61.0%, K 0.48 (SE 0.03) (**Table 4**). Agreement was worse when the time between eGFR measures increased.

Table 4: Agreement between primary care-derived CKD stage (NCKDA) and secondary care-derived CKD stage (MINAP)

	**% agreement**	**Kappa statistic (SE)**
**Overall[1]**	57.2%	0.42 (0.012)
**CKD stages 3a, 3b, 4-5[2]**	53.2%	0.34 (0.015)
**CKD stages 1-2, 3a-5[3]**	82.1%	0.55 (0.021)
**Overall[1], by time from NCKDA SCr test (primary care) to MINAP SCr test (at AMI secondary care admission)**		
0-5 months	61.0%	0.48 (0.03)
6-11 months	56.7%	0.42 (0.02)
12-23 months	55.9%	0.40 (0.02)
24-36 months	56.8%	0.41 (0.04)

[1]Overall agreement when grouping as (1) Stages 1-2 (eGFR 60-120mL/min/1.73m^2), (2) Stage 3a (eGFR 45-59mL/min/1.73m^2), (3) Stage 3b (eGFR 30-44 mL/min/1.73m^2), (4) Stages 4-5 (eGFR 0-30 mL/min/1.73m^2)

For peer review only - http://bmjopen.bmj.com/site/about/guidelines.xhtml

[2]Agreement when restricting to people with CKD stages 3a-5, grouped as (1) Stage 3a (eGFR 45-59mL/min/1.73m^2), (2) Stage 3b (eGFR 30-44 mL/min/1.73m^2), (3) Stages 4-5 (eGFR 0-30 mL/min/1.73m^2)
[3]Agreement when grouping as (1) Stages 1-2 (eGFR 60-120mL/min/1.73m^2) and (2) Stages 3a-5 (eGFR 0-59mL/min/1.73m^2)

AMI: acute myocardial infarction, CKD: chronic kidney disease, eGFR: estimated glomerular filtration rate, HES: Hospital Episode Statistics, NCKDA: National Chronic Kidney Disease Audit, MINAP: Myocardial Ischaemia National Audit Project, SCr: serum creatinine, SE: standard error

For peer review only

Sensitivity analyses

AMI case ascertainment in MINAP and HES was similar in AMI hospitalisations recorded prior to the study start (sensitivity analysis) compared to after the study start (main analysis) (**Supplementary tables 7-8, Supplementary figure 4**). There were also no major differences in agreement between CKD staging derived in primary vs. secondary care when investigating AMI hospitalisations prior to the study start (**Supplementary table 9**).

After expanding the AMI definition in HES to include any hospitalisations with AMI coded in the second diagnostic position as well as the first, the proportion of AMI hospitalisations captured in both HES and MINAP decreased slightly (**Supplementary figure 5**). After combining HES AMI admissions within 30 days of each other for the same person, we observed a 1% increase in the proportion of AMI hospitalisations recorded in both MINAP and HES (**Supplementary figure 5**).

Finally, the ten most common diagnoses in HES matching with the MINAP only AMI hospitalisations from the main analysis are shown in **Supplementary table 10**. Eighty-eight percent of unmatched MINAP AMIs had a non-AMI HES hospitalisation within 30 days. These were mainly CVD or respiratory infection-related ICD-10 diagnoses.

For peer review only - http://bmjopen.bmj.com/site/about/guidelines.xhtml

DISCUSSION

We compared recording of AMI hospitalisations for people with CKD between two large secondary healthcare datasets in England. In a cohort of 6,042 people, we found that both HES and MINAP missed a significant proportion of AMI hospitalisations. CKD stage influenced likelihood of AMI recording by dataset: AMI hospitalisations in people with moderate to severe CKD were more likely to be recorded in MINAP compared to people at risk of CKD or with mild CKD. We found an association between AMI hospitalisation recording by dataset and in-hospital mortality. CKD stage calculated from SCr at the time of AMI admission (recorded in MINAP) varied significantly from that derived from SCr recorded in primary care.

Our results agree with previous research demonstrating incomplete capture of AMI events by individual healthcare datasets in the overall English population and extend them to a population with CKD. Herrett et al[12] showed 46% agreement when restricting to MINAP and HES recorded AMI hospitalisations, which is close to the 42% agreement we found in people with moderate to severe CKD. A smaller single-centre study by Torabi et al (2005) found 32% agreement between MINAP and the hospital information department (responsible for HES coding).

In contrast to both studies, we found significantly worse agreement in case ascertainment for AMI hospitalisations between MINAP and HES for people at risk of CKD or with mild CKD. Torabi collected data on renal function but found likelihood of AMI case recording in MINAP to reduce

For peer review only - http://bmjopen.bmj.com/site/about/guidelines.xhtml

with advancing CKD stage. Differences in results between this and our study could be ascribed to changes in management of patients and/or event recording over time, differences in the populations studied and local practice in the single centre analysed by Torabi et al.

The high prevalence of CKD risk factors in people at risk of or with mild CKD could put them at greater risk of type 2 AMI; a mismatch of myocardial oxygen supply and demand in the absence of the "classical" coronary artery plaque rupture with thrombosis reflective of type 1 AMI.[20] People with type 2 AMI are typically older, with a greater burden of comorbidities than those with type 1 AMI, and have poor outcomes.[28] HES is likely to include more type 2 AMI than MINAP as clinical coders for the latter are asked to select type 1 AMI only.

People with AMI recorded in both MINAP and HES had lower in-hospital mortality compared with those with AMI recorded in either MINAP or HES only. Our findings agree with Herrett et al;[12] patients with AMI recorded in only one source had a higher mortality than those with events recorded in more than one source. Higher in-hospital mortality in the MINAP only cases is likely to reflect the referral of severe and complex AMI cases to cardiology, including a higher STEMI to NSTEMI ratio.

We found significant variation between CKD stage derived from SCr taken within 24 hours of AMI admission (recorded in MINAP) and that derived from SCr in primary care. Agreement was lowest for people with moderate to severe CKD. These findings suggest that although previous research[13-15] using SCr at AMI admission recorded in MINAP as a proxy for baseline CKD stage may misclassify CKD stage, it is unlikely to have resulted in a systematic bias in either over- or under-estimation of CKD stage. Differences between SCr recorded in primary care and SCr

For peer review only - http://bmjopen.bmj.com/site/about/guidelines.xhtml

recorded in MINAP may reflect progression of CKD, differential use of medication that affects the renin-angiotension-aldosterone system, AKI at the time of serum sampling (although changes in serum creatinine are unlikely to show within 24 hours of AMI onset), or variation around the mean. Binary classification between individuals with CKD stages 3-5 and those with stages 1-2 is more reliable than classification by CKD stage.

Limitations

The NCKDA only included people with CKD and/or risk factors for CKD; therefore, we cannot generalise our results to the general population. Secondly, people with CKD but without a recorded SCr value in primary care may have been excluded from this study or incorrectly assumed to have risk factors for CKD only. Our results are likely impacted by residual confounding, since we were limited in the number of relevant comorbidities we could include in our multivariable models. Finally, we may have missed AMI cases by including only those recorded in the first diagnostic position of the first episode of a HES admission; however, our sensitivity analysis which included AMI hospitalisations recorded in the first or second diagnostic position showed similar results. Inclusion of the first diagnostic position of later episodes was undertaken in a similar study investigating AMI case ascertainment in people with malignancy, with little improvement in agreement between datasets.[29]

Future research

This study demonstrates how AMI case ascertainment can be improved by using linked healthcare datasets. Further research investigating disease incidence, prevalence and outcomes should follow this approach. Optimising data quality in UK healthcare datasets and simplifying

For peer review only - http://bmjopen.bmj.com/site/about/guidelines.xhtml

the process of data linkage would facilitate high-quality observational research to inform the design of future RCTs and provide estimated treatment effects where RCT data are lacking.

Conclusion

The use of linked healthcare datasets should be prioritised in observational research investigating multimorbidity.

For peer review only

For peer review only - http://bmjopen.bmj.com/site/about/guidelines.xhtml

ACKNOWLEDGEMENTS

MINAP is commissioned, as part of the 'National Cardiac Audit Project' by the Healthcare Quality Improvement Partnership. This work uses data that has been provided by patients and collected by the NHS as part of their care and support.

We acknowledge and thank UK cardiovascular, renal, and primary care physicians as well as hospital and community audit and coding teams whose diligent data collection has provided the data for these analyses. In particular, we would like to thank James Chal, Anil Gunesh, Andrew Harrison, and Akosua Donkor from NICOR; and Kathryn Griffith, Matthew Harker, Yvonne Silove, Tasneem Hoosain, Nick Wilson, Ronnie Moodley, Richard Fluck, Chris Gush, David Wheeler, Liam Smeeth, Ron Cullen, Andy Syme, Richard Gunn, Paul Wright, Hugh Gallagher, Sion Edwards, Fiona Loud, Nick Palmer, Richard Fluck, Anita Sharma, Kate Cheema, Andy Syme, Sally Hull, Ben Caplin, Lois Kim, and Faye Cleary from the NCKDA steering, clinical reference, and statistical groups.

AUTHOR CONTRIBUTIONS

JS, DMT, DN, DA, FC, and UU conceived the study. JS, DMT, DN, DA, FC, UU, and PB designed the study. PB and DN had access to and analysed the data. PB and JS drafted the manuscript. All authors critically appraised the results and edited the manuscript. All authors approved the

For peer review only - http://bmjopen.bmj.com/site/about/guidelines.xhtml

final version of the manuscript. The lead authors confirm all authors meet ICJME authorship criteria, and no one who meets ICJME criteria have been excluded.

COMPETING INTERESTS

All authors have completed an ICJME form. PB, JS, DMT, FC, LT, MDB, and SD have nothing to declare. UU declares a grant from the Health Foundation to undertake quality improvement unrelated to this work. MS declares funding from Cancer Research UK (C53325/A21134) and British Heart Foundation (SP/16/5/32415) for research activities related to this work looking at acute myocardial infarction ascertainment in a cancer population. JS declares Doctoral research funding from the NIHR for related research looking at acute myocardial infarction care for people with chronic kidney disease. JD declares grants from British Heart Foundation paid to his institution unrelated to this work. JD also declares consulting fees from Novo Nordisk, and honoraria from Amgen, Boehringer Ingelheim, Merck, Pfizer, Aegerion, Novartis, Sanofi, Takeda, Novo Nordisk, and Bayer, unrelated to this work. JD is also member of a study steering committee with Novo Nordisk. CW declares he is clinical lead of the Myocardial Ischaemia National Audit Project. DA reports research funding and in-kind support from Astra Zeneca inc. for unrelated research and educational funding from Abbott Vascular inc. to support a clinical research fellow doing unrelated research. DA has conducted consultancy for General Electric inc. to support general research funds. DN is the UK Kidney Association Director of Informatics Research. DN is also on the steering group for two GlaxoSmithKline funded studies that investigate kidney function in children and adults in Sub-Saharan Africa.

For peer review only - http://bmjopen.bmj.com/site/about/guidelines.xhtml

FUNDING

This work was supported by Kidney Research UK (grant number IN_008_20180304) and the Health Foundation (grant number 1725841). JS is a Doctoral Research Fellow funded by the NIHR (NIHR 300906). DA is funded by a joint research grant from the British Heart Foundation (SP/16/5/32415) and Cancer Research UK (C53325/A21134).

DATA SHARING

Due to data sharing agreements, we are not able to share these data. Researchers interested in using these data should consult the websites for the NCKDA (https://www.lshtm.ac.uk/research/centres-projects-groups/ckdaudit#welcome) and MINAP (https://www.nicor.org.uk/national-cardiac-audit-programme/myocardial-ischaemia-minap-heart-attack-audit/).

ETHICS APPROVAL

This study was approved by the London School of Hygiene & Tropical Medicine LEO ethics committee (ID 16988) and the National Chronic Kidney Disease Audit (NCKDA) Steering Committee. Audits used in this study are covered by Section 251 approvals (NHS Act 2006)

For peer review only - http://bmjopen.bmj.com/site/about/guidelines.xhtml

For peer review only

which allows data to be collected without individual patient consent for medical research when it is not possible to use anonymised information and when seeking consent is not practical.

REFERENCES

1. Foundation BH. Heart statistics. https://www.bhf.org.uk/what-we-do/our-research/heart-statistics. Published 2020. Accessed 28/02/2020, 2020.
2. Pitt B, Remme W, Zannad F, et al. Eplerenone, a selective aldosterone blocker, in patients with left ventricular dysfunction after myocardial infarction. *The New England journal of medicine.* 2003;348(14):1309-1321.
3. Januzzi JL, Cannon CP, DiBattiste PM, Murphy S, Weintraub W, Braunwald E. Effects of renal insufficiency on early invasive management in patients with acute coronary syndromes (The TACTICS-TIMI 18 Trial). *The American journal of cardiology.* 2002;90(11):1246-1249.
4. Bhatt DL, Roe MT, Peterson ED, et al. Utilization of early invasive management strategies for high-risk patients with non-ST-segment elevation acute coronary syndromes: results from the CRUSADE Quality Improvement Initiative. *Jama.* 2004;292(17):2096-2104.
5. Kedhi E, Fabris E, van der Ent M, et al. Six months versus 12 months dual antiplatelet therapy after drug-eluting stent implantation in ST-elevation myocardial infarction (DAPT-STEMI): randomised, multicentre, non-inferiority trial. *BMJ (Clinical research ed).* 2018;363:k3793.
6. Serruys PW, Kogame N, Katagiri Y, et al. Clinical outcomes of state-of-the-art percutaneous coronary revascularisation in patients with three-vessel disease: two-year follow-up of the SYNTAX II study. *EuroIntervention.* 2019;15(3):e244-e252.
7. Wright RS, Reeder GS, Herzog CA, et al. Acute myocardial infarction and renal dysfunction: a high-risk combination. *Ann Intern Med.* 2002;137(7):563-570.
8. Santopinto JJ, Fox KA, Goldberg RJ, et al. Creatinine clearance and adverse hospital outcomes in patients with acute coronary syndromes: findings from the global registry of acute coronary events (GRACE). *Heart.* 2003;89(9):1003-1008.
9. Fox CS, Muntner P, Chen AY, et al. Use of evidence-based therapies in short-term outcomes of ST-segment elevation myocardial infarction and non-ST-segment elevation myocardial infarction in patients with chronic kidney disease: a report from the National Cardiovascular Data Acute Coronary Treatment and Intervention Outcomes Network registry. *Circulation.* 2010;121(3):357-365.
10. Herzog CA, Ma JZ, Collins AJ. Poor long-term survival after acute myocardial infarction among patients on long-term dialysis. *The New England journal of medicine.* 1998;339(12):799-805.

For peer review only - http://bmjopen.bmj.com/site/about/guidelines.xhtml

11. Konstantinidis I, Nadkarni GN, Yacoub R, et al. Representation of Patients With Kidney Disease in Trials of Cardiovascular Interventions: An Updated Systematic Review. *JAMA internal medicine.* 2016;176(1):121-124.
12. Herrett E, Shah AD, Boggon R, et al. Completeness and diagnostic validity of recording acute myocardial infarction events in primary care, hospital care, disease registry, and national mortality records: cohort study. *BMJ (Clinical research ed).* 2013;346:f2350.
13. Gupta T, Paul N, Kolte D, et al. Association of chronic renal insufficiency with in-hospital outcomes after percutaneous coronary intervention. *J Am Heart Assoc.* 2015;4(6):e002069.
14. Rozenbaum Z, Benchetrit S, Minha S, et al. The Effect of Admission Renal Function on the Treatment and Outcome of Patients with Acute Coronary Syndrome. *Cardiorenal Med.* 2017;7(3):169-178.
15. Shaw C, Nitsch D, Steenkamp R, et al. Inpatient coronary angiography and revascularisation following non-ST-elevation acute coronary syndrome in patients with renal impairment: a cohort study using the Myocardial Ischaemia National Audit Project. *PLoS One.* 2014;9(6):e99925.
16. Grams ME, Astor BC, Bash LD, Matsushita K, Wang Y, Coresh J. Albuminuria and estimated glomerular filtration rate independently associate with acute kidney injury. *J Am Soc Nephrol.* 2010;21(10):1757-1764.
17. Nitsch D CB HS, Wheeler D. National Chronic Kidney Disease Audit - National Report Part 1. 2017.
18. Mudie K CF CB, Wheeler D, Hull S, Nitsch D. National Chronic Kidney Disease Audit: National Report Part 2. 2017.
19. Hospital Episode Statistics. https://digital.nhs.uk/data-and-information/data-tools-and-services/data-services/hospital-episode-statistics. Published 2019. Accessed2020.
20. Thygesen K, Alpert JS, Jaffe AS, et al. Fourth universal definition of myocardial infarction (2018). *Eur Heart J.* 2018.
21. audit) MIMHA. https://www.nicor.org.uk/national-cardiac-audit-programme/myocardial-ischaemia-minap-heart-attack-audit/, . Published 2020. Accessed.
22. Levey AS, Coresh J, Greene T, et al. Using standardized serum creatinine values in the modification of diet in renal disease study equation for estimating glomerular filtration rate. *Ann Intern Med.* 2006;145(4):247-254.
23. Levey AS, Stevens LA, Schmid CH, et al. A new equation to estimate glomerular filtration rate. *Ann Intern Med.* 2009;150(9):604-612.
24. Stevens PE, Levin A. Evaluation and management of chronic kidney disease: synopsis of the kidney disease: improving global outcomes 2012 clinical practice guideline. *Ann Intern Med.* 2013;158(11):825-830.
25. Herbert A, Wijlaars L, Zylbersztejn A, Cromwell D, Hardelid P. Data Resource Profile: Hospital Episode Statistics Admitted Patient Care (HES APC). *Int J Epidemiol.* 2017;46(4):1093-1093i.
26. Schnier C BK NJ, Sudlow C. Definitions of Acute Myocardial Infarction and Main Myocardial Infarction Pathological Types - UK Biobank Phase 1 Outcomes Adjudication.

For peer review only - http://bmjopen.bmj.com/site/about/guidelines.xhtml

https://biobank.ndph.ox.ac.uk/showcase/showcase/docs/alg_outcome_mi.pdf. Published 2017. Accessed 12 January, 2021.

27. Giavarina D. Understanding Bland Altman analysis. *Biochem Med (Zagreb).* 2015;25(2):141-151.

28. Baron T, Hambraeus K, Sundström J, Erlinge D, Jernberg T, Lindahl B. Type 2 myocardial infarction in clinical practice. *Heart.* 2015;101(2):101-106.

29. Coles B, Teece L, Weston C, et al. Case-ascertainment of acute myocardial infarction hospitalizations in cancer patients: a cohort study using English linked electronic health data. *European Heart Journal - Quality of Care and Clinical Outcomes.* 2021.

For peer review only

For peer review only - http://bmjopen.bmj.com/site/about/guidelines.xhtml

FIGURE LEGEND

Figure 1: Venn diagrams illustrating AMI recording in MINAP and HES secondary care datasets. Venn diagrams presented overall, and stratified by CKD status (at-risk of or mild CKD, eGFR≥60mL/min/1.73m^2 or moderate to severe CKD, eGFR<60 mL/min/1.73m^2). Circle areas are proportional to the number of AMI events in each dataset.

TABLE LEGEND

Table 1: Baseline characteristics by CKD stage for all AMI events captured after the study start. n (column %) unless specified otherwise.

Table 2: Multinomial logistic regression comparing the relative risk ratios (RRR) of AMI recording across HES and MINAP according to CKD stage. The comparator outcome is people with AMI recorded in both HES and MINAP databases.

Table 3: Death during and after first AMI hospitalisation in total study population at risk of or with CKD.

Table 4: Agreement between primary care-derived CKD stage (NCKDA) and secondary care-derived CKD stage (MINAP)

For peer review only

For peer review only - http://bmjopen.bmj.com/site/about/guidelines.xhtml

Supplementary materials

Supplementary tables

Supplementary table 1: ICD-10 codes for AMI identified in HES

Supplementary table 2: CALIBER definition of AMI subtypes (STEMI, NSTEMI) using MINAP data.

Supplementary table 3: Variables used to define death in-hospital and post-AMI discharge.

Supplementary table 4: Details on data sources for covariates.

Supplementary table 5: AMI database capture by CKD status (binary) stratified by AMI subtype (STEMI or NSTEMI)

Supplementary table 6: Serum creatinine (SCr) measured in the first 24 hours of AMI hospitalization recorded in MINAP, stratified by CKD stage defined in primary care (NCKDA)

Supplementary table 7: AMI hospitalisations occurring before study start – description of people in the NCKDA who experienced AMI hospitalisation recorded in MINAP or HES before the study start. n (column %) unless specified otherwise.

Supplementary table 8: AMI hospitalisations occurring before study start - multinomial logistic regression comparing the relative risk ratios (RRR) of AMI recording across HES and MINAP according to CKD stage. The comparator outcome is people with AMI recorded in both HES and MINAP databases.

Supplementary table 9: AMI hospitalisations occurring before study start - agreement between primary care-derived CKD stage (NCKDA) and secondary care-derived CKD stage (MINAP)

Supplementary table 10: Sensitivity analysis describing the ICD-10 diagnoses (first episode, first diagnostic position) of non-AMI HES cases that are within ±30 days of MINAP only AMI cases from the main analyses

Supplementary figures

Supplementary figure 1: Flow diagram illustrating the NCKDA extracts, and resulting contribution of AMI events to this study

Supplementary figure 2: AMI case ascertainment in MINAP and HES datasets over time.

Supplementary figure 3: Bland-Altman plot comparing the mean eGFR (NCKDA eGFR and MINAP eGFR, x-axis) and the difference between the NCKDA eGFR and the MINAP eGFR (y-axis).

For peer review only

Supplementary figure 4: AMI hospitalisations occurring before study start - comparing AMI case ascertainment overall and by CKD status for AMI hospitalisations in the main analysis and AMI hospitalisations which occurred prior to the last NCKDA extract (sensitivity analysis).

Supplementary figure 5: Sensitivity analyses compared to main analysis, describing AMI recording in MINAP and HES in the overall study population at risk of or with CKD. Areas of Venn diagrams are proportional to the number of cases.

For peer review only

Supplementary table 1: ICD-10 codes for AMI identified in HES

**AMI subtype**	**ICD-10 codes**
Overall	I21, I22, I23
STEMI	I21.0, I21.1, I21.2, I21.3, I22.0, I22.1, I22.8
NSTEMI	I21.4, I21.9, I22.9
Missing	I23

AMI: acute myocardial infarction; HES: Hospital Episode Statistics; ICD-10: International Classification of Diseases 10th Edition; STEMI: ST elevated myocardial infarction; NSTEMI: Non-ST elevated myocardial infarction

For peer review only

Supplementary table 2: CALIBER definition of AMI subtypes (STEMI, NSTEMI) using MINAP data.

	MINAP variable		
**AMI subtype**	**Discharge diagnosis**	**Markers elevated?**	**ECG result**
Other	Threatened MI, Chest pain uncertain cause, MI unconfirmed, other diagnosis	-	-
STEMI	STEMI	Raised or missing	ST elevation, LBBB, or ST elevation
	NSTEMI/Troponin positive ACS	Raised or missing	ST elevation
	ACS troponin negative	Raised	ST elevation
	ACS troponin unspecified	Raised	ST elevation
NSTEMI	NSTEMI/Troponin positive ACS	Raised or missing	ST depression, T wave changes only, other abnormality, Normal ECG, or LBBB
	ACS troponin negative	Raised	LBBB, ST depression, T wave changes only, Other abnormality, normal ECG, or missing
	ACS troponin unspecified	Raised	LBBB, ST depression, T wave changes only, Other abnormality, normal ECG, or missing
Unstable angina	*Any remaining hospitalisations not assigned as STEMI, NSTEMI, or other diagnosis		

ACS: acute coronary syndrome, AMI: acute myocardial infarction, ECG: electrocardiogram, LBBB: left bundle branch block, MI: myocardial infarction, MINAP: Myocardial Ischaemia National Audit Project, NSTEMI: Non ST-elevation myocardial infarction, STEMI: ST-elevation myocardial infarction

For peer review only

Supplementary table 3: Variables used to define death in-hospital and post-AMI discharge.

**Data source**	**Variables to define in-hospital death**	**Variables to define death post-AMI discharge (in those who survive first AMI hospitalization)**
ONS	Date of death (falling on or in between first AMI admission and discharge dates)	Date of death (falling after first AMI discharge date)
MINAP	Reason for no angiography, no intervention, admission ward, discharge destination	-
HES	Discharge location, discharge method	-

AMI: acute myocardial infarction, HES: Hospital Episode Statistics, MINAP: Myocardial Ischaemia National Audit Project, ONS: Office of National Statistics

For peer review only

Supplementary table 4: Details on data sources for covariates.

Category	Covariate	Data source
Sociodemographic and lifestyle variables	Age at AMI admission	NCKDA
	Sex	NCKDA
	IMD quintiles	NCKDA
	Smoking status	NCKDA, MINAP
Comorbidities	Angina	MINAP and HES
	Cerebrovascular disease	MINAP and HES
	COPD	MINAP and HES
	Diabetes mellitus	MINAP and HES
	Heart failure	MINAP and HES
	Hypertension	NCKDA, MINAP, and HES
	Previous myocardial infarction	MINAP, and HES
	Peripheral vascular disease	NCKDA, MINAP, and HES
	Dialysis	NCKDA
	Kidney transplant	NCKDA

AMI: Acute Myocardial Infarction, COPD: Chronic Obstructive Pulmonary Disease, HES: Hospital Episode Statistics, IMD: Index of Multiple Deprivation, NCKDA: National Chronic Kidney Disease Audit, MINAP: Myocardial Ischaemia National Audit Project

For peer review only

Supplementary table 5: AMI database capture by CKD status (binary) stratified by AMI subtype (STEMI or NSTEMI)

AMI subtype	Database (outcome, compared to people with AMI recorded in MINAP & HES)	CKD stage (exposure)	Number of AMI admissions	Unadjusted RRR (95% CI)	Adjusted RRR[1] (95% CI)
**STEMI**	**MINAP only** (N=115)	At risk of CKD & Stages 1-2	31	1	1
		Stage 3a-5	74	1.08 (0.68-1.72)	0.88 (0.52-1.49)
	**HES only** (N=1,482)	At risk of CKD & Stages 1-2	1,306	1	1
		Stage 3a-5	194	0.07 (0.05-0.09)	0.07 (0.05-0.09)
**NSTEMI**	**MINAP only** (N=666)	At risk of CKD & Stages 1-2	167	1	1
		Stage 3a-5	474	1.16 (0.93-1.45)	1.11 (0.88-1.41)
	**HES only** (N=2,834)	At risk of CKD & Stages 1-2	2,164	1	1
		Stage 3a-5	717	0.14 (0.12-0.16)	0.15 (0.12-0.17)

[1] adjusted for sex, age group at AMI admission, ethnicity, previous AMI, and clustering by patient

AMI: Acute Myocardial Infarction, CKD: chronic kidney disease, HES: Hospital Episode Statistics, MINAP: Myocardial Ischaemia National Audit Project, NSTEMI: non-ST elevation myocardial infarction, RRR: relative risk ratio, STEMI: ST-elevation myocardial infarction

For peer review only

Supplementary table 6: Serum creatinine (SCr) measured in the first 24 hours of AMI hospitalization recorded in MINAP, stratified by CKD stage defined in primary care (NCKDA)

**CKD status (NCKDA)**	**SCr recorded in MINAP at AMI admission, n (%)**	**MINAP median SCr, umol (IQR)**	**MINAP Median SCr value*, mg/dL (IQR)**	**MINAP Median eGFR, mL/min/1.73m^2 (IQR)**
All stages	2,240 (97)	115 (91-152)	1.30 (1.03-1.72)	47.6 (33.5-61.6)
At risk of CKD & Stages 1-2	630 (97)	88 (74-104)	1.00 (0.84-1.18)	67.0 (56.0-80.2)
3a	770 (97)	109 (94-128)	1.23 (1.06-1.45)	50.8 (41.9-59.2)
3b	587 (98)	140 (117-174)	1.58 (1.32-1.97)	36.7 (29.4-43.4)
4-5	253 (95)	255 (190-407)	2.88 (2.15-4.60)	19.8 (12.0-25.5)

*Multiplied by 0.95 for SCr taken before 2013

AMI: acute myocardial infarction, CKD: chronic kidney disease, eGFR: estimated glomerular filtration rate, NCKDA: National Chronic Kidney Disease Audit, MINAP: Myocardial Ischaemia National Audit Project, SCr: serum creatinine

For peer review only

Supplementary table 7: AMI hospitalisations occurring before study start – description of people in the NCKDA who experienced AMI hospitalisation recorded in MINAP or HES before the study start. n (column %) unless specified otherwise.

**CKD status** (main exposure)	**No CKD**	**CKD**			**Total**
**CKD stage** (secondary exposure)	**At-risk of CKD & 1-2**	**3a**	**3b**	**4-5**	
**Unique individuals**	27,909	4,884	2,083	726	34,860
**Total number of AMI events, N**	32,041	5,641	2,460	926	41,068
**Age at AMI event, years, mean (SD)**	64 (13)	75 (10)	78 (10)	75 (12)	66 (13)
**Age category at AMI event, years**					
18-50	4,506 (14)	83 (1)	22 (1)	34 (4)	4,645 (11)
50-64	11,964 (37)	829 (15)	207 (8)	130 (14)	13,130 (32)
65-79	11,517 (36)	2,777 (49)	986 (40)	405 (44)	15,685 (38)
80+	3,922 (12)	1,952 (35)	1,245 (51)	357 (39)	7,476 (18)
Missing	132 (0)	0 (0)	0 (0)	0 (0)	132 (0)
**Female**	9,288 (29)	2,482 (44)	1,209 (49)	394 (43)	13,373 (33)
**Ethnicity**					
White	29,016 (91)	5,228 (93)	2,257 (92)	846 (91)	37,347 (91)
Other	2,612 (8)	295 (5)	139 (6)	71 (8)	3,117 (8)
Missing	413 (1)	118 (2)	64 (3)	9 (1)	604 (1)
**IMD quintile**					
1 (least deprived)	5,808 (18)	1,121 (20)	456 (19)	184 (20)	7,569 (18)
2	6,716 (21)	1,284 (23)	523 (21)	182 (20)	8,705 (21)
3	6,922 (22)	1,297 (23)	542 (22)	180 (19)	8,941 (22)
4	6,820 (21)	1,134 (20)	525 (21)	216 (23)	8,695 (21)
5 (most deprived)	5,531 (17)	787 (14)	406 (17)	161 (17)	6,885 (17)
Missing	244 (1)	18 (0)	8 (0)	3 (0)	273 (1)
**Dialysis in primary care**					
Peritoneal dialysis	0 (0)	0 (0)	0 (0)	12 (1)	12 (0)
Haemodialysis	0 (0)	0 (0)	0 (0)	24 (3)	24 (0)
Renal dialysis, unspecified	0 (0)	0 (0)	0 (0)	11 (1)	11 (0)
**Kidney transplant**	3 (0)	4 (0)	8 (0)	11 (1)	26 (0)
**Comorbidities**					
Angina	5,001 (16)	1,630 (29)	882 (36)	335 (36)	7,848 (19)
Cerebrovascular disease	1,405 (4)	497 (9)	309 (13)	135 (15)	2,346 (6)

For peer review only

CKD status (main exposure)	No CKD	CKD			Total
**CKD stage (secondary exposure)**	**At-risk of CKD & 1-2**	**3a**	**3b**	**4-5**	
**Unique individuals**	27,909	4,884	2,083	726	34,860
**Total number of AMI events, N**	32,041	5,641	2,460	926	41,068
COPD	2,059 (6)	680 (12)	368 (15)	147 (16)	3,254 (8)
Diabetes	5,823 (18)	1,500 (27)	873 (35)	463 (50)	8,659 (21)
Heart failure	1,228 (4)	456 (8)	353 (14)	199 (21)	2,236 (5)
Hypertension	10,556 (33)	3,287 (58)	1,721 (70)	673 (73)	16,237 (40)
Myocardial infarction	5,563 (17)	1,510 (27)	832 (34)	380 (41)	8,285 (20)
Peripheral vascular disease	799 (2)	258 (5)	181 (7)	106 (11)	1,344 (3)
**Smoking status**					
non-smoker	12,945 (40)	2,049 (36)	835 (34)	301 (33)	16,130 (39)
ever-smoker	16,626 (52)	2,477 (44)	983 (40)	382 (41)	20,468 (50)
Missing	2,470 (8)	1,115 (20)	642 (26)	243 (26)	4,470 (11)

AMI: Acute Myocardial Infarction, CKD: chronic kidney disease, COPD: Chronic Obstructive Pulmonary Disease, HES: Hospital Episode Statistics, IMD: Index of Multiple Deprivation, NCKDA: National Chronic Kidney Disease Audit, MINAP: Myocardial Ischaemia National Audit Project

For peer review only

Supplementary table 8: AMI hospitalisations occurring before study start - multinomial logistic regression comparing the relative risk ratios (RRR) of AMI recording across HES and MINAP according to CKD stage. The comparator outcome is people with AMI recorded in both HES and MINAP databases.

**AMI recording** (outcome, compared to people with AMI recorded in MINAP & HES)	**CKD stage** (exposure)	**Number of AMI admissions, n=**	**Unadjusted[1] RRR (95% CI)**	**Partially adjusted[2] RRR (95% CI)**	**Adjusted[3] RRR (95% CI)**
**MINAP only** (N=3,957)	At-risk/Stages 1-2	2,147	1	1	1
	Stage 3a	999	0.96 (0.87-1.05)	0.90 (0.82-0.99)	0.88 (0.80-0.97)
	Stage 3b	578	1.22 (1.09-1.36)	1.12 (0.99-1.26)	1.05 (0.93-1.19)
	Stages 4-5	233	1.35 (1.14-1.60)	1.27 (1.07-1.51)	1.13 (0.94-1.35)
**HES only** (N=26,697)	At-risk/Stages 1-2	23,904	1	1	1
	Stage 3a	1,911	0.16 (0.15-0.18)	0.23 (0.22-0.25)	0.24 (0.22-0.25)
	Stage 3b	630	0.12 (0.11-0.13)	0.20 (0.17-0.22)	0.21 (0.18-0.23)
	Stages 4-5	252	0.13 (0.11-0.15)	0.18 (0.15-0.22)	0.20 (0.17-0.24)

[1]Complete cases for adjusted model
[2]Adjusted for sex, age at AMI admission, ethnicity (white, other), IMD quintile, clustering by participant
[3]Additionally adjusted for comorbidities previous AMI, heart failure, COPD, diabetes mellitus

AMI: Acute Myocardial Infarction, CKD: chronic kidney disease, COPD: Chronic Obstructive Pulmonary Disease, HES: Hospital Episode Statistics, IMD: Index of Multiple Deprivation, MINAP: Myocardial Ischaemia National Audit Project, RRR: relative risk ratio

For peer review only

Supplementary table 9: AMI hospitalisations occurring before study start - agreement between primary care-derived CKD stage (NCKDA) and secondary care-derived CKD stage (MINAP)

	**% agreement**	**Kappa statistic (SE)**
**Overall[1]**	66.4%	0.51 (0.007)
**CKD stages 3a, 3b, 4-5[2]**	57.8%	0.37 (0.008)
**CKD stages 1-2, 3a-5[3]**	80.3%	0.59 (0.010)
**Overall[1], by time from NCKDA SCr test (primary care) to MINAP SCr test (at AMI secondary care admission)**		
0-5 months	66.7%	0.52 (0.009)
6-11 months	65.6%	0.49 (0.013)
12-23 months	63.3%	0.42 (0.023)
24-36 months	65.0%	0.45 (0.034)

[1]Overall agreement when grouping as (1) Stages 1-2 (eGFR 60-120mL/min/1.73m^2), (2) Stage 3a (eGFR 45-59mL/min/1.73m^2), (3) Stage 3b (eGFR 30-44 mL/min/1.73m^2), (4) Stages 4-5 (eGFR 0-30 mL/min/1.73m^2)
[2]Agreement when restricting to people with CKD stages 3a-5, grouped as (1) Stage 3a (eGFR 45-59mL/min/1.73m^2), (2) Stage 3b (eGFR 30-44 mL/min/1.73m^2), (3) Stages 4-5 (eGFR 0-30 mL/min/1.73m^2)
[3]Agreement when grouping as (1) Stages 1-2 (eGFR 60-120mL/min/1.73m^2) and (2) Stages 3a-5 (eGFR 0-59mL/min/1.73m^2)

AMI: Acute Myocardial Infarction, CKD: chronic kidney disease, NCKDA: National Chronic Kidney Disease Audit, MINAP: Myocardial Ischaemia National Audit Project, SCr: serum creatinine

For peer review only

Supplementary table 10: Sensitivity analysis describing the ICD-10 diagnoses (first episode, first diagnostic position) of non-AMI HES cases that are within ±30 days of MINAP only AMI cases from the main analyses

**Main analysis**		
**MINAP only AMI hospitalisations matched with non-AMI HES hospitalisations**		677/766 (88%)
**Short ICD-10 code (1st episode, 1st diagnostic position)**		**n (% of matched)**
I24	Other acute ischaemic heart diseases	114 (17)
I25	Chronic ischaemic heart disease	84 (12)
R07	Pain in throat and chest	63 (9)
J18	Pneumonia, organism unspecified	56 (8)
I20	Angina pectoris	51 (8)
I50	Heart failure	39 (6)
S72	Fracture of femur	18 (3)
N39	Other disorders of urinary system	14 (2)
A41	Other sepsis	11 (2)
R06	Abnormalities of breathing	11 (2)
**Sensitivity analysis – AMI hospitalisations occurring before study start**		
**MINAP only AMI hospitalisations matched with non-AMI HES hospitalisations**		3,908/4,470 (87%)
**Short ICD-10 code (1st episode, 1st diagnostic position)**		**n (% of matched)**
I25	Chronic ischaemic heart disease	996 (25)
I20	Angina pectoris	760 (19)
R07	Pain in throat and chest	588 (15)
I24	Other acute ischaemic heart diseases	271 (7)
I50	Heart failure	129 (3)

For peer review only

J18	Pneumonia, organism unspecified	86 (2)
I48	Atrial fibrillation and flutter	73 (2)
R06	Abnormalities of breathing	49 (1)
I46	Cardiac arrest	42 (1)
S72	Fracture of femur	40 (1)

AMI: Acute Myocardial Infarction, HES: Hospital Episode Statistics, ICD-10: International Classification of Diseases – 10th Edition, MINAP: Myocardial Ischaemia National Audit Project

For peer review only

Supplementary figure 1: Flow diagram illustrating the NCKDA extracts, and resulting contribution of AMI events to this study

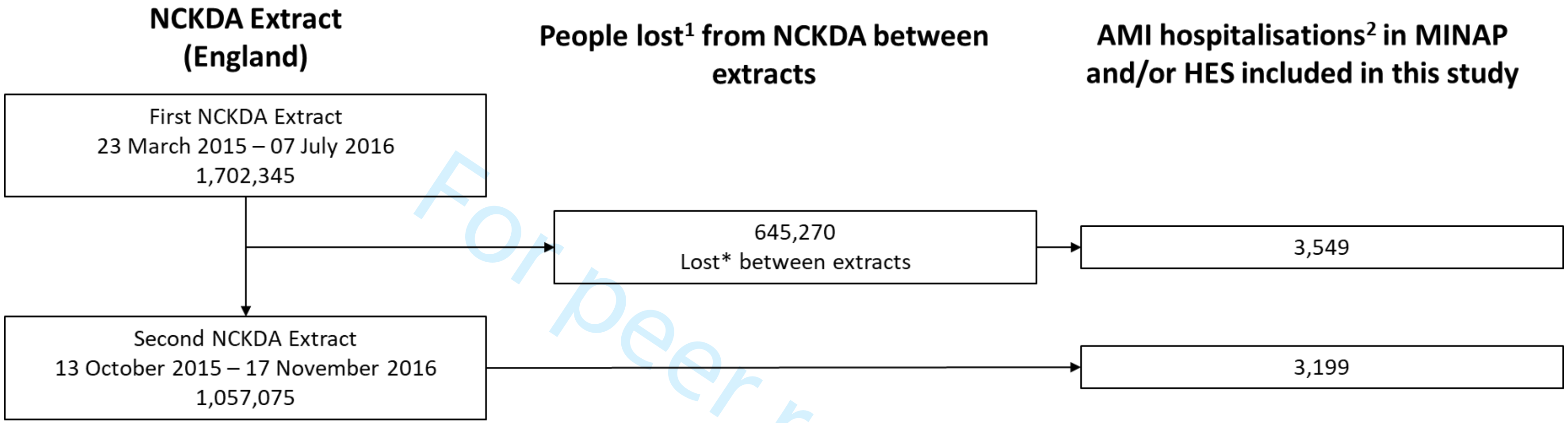

[1]Lost due to death, GP opted out, or person moved GPs

[2]AMI hospitalisations occurring after final NCKDA extract in which each person appears

AMI: acute myocardial infarction, GP: General Practice, NCKDA: National Chronic Kidney Disease Audit, MINAP: Myocardial Ischaemia National Audit Project

For peer review only

Supplementary figure 2: AMI case ascertainment in MINAP and HES datasets over time.

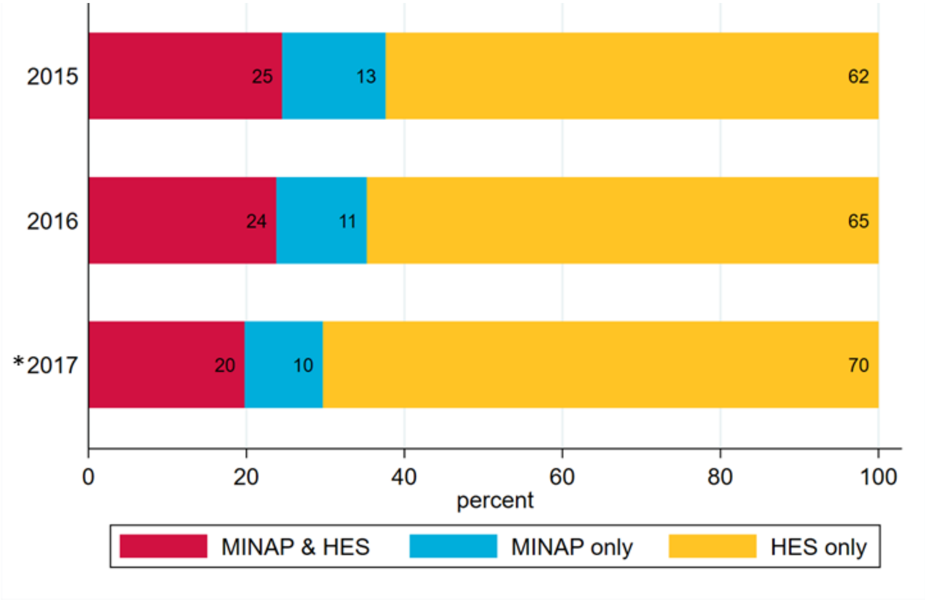

*2017 includes AMI hospitalisations up to 31 March only

AMI: acute myocardial infarction, HES: Hospital Episode Statistics, MINAP: Myocardial Ischaemia National Audit Project

Supplementary figure 3: Bland-Altman plot comparing the mean eGFR (NCKDA eGFR and MINAP eGFR, x-axis) and the difference between the NCKDA eGFR and the MINAP eGFR (y-axis).

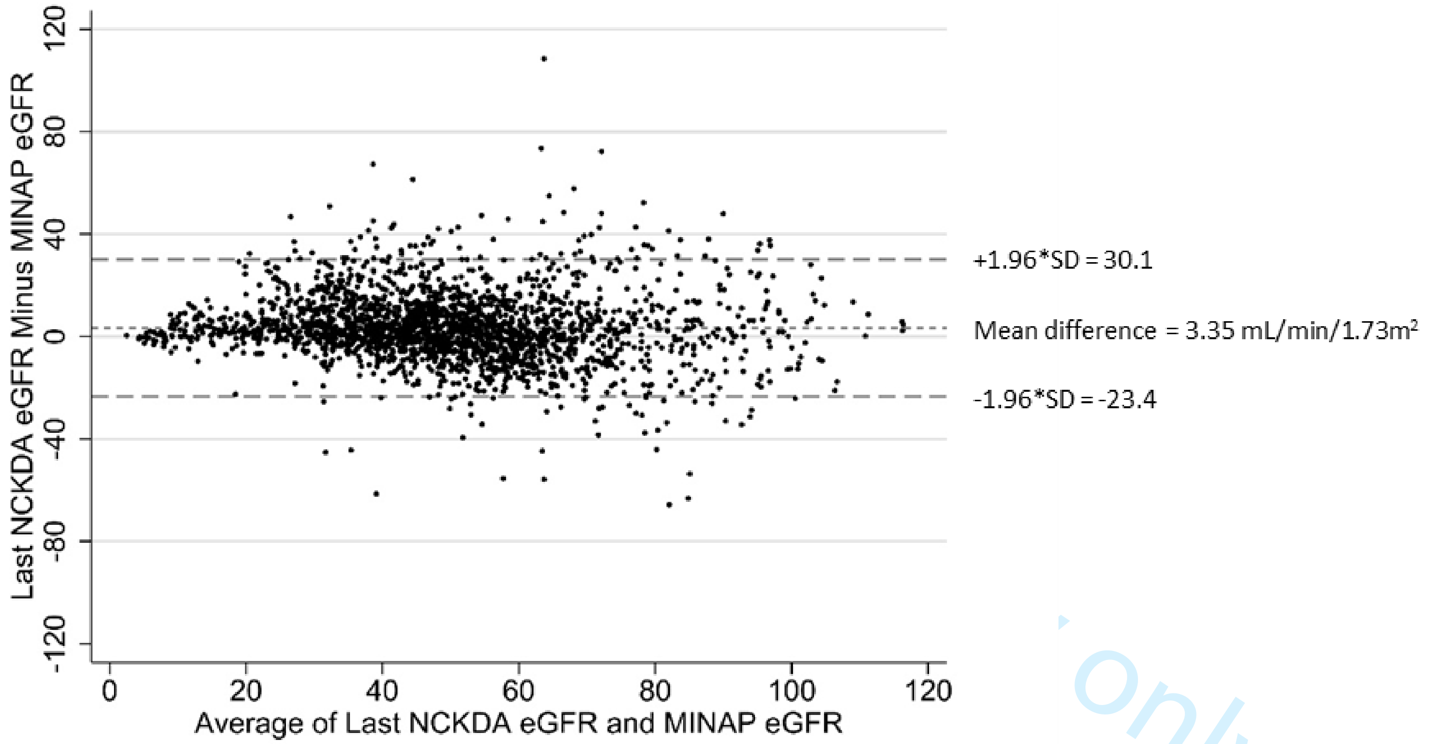

eGFR: estimated glomerular filtration rate, NCKDA: National Chronic Kidney Disease Audit, MINAP: Myocardial Ischaemia National Audit Project

Supplementary figure 4: AMI hospitalisations occurring before study start - comparing AMI case ascertainment overall and by CKD status for AMI hospitalisations in the main analysis and AMI hospitalisations which occurred prior to the last NCKDA extract (sensitivity analysis).

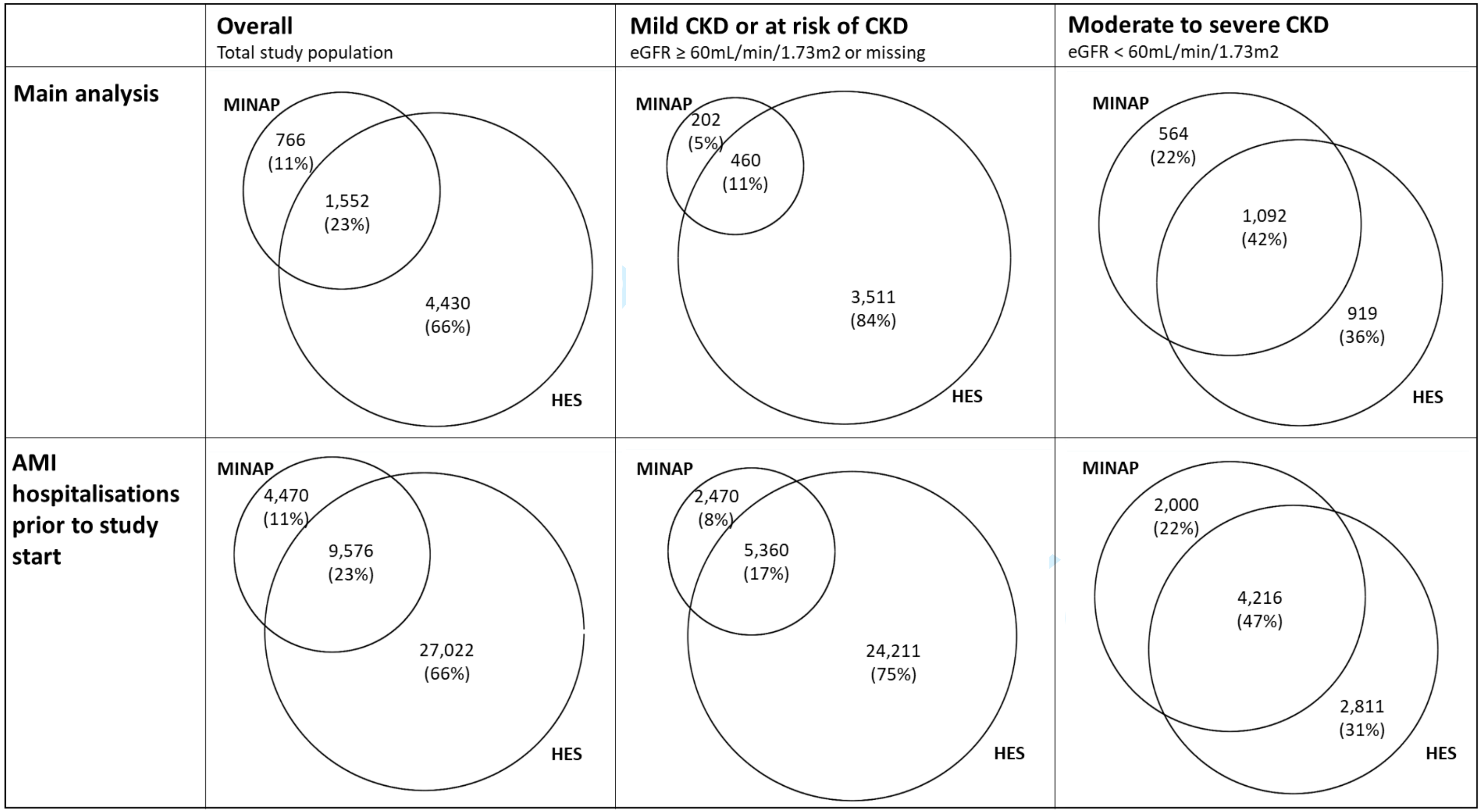

AMI: Acute Myocardial Infarction, CKD: chronic kidney disease, eGFR: estimated glomerular filtration rate, NCKDA: National Chronic Kidney Disease Audit, MINAP: Myocardial Ischaemia National Audit Project

Supplementary figure 5: Sensitivity analyses compared to main analysis, describing AMI recording in MINAP and HES in the overall study population at risk of or with CKD. Areas of Venn diagrams are proportional to the number of cases.

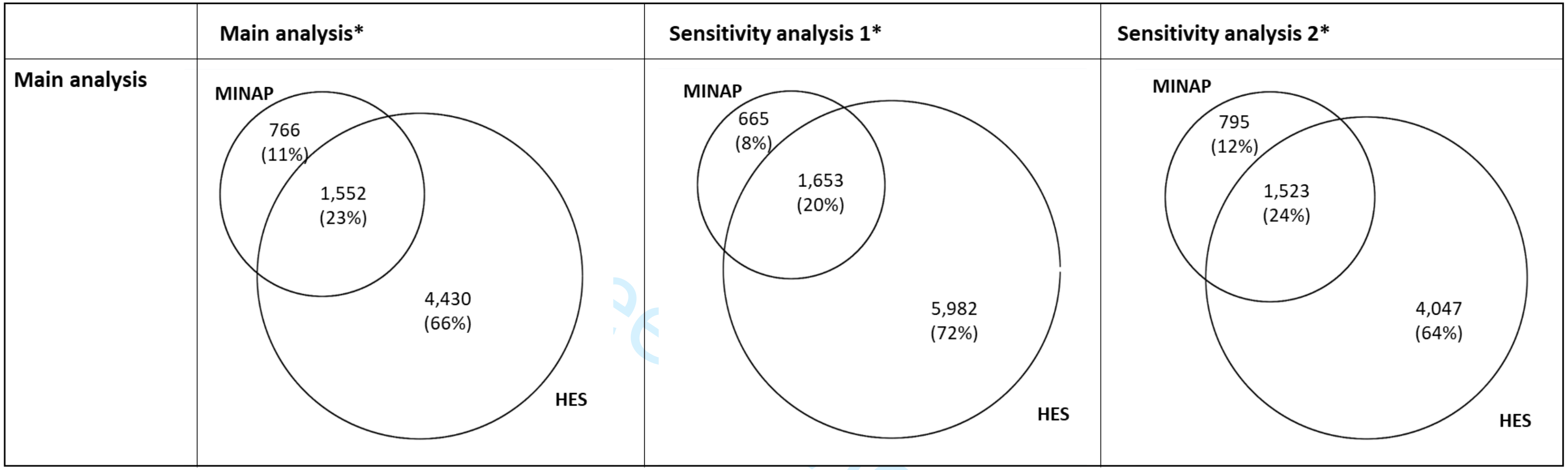

*__Main analysis:__ HES AMI cases defined as AMI recorded in 1st diagnostic position, 1st episode of hospitalisation
*__Sensitivity analysis 1:__ HES AMI cases defined as AMI recorded in 1st or 2nd diagnostic position, 1st episode of hospitalisation
*__Sensitivity analysis 2:__ HES AMI cases in 1st diagnostic position, 1st episode of hospitalisation (as per main analysis) within 30 days of each other counted as a single AMI case, then matched to MINAP AMI cases

AMI: Acute Myocardial Infarction, CKD: chronic kidney disease, HES: Hospital Episode Statistics, MINAP: Myocardial Ischaemia National Audit Project

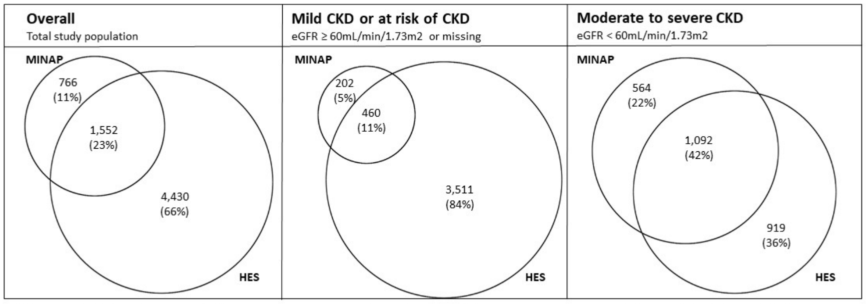

CKD: chronic kidney disease, eGFR: estimated glomerular filtration rate, HES: hospital episode statistics, MINAP: Myocardial Ischaemia National Audit Project

Figure 1: Venn diagrams illustrating AMI recording in MINAP and HES secondary care datasets. Venn diagrams presented overall, and stratified by CKD status (at-risk of or mild CKD, eGFR≥60mL/min/1.73m2 or moderate to severe CKD, eGFR<60 mL/min/1.73m2). Circle areas are proportional to the number of AMI events in each dataset.

338x190mm (96 x 96 DPI)

STROBE 2007 (v4) Statement—Checklist of items that should be included in reports of *cohort studies*

Section/Topic	Item #	Recommendation	Reported on page #
**Title and abstract**	1	(*a*) Indicate the study's design with a commonly used term in the title or the abstract	1
		(*b*) Provide in the abstract an informative and balanced summary of what was done and what was found	4-5
**Introduction**			
Background/rationale	2	Explain the scientific background and rationale for the investigation being reported	7-8
Objectives	3	State specific objectives, including any prespecified hypotheses	8
**Methods**			
Study design	4	Present key elements of study design early in the paper	8-10
Setting	5	Describe the setting, locations, and relevant dates, including periods of recruitment, exposure, follow-up, and data collection	8-9
Participants	6	(*a*) Give the eligibility criteria, and the sources and methods of selection of participants. Describe methods of follow-up	9-10
		(*b*) For matched studies, give matching criteria and number of exposed and unexposed	N/a
Variables	7	Clearly define all outcomes, exposures, predictors, potential confounders, and effect modifiers. Give diagnostic criteria, if applicable	10-13
Data sources/ measurement	8*	For each variable of interest, give sources of data and details of methods of assessment (measurement). Describe comparability of assessment methods if there is more than one group	12-13
Bias	9	Describe any efforts to address potential sources of bias	13-15, 21-22
Study size	10	Explain how the study size was arrived at	8-9
Quantitative variables	11	Explain how quantitative variables were handled in the analyses. If applicable, describe which groupings were chosen and why	12
Statistical methods	12	(*a*) Describe all statistical methods, including those used to control for confounding	13-16
		(*b*) Describe any methods used to examine subgroups and interactions	13-16
		(*c*) Explain how missing data were addressed	15-16
		(*d*) If applicable, explain how loss to follow-up was addressed	14
		(*e*) Describe any sensitivity analyses	15
**Results**			

For peer review only

Participants	13*	(a) Report numbers of individuals at each stage of study—eg numbers potentially eligible, examined for eligibility, confirmed eligible, included in the study, completing follow-up, and analysed	17
		(b) Give reasons for non-participation at each stage	N/a
		(c) Consider use of a flow diagram	Supp figure 1
Descriptive data	14*	(a) Give characteristics of study participants (eg demographic, clinical, social) and information on exposures and potential confounders	17, Table 1
		(b) Indicate number of participants with missing data for each variable of interest	Table 1
		(c) Summarise follow-up time (eg, average and total amount)	18, supp figure 1
Outcome data	15*	Report numbers of outcome events or summary measures over time	17-18
Main results	16	(*a*) Give unadjusted estimates and, if applicable, confounder-adjusted estimates and their precision (eg, 95% confidence interval). Make clear which confounders were adjusted for and why they were included	17-18, Table 2
		(*b*) Report category boundaries when continuous variables were categorized	Table 2
		(*c*) If relevant, consider translating estimates of relative risk into absolute risk for a meaningful time period	N/a
Other analyses	17	Report other analyses done—eg analyses of subgroups and interactions, and sensitivity analyses	18-20
**Discussion**			
Key results	18	Summarise key results with reference to study objectives	20-21
**Limitations**			
Interpretation	20	Give a cautious overall interpretation of results considering objectives, limitations, multiplicity of analyses, results from similar studies, and other relevant evidence	23-24
Generalisability	21	Discuss the generalisability (external validity) of the study results	23-24
**Other information**			
Funding	22	Give the source of funding and the role of the funders for the present study and, if applicable, for the original study on which the present article is based	27

*Give information separately for cases and controls in case-control studies and, if applicable, for exposed and unexposed groups in cohort and cross-sectional studies.

Note: An Explanation and Elaboration article discusses each checklist item and gives methodological background and published examples of transparent reporting. The STROBE checklist is best used in conjunction with this article (freely available on the Web sites of PLoS Medicine at http://www.plosmedicine.org/, Annals of Internal Medicine at http://www.annals.org/, and Epidemiology at http://www.epidem.com/). Information on the STROBE Initiative is available at www.strobe-statement.org.

For peer review only

BMJ Open

The impact of chronic kidney disease on case ascertainment for hospitalised acute myocardial infarction: An English cohort study

Journal:	*BMJ Open*
Manuscript ID	bmjopen-2021-057909.R1
Article Type:	Original research
Date Submitted by the Author:	14-Jan-2022
Complete List of Authors:	Bidulka, Patrick; London School of Hygiene and Tropical Medicine Faculty of Epidemiology and Population Health, Non-Communicable Disease Epidemiology<br>Scott, Jemima; University of Bristol, Population Health Sciences; North Bristol NHS Trust, Richard Bright Renal Service<br>Taylor, Dominic ; University of Bristol, Population Health Sciences; North Bristol NHS Trust<br>Udayaraj, Udaya; Churchill Hospital, Oxford Kidney Unit; University of Oxford, Nuffield Department of Medicine<br>Caskey, Fergus; University of Bristol, Population Health Sciences; North Bristol NHS Trust, Richard Bright Renal Service<br>Teece, Lucy; University of Leicester, Biostatistics Research Group<br>Sweeting, Michael; University of Leicester, Biostatistics Research Group<br>Deanfield, John; Barts Health NHS Trust, National Institute for Cardiovascular Outcomes Research (NICOR); University College London, Institute of Cardiovascular Sciences<br>de Belder, Mark; Barts Health NHS Trust, National Institute for Cardiovascular Outcomes Research (NICOR)<br>Denaxas, S; University College London, Institute of Health Informatics, Faculty of Population Health Sciences; Health Data Research UK<br>Weston, Clive; Glangwili General Hospital<br>Adlam, David; University of Leicester, Department of Cardiovascular Sciences and NIHR Leicester Biomedical Research Centre<br>Nitsch, Dorothea; London School of Hygiene & Tropical Medicine, Department of Non-Communicable Disease Epidemiology
**Primary Subject Heading**:	Renal medicine
Secondary Subject Heading:	Cardiovascular medicine, Epidemiology, General practice / Family practice, Research methods
Keywords:	CARDIOLOGY, Audit < CARDIOLOGY, Myocardial infarction < CARDIOLOGY, NEPHROLOGY, STATISTICS & RESEARCH METHODS

SCHOLARONE™
Manuscripts

I, the Submitting Author has the right to grant and does grant on behalf of all authors of the Work (as defined in the below author licence), an exclusive licence and/or a non-exclusive licence for contributions from authors who are: i) UK Crown employees; ii) where BMJ has agreed a CC-BY licence shall apply, and/or iii) in accordance with the terms applicable for US Federal Government officers or employees acting as part of their official duties; on a worldwide, perpetual, irrevocable, royalty-free basis to BMJ Publishing Group Ltd ("BMJ") its licensees and where the relevant Journal is co-owned by BMJ to the co-owners of the Journal, to publish the Work in this journal and any other BMJ products and to exploit all rights, as set out in our licence.

The Submitting Author accepts and understands that any supply made under these terms is made by BMJ to the Submitting Author unless you are acting as an employee on behalf of your employer or a postgraduate student of an affiliated institution which is paying any applicable article publishing charge ("APC") for Open Access articles. Where the Submitting Author wishes to make the Work available on an Open Access basis (and intends to pay the relevant APC), the terms of reuse of such Open Access shall be governed by a Creative Commons licence – details of these licences and which Creative Commons licence will apply to this Work are set out in our licence referred to above.

Other than as permitted in any relevant BMJ Author's Self Archiving Policies, I confirm this Work has not been accepted for publication elsewhere, is not being considered for publication elsewhere and does not duplicate material already published. I confirm all authors consent to publication of this Work and authorise the granting of this licence.

For peer review only

Title:

The impact of chronic kidney disease on case ascertainment for hospitalised acute myocardial infarction: An English cohort study

Authors:

Patrick Bidulka* (1), Jemima Scott* (2,3), Dominic M Taylor (2,3), Udaya Udayaraj (4), Fergus Caskey (2,3), Lucy Teece (5), Michael Sweeting (5), John Deanfield (6,7), Mark de Belder (6), Spiros Denaxas (8,9), Clive Weston (10), David Adlam* (11), Dorothea Nitsch* (1)

*Equal contribution/joint-authorship

Order	Author	Affiliation	Job title at time of writing	Email
1	Bidulka P*	1	Research Fellow	patrick.bidulka1@lshtm.ac.uk
2	Scott J*	2,3	NIHR Doctoral Research Fellow	jemima.scott@bristol.ac.uk
3	Taylor DM	2,3	Consultant Nephrologist (NBT), Honorary Lecturer (UoB)	dominic.taylor@nbt.nhs.uk
4	Udayarai U	4,5	Consultant Nephrologist Honorary Senior Lecturer	Udaya.Udayaraj@ouh.nhs.uk
5	Caskey FJ	2,3	Consultant senior lecturer	fergus.caskey@bristol.ac.uk
6	Teece L	6	Research Fellow in Medical Statistics	lucy.teece@leicester.ac.uk
7	Sweeting MJ	6	Associate Professor in Biostatistics	michael.sweeting@leicester.ac.uk
8	Deanfield J	7,8	Professor of Cardiology	j.deanfield@ucl.ac.uk
9	de Belder M	7	Professor of Cardiology	mark.debelder@nhs.net
10	Denaxas S	9,10	Professor of Biomedical Informatics	s.denaxas@ucl.ac.uk
11	Weston C	11	Consultant Cardiologist	cliveweston@doctors.org.uk
12	Adlam D	12	Associate Professor in acute and interventional cardiology; Honorary consultant	da134@leicester.ac.uk
13	Nitsch D	1	Professor	dorothea.nitsch@lshtm.ac.uk

For peer review only - http://bmjopen.bmj.com/site/about/guidelines.xhtml

Affiliations:

(1) Department of Non-Communicable Disease Epidemiology, London School of Hygiene & Tropical Medicine, Keppel Street, London UK WC1E 7HT

(2) Population Health Sciences, University of Bristol, Bristol, UK BS8 2PS

(3) Richard Bright Renal Service, North Bristol NHS Trust, Southmead Hospital, Bristol BS10 5NB

(4) Oxford Kidney Unit, Churchill Hospital, Oxford OX3 7LE

(5) Nuffield Department of Medicine, University of Oxford, Oxford, OX3 7BN

(6) Biostatistics Research Group, Department of Health Sciences, University of Leicester, University Road, Leicester, UK, LE1 7RH

(7) National Institute for Cardiovascular Outcomes Research (NICOR), Barts Health NHS Trust, 2nd Floor, 1 St Martin's-Le-Grand, London EC1A 4AS

(8) Institute of Cardiovascular Sciences, University College London. London, UK

(9) Institute of Health Informatics, Faculty of Population Health Sciences, University College London, London UK NW1 2DA

(10) Health Data Research UK, London UK

(11) Glangwili General Hospital, Dolgwili Road, Carmarthen, Wales SA31 2AF

(12) Department of Cardiovascular Sciences, University of Leicester, and NIHR Leicester Biomedical Research Centre, Leicester UK

For peer review only

For peer review only - http://bmjopen.bmj.com/site/about/guidelines.xhtml

Word count (abstract): 300

Word count (main text): 4416

Figures: 1

Tables: 4

Corresponding author: patrick.bidulka1@lshtm.ac.uk

For peer review only

For peer review only - http://bmjopen.bmj.com/site/about/guidelines.xhtml

ABSTRACT (300 words)

Objectives

Acute myocardial infarction (AMI) case ascertainment improves for the UK general population using linked health datasets. Because care pathways for people with chronic kidney disease (CKD) change based on disease severity, AMI case ascertainment for these people may differ. We aimed to determine the association between CKD severity and AMI case ascertainment in two secondary care datasets, and the agreement in estimated glomerular filtration rate (eGFR) between the same datasets.

Methods

We used a cohort design. Primary care records for people with CKD or risk factors for CKD, identified using the National CKD Audit (2015-2017), were linked to the Myocardial Ischaemia National Audit Programme (MINAP, 2007-2017) and Hospital Episode Statistics (HES, 2007-2017) secondary care registries. People with an AMI recorded in either MINAP, HES, or both were included in the study cohort. CKD status was defined using eGFR derived from the most recent serum creatinine value recorded in primary care.

Results

We identified 6,748 AMIs: 23% were recorded in both MINAP and HES, 66% in HES only, and 11% in MINAP only. Compared to people at risk of CKD or with mild CKD, AMIs in people with moderate-severe CKD were more likely to be recorded in both MINAP and HES (42% vs. 11% respectively), or MINAP only (22% vs. 5%), and less likely to be recorded in HES only (36% vs.

For peer review only - http://bmjopen.bmj.com/site/about/guidelines.xhtml

84%). People with AMIs recorded in HES only or MINAP only had increased odds of death during hospitalisation compared to those recorded in both (adjusted odds ratio (OR) 1.61, 95% confidence interval (CI) 1.32-1.96 and OR 1.60, 95% CI 1.26-2.04, respectively). Agreement between eGFR at AMI admission (MINAP) and in primary care was poor (kappa (K) 0.42, standard error (SE) 0.012).

Conclusions

AMI case ascertainment is incomplete in both MINAP and HES, and is associated with CKD severity.

For peer review only

For peer review only - http://bmjopen.bmj.com/site/about/guidelines.xhtml

ARTICLE SUMMARY

- Our study includes a large sample size of 6,748 acute myocardial infarction (AMI) events.
- We have assessed the completeness of AMI hospitalisations recorded in two healthcare datasets widely used in observational research in England.
- We evaluated, for the first time, the validity of using serum creatinine recorded in secondary care at the time of an AMI to estimate pre-AMI CKD stage.
- Generalisability to the general population is limited as the National Chronic Kidney Disease Audit only included people with CKD and/or risk factors for CKD.

For peer review only

INTRODUCTION

Prognosis following acute myocardial infarction (AMI) has improved considerably over the past 50 years such that 85% of individuals now live longer than one year post-AMI.[1] Improved survival is the result of advances in AMI management, driven by evidence from large-scale randomised controlled trials (RCTs).[2-6] Of those admitted to hospital with AMI, 30-40% have chronic kidney disease (CKD):[7] a sustained reduction in kidney function associated with poor outcomes.[8,9] Amongst those with dialysis-dependent CKD only 40% will survive their first year post-AMI.[10] These inferior outcomes may result from higher prevalence of comorbidity,[2] calcific coronary artery disease[3] and the pro-inflammatory effects of uraemia.[4]

Most major RCTs investigating AMI interventions excluded patients with advanced CKD.[11] However, current AMI guidelines from Europe and the United States apply the results of these RCTs to those with or without CKD.[5-7] Clinicians' unease with the dearth of evidence may explain diversion from these AMI guidelines when treating people with CKD.[2,10,11] In the absence of specific RCTs in CKD populations, well-conducted observational analyses can contribute significantly to our understanding and improved management of AMI.

In the UK, data on AMI treatment and outcomes is collected in unlinked, disease-specific registries or in broad registration databases. While there are known differences in the reliability and validity of AMI case ascertainment using these resources in the general population,[12] it is unclear to what extent these differences persist in people with underlying CKD. Multimorbidity and differences in admission and treatment pathways in people with CKD may influence AMI case recording. Reliably identifying which AMI patients have CKD using AMI audit data is also

For peer review only - http://bmjopen.bmj.com/site/about/guidelines.xhtml

difficult; previous studies used admission serum creatinine (SCr) as a proxy for pre-admission CKD stage.[13-15] This unvalidated method risks misclassifying people as having CKD because of the co-incidence of acute kidney injury (AKI) and AMI.[16]

In this study we linked records from the National Chronic Kidney Disease Audit (NCKDA) to the Myocardial Ischaemia National Audit Project (MINAP) and Hospital Episode Statistics (HES) to determine the reliability of these data sources to investigate cardiovascular disease comorbidity and outcomes in people with or at risk of CKD in England. Our objectives were to: (1) Compare case ascertainment of AMI hospitalisations in secondary care datasets (MINAP and HES); (2) determine if MINAP and/or HES case ascertainment defines populations of CKD patients with different risks of death during and after AMI; and (3) compare CKD stage classification using admission serum creatinine recorded in secondary care (MINAP) versus primary care (NCKDA).

METHODS

Data sources

Data from all sources were restricted to patients treated in England. People with or at risk of CKD were identified using primary care data from the NCKDA.[17,18] The NCKDA aimed to optimise the identification and management of people with CKD and/or risk factors for CKD in primary care, and included 10% of English General Practices (GP).[17,18] NCKDA data were collected between 2015 and 2016 in two main cross-sectional data extracts for people with either blood or urine laboratory results indicating CKD and/or risk factors for CKD (prevalent

For peer review only - http://bmjopen.bmj.com/site/about/guidelines.xhtml

hypertension, diabetes mellitus, cardiovascular disease, connective tissue disorders, kidney stones, prostatic disease, family history of kidney disease, previous acute kidney injury (AKI), and users of kidney-damaging medications such as lithium or calcineurin inhibitors).[17] People without an eGFR recorded in primary care were included in the NCKDA only if they were at risk of CKD.

The population identified from NCKDA were linked with Office of National Statistics (ONS) data, January 1998 to September 2019, as well as secondary care data from HES Admitted patient Care (APC) and MINAP, April 2007 to April 2017. HES APC includes hospital admission data for National Health Service (NHS)-treated patients in England, including admission and discharge dates, and diagnoses recorded using International Classification of Diseases 10th Edition (ICD-10) codes.[19] MINAP is an on-going AMI audit in England, Wales, and Northern Ireland which was designed to optimise the care of patients with type one AMI by evaluating the patient pathway from hospital admission with AMI[20] to discharge. MINAP includes admission and discharge dates, treatments, and comorbidities.[21]

Study design

Cohort study.

Study participants

We included people in the NCKDA registered with a GP in England, with one or more AMI hospitalisation recorded in HES or MINAP. People in each NCKDA extract must have been alive according to GP records at the time of that extract. We therefore included people with an AMI

For peer review only - http://bmjopen.bmj.com/site/about/guidelines.xhtml

hospitalisation recorded in MINAP or HES only after the date of their GP's final NCKDA extract. People with an AMI hospitalisation that started prior to the extract date and ended after the extract date were added to the cohort, since they were at risk of death (n=183). In addition, people with an ONS death date indicating death during an AMI hospitalisation that occurred within 90 days prior to the NCKDA extract date were included (n=96), since they were likely misclassified as alive at the time of the extract because of delays in updating the death date in the GP systems. People with an ONS death date earlier than 90 days prior to the extract were excluded (n=4).

Exposures

Our main exposure variable was moderate to severe CKD (eGFR<60mL/min/1.73m^2, CKD stages 3-5), defined using the most recent eGFR recorded in primary care (NCKDA data) prior to the AMI hospitalisation. People with no eGFR recorded in primary care or an eGFR≥60mL/min/1.73m^2 were categorised as at risk of CKD or having mild CKD, respectively. We assumed people with no eGFR recorded in primary care did not have moderate to severe CKD since these people are much less likely to have CKD than those with eGFR recorded.[22] eGFR was calculated using primary care SCr measures and the revised Modification of Diet in Renal Disease (MDRD) equation.[23,24]

Our secondary exposure was CKD stage, defined by the Kidney Disease Improving Global Outcomes CKD staging, based on a single eGFR record without the requirement for two measures three months apart.[25] We combined some CKD stages due to low numbers of AMI

For peer review only - http://bmjopen.bmj.com/site/about/guidelines.xhtml

cases: (1) At risk of CKD & Stages 1-2 (eGFR missing or ≥60mL/min/1.73m^2), (2) Stage 3a (eGFR 45-59mL/min/1.73m^2), (3) Stage 3b (eGFR 30-44mL/min/1.73m^2), and (4) Stages 4-5 (eGFR<30mL/min/1.73m^2).

We used the latest eGFR recorded prior to the AMI hospitalisation to categorise people with a history of kidney transplant into the primary and secondary exposure groups. We categorised people with a history of dialysis prior to the AMI hospitalisation as moderate to severe CKD for the main exposure and CKD stages 4-5 for the secondary exposure, even if the latest eGFR did not agree.

As the use of a single serum creatinine test at the time of AMI hospitalisation to determine CKD stage has not previously been validated, we have used the term “eGFR stage” in place of CKD stage to refer to the eGFR level calculated from this test.

Outcomes

Primary outcome

The primary outcome was AMI case ascertainment, defined as the dataset(s) in which the AMI hospitalisation was recorded. We defined an AMI as being recorded in both HES and MINAP if an AMI hospitalisation in HES was within 30 days of an AMI hospitalisation in MINAP. Where multiple HES AMI hospitalisations fell within 30 days of a MINAP AMI hospitalisation, the HES AMI hospitalisation closest in time to the MINAP AMI admission was selected as the single

For peer review only - http://bmjopen.bmj.com/site/about/guidelines.xhtml

matched event. AMI hospitalisations without a match were categorised as HES or MINAP only. Study participants could contribute multiple AMI hospitalisations.

We defined an AMI in HES data using ICD-10 codes I.21, I.22, or I.23 in the primary admission diagnosis field (first diagnostic position in the first episode of an admission).[26] We categorised AMI subtype (ST-segment elevation myocardial infarction (STEMI) and non-ST-segment elevation myocardial infarction (NSTEMI)) using the UK Biobank coding definitions[27] (**Supplementary table 1**). We defined AMI in MINAP using a previously developed algorithm which uses the discharge diagnosis, electrocardiogram (ECG) results, and the presence of elevated cardiac markers to identify acute coronary syndrome events and subtypes (**Supplementary table 2**). We excluded MINAP hospitalisations classified as unstable angina or other from the analysis.[12]

Secondary outcomes

We investigated in-hospital mortality during each person's first AMI hospitalisation within the study period. In those who survived and were discharged from their first AMI hospitalisation, we also investigated post-discharge mortality using the ONS death date (up to 15 September 2019). Variables in HES, MINAP, and ONS used to define death are described in **Supplementary table 3**. People were considered to have died during AMI hospitalisation if any of these variables indicated in-hospital death, or the ONS death date fell on or between the admission and discharge dates. We used the earliest of the HES or MINAP admission dates and the latest of the HES or MINAP discharge dates to define these dates for AMI hospitalisations recorded in both datasets.

For peer review only - http://bmjopen.bmj.com/site/about/guidelines.xhtml

We investigated the agreement between CKD stage derived from the most recent primary care SCr test (NCKDA data) and eGFR stage derived from the secondary care SCr test conducted within 24 hours of AMI hospitalisation (MINAP data). We used the same methods to determine eGFR stage in MINAP data as we did for NCKDA data.

Covariates

We described age at AMI admission (mean and standard deviation (SD) as well as age category in years: 18-49, 50-64, 65-79, 80+), sex, ethnicity (white or other), index of multiple deprivation quintiles (IMD, as a proxy for socioeconomic status), and relevant comorbidities including angina, cerebrovascular disease, chronic obstruction pulmonary disease (COPD), diabetes mellitus (type 1 and 2), heart failure, hypertension, previous myocardial infarction, and peripheral vascular disease. We also described dialysis and transplant status, and smoking status. Data sources for each key covariate are described in **Supplementary table 4**.

For peer review only

Data analysis

Objective 1 – AMI case ascertainment

We summarised key covariates by CKD status. We used Venn diagrams to describe AMI case ascertainment overall and stratified by CKD status (at risk of CKD or mild CKD vs. moderate to severe CKD). We used multinomial, multivariable logistic regression to quantify the association between CKD stage and AMI case ascertainment. We used the 'HES and MINAP' category as the

For peer review only - http://bmjopen.bmj.com/site/about/guidelines.xhtml

base outcome and reported crude and adjusted relative risk ratios (RRR) and 95% confidence intervals (CI), using 'At risk of CKD & Stages 1-2' as the reference exposure category. We adjusted for sex, age category, ethnicity, IMD quintile, previous AMI, heart failure, COPD, diabetes mellitus, and clustering by participant (using cluster-robust standard errors (SE)). We used a complete case analysis since we could not assume that missing values for ethnicity were missing at random. In a secondary analysis, we stratified these regressions by AMI subtype (STEMI and NSTEMI).

Objective 2 – Risk of death

We used multivariable logistic regression to calculate the odds of death in hospital during each person's first AMI hospitalisation in people with AMI recorded in MINAP only or HES only, relative to MINAP and HES. After confirming the proportional hazards assumption with a Schoenfeld Residuals test on the full multivariable model (p=0.35), we used multivariable Cox regression to estimate hazards ratios (HR) for death during total follow-up in those who survived their first AMI hospitalisation with AMI recorded in MINAP only or HES only, relative to MINAP and HES.

Objective 3 – Agreement between eGFR in primary and secondary care

Finally, to assess the validity of using MINAP-recorded eGFR at AMI admission as a proxy for pre-admission CKD status, we compared eGFR and its corresponding eGFR stage within 24 hours of AMI admission (MINAP data) to the most recent eGFR and its corresponding CKD stage in primary care (NCKDA data). In this analysis, we excluded people with eGFR measures greater than 120mL/min/1.73m^2 in either NCKDA or MINAP as these are unlikely to be true values. We

For peer review only - http://bmjopen.bmj.com/site/about/guidelines.xhtml

drew a Bland-Altman plot to describe differences in the distribution of eGFR measures in primary and secondary care[28] and calculated the percent agreement and kappa agreement statistics between CKD and eGFR stage derived using primary and secondary care eGFRs, respectively. Secondary analyses re-calculated agreements and kappa statistics restricting to people with stages 3a-5 (eGFR<60mL/min/1.73m^2) in primary care and grouped by time between the most recent primary care eGFR measure and the AMI hospitalisation (0-5, 6-11, 12-23, 24-36-month gaps).

Sensitivity analyses

We repeated the main analyses for AMI events occurring prior to the study start date (the latest NCKDA extract). People who experienced AMI hospitalisation before the study start were survivors, since only people alive at the time of the NCKDA were included in the study.

In addition, we re-drew the Venn diagrams after including HES AMI hospitalisations recorded in both the first and second diagnostic positions of the first episode to include AMIs recorded as co-primary diagnoses.[26] We also repeated the matching process between MINAP and HES AMI hospitalisations after combining all HES AMIs within 30 days of each other into a single HES hospitalisation, since it is likely that some AMI events in our dataset have multiple HES hospitalisations recorded if, for example, a patient is transferred between hospitals for treatment. Furthermore, we repeated our multivariable analyses after excluding people with a history of dialysis. Finally, to understand why people may have an AMI hospitalisation recorded in MINAP but not HES, we searched for non-AMI HES hospitalisations within 30 days of the AMI

For peer review only - http://bmjopen.bmj.com/site/about/guidelines.xhtml

recorded in MINAP and described the ICD-10 diagnoses in the first episode of the first diagnostic position.

Missing data

We did a complete case analysis when building our multivariable models. People with missing ethnicity (~1%) and IMD data (<1%) were excluded prior to building our unadjusted, partially adjusted, and adjusted multinomial models.

We used discharge dates to help re-categorise people who were in-hospital at the time of the NCKDA extract into the cohort, as well as to determine death in hospital and the start of follow-up in those who survived their first AMI hospitalisation. Discharge date was missing in 19% and 1% of the MINAP and HES datasets, respectively. We assumed these dates were missing at random and used the median length of admissions in those without missing admission and discharge dates (5 and 4 days in MINAP and HES, respectively) to impute the missing discharge dates.

Patient and public involvement

The Kidney Care UK patient organisation (https://www.kidneycareuk.org/) supported the research questions, grant applications, and the related record linkage application for section 251 permissions critical to the development of the National Chronic Kidney Disease Audit (NCKDA). Patient members of the UK Renal Registry Patient Council

For peer review only - http://bmjopen.bmj.com/site/about/guidelines.xhtml

(https://renal.org/patients/patient-council) reviewed the study results. Their feedback supported a further planned record linkage of renal and cardiac data to look at patient outcomes.

For peer review only

For peer review only - http://bmjopen.bmj.com/site/about/guidelines.xhtml

RESULTS

Study population and baseline characteristics

From 1,702,345 people in England included in the NCKDA, we identified 6,042 (0.4%) people with or at risk of CKD who experienced 6,748 AMIs between the final NCKDA extract and 1 April 2017 (**Supplementary figure 1**). Baseline characteristics stratified by CKD stage are described in **Table 1**. People with moderate to severe CKD accounted for 38% of AMI hospitalisations (2,575). Average age at the time of AMI was 73 years (SD 13). People with moderate to severe CKD were older on average than people with mild CKD or at risk of CKD. Most people were white (92%) and male (61%). The most prevalent comorbidities were hypertension (61%) and diabetes mellitus (35%).

Table 1: Baseline characteristics by CKD stage for all AMI events captured after the study start. n (column %) unless specified otherwise.

**CKD status** (main exposure)	**At risk of CKD or mild CKD**	**Moderate to severe CKD**			**Total**
**CKD stage** (secondary exposure)	**At risk of CKD & 1-2**	**3a**	**3b**	**4-5**	
**Unique individuals**	3,751	1,210	732	349	6,042
**Total number of AMI events, N**	4,173	1,353	825	397	6,748
**Age at AMI event, years, mean (SD)**	70 (13)	79 (10)	82 (9)	79 (12)	73 (13)
**Age category at AMI event, years**					
18-50	299 (7)	20 (1)	1 (0)	10 (3)	330 (5)
50-64	1,163 (28)	91 (7)	44 (5)	38 (10)	1,336 (20)
65-79	1,655 (40)	531 (39)	251 (30)	122 (31)	2,559 (38)
80+	1,056 (25)	711 (53)	529 (64)	227 (57)	2,523 (37)
**Female**	1,430 (34)	638 (47)	416 (50)	168 (42)	2,652 (39)
**Ethnicity**					
White	3,807 (91)	1,263 (93)	773 (94)	361 (91)	6,204 (92)
Other	323 (8)	73 (5)	44 (5)	34 (9)	474 (7)
Missing	43 (1)	17 (1)	8 (1)	2 (1)	70 (1)
**IMD quintile**					
1 (least deprived)	732 (18)	252 (19)	152 (18)	68 (17)	1,204 (18)
2	843 (20)	306 (23)	176 (21)	85 (21)	1,410 (21)

For peer review only - http://bmjopen.bmj.com/site/about/guidelines.xhtml

CKD status (main exposure)	At risk of CKD or mild CKD	Moderate to severe CKD			Total
**CKD stage (secondary exposure)**	**At risk of CKD & 1-2**	**3a**	**3b**	**4-5**	
**Unique individuals**	3,751	1,210	732	349	6,042
**Total number of AMI events, N**	4,173	1,353	825	397	6,748
3	934 (22)	331 (24)	193 (23)	79 (20)	1,537 (23)
4	951 (23)	255 (19)	185 (22)	96 (24)	1,487 (22)
5 (most deprived)	690 (17)	206 (15)	114 (14)	69 (17)	1,079 (16)
Missing	23 (1)	3 (0)	5 (1)	0 (0)	31 (0)
**Dialysis in primary care**					
Peritoneal dialysis	0 (0)	0 (0)	0 (0)	15 (4)	15 (0)
Haemodialysis	0 (0)	0 (0)	0 (0)	24 (6)	24 (0)
Renal dialysis, unspecified	0 (0)	0 (0)	0 (0)	7 (2)	7 (0)
**Kidney transplant**	5 (0)	0 (0)	4 (0)	17 (4)	26 (0)
**Comorbidities**					
Angina	959 (23)	399 (29)	275 (33)	155 (39)	1,788 (26)
Cerebrovascular disease	390 (9)	178 (13)	139 (17)	81 (20)	788 (12)
COPD	514 (12)	209 (15)	168 (20)	58 (15)	949 (14)
Diabetes mellitus	1,293 (31)	465 (34)	356 (43)	233 (59)	2,347 (35)
Heart failure	400 (10)	234 (17)	211 (26)	123 (31)	968 (14)
Hypertension	2,333 (56)	884 (65)	583 (71)	322 (81)	4,122 (61)
Myocardial infarction	1,050 (25)	430 (32)	274 (33)	163 (41)	1,917 (28)
Peripheral vascular disease	229 (5)	108 (8)	74 (9)	47 (12)	458 (7)
**Smoking status**					
non-smoker	1,953 (47)	566 (42)	306 (37)	151 (38)	2,976 (44)
ever-smoker	2,018 (48)	530 (39)	318 (39)	140 (35)	3,006 (45)
Missing	202 (5)	257 (19)	201 (24)	106 (27)	766 (11)

AMI: Acute Myocardial Infarction, CKD: chronic kidney disease, COPD: Chronic Obstructive Pulmonary Disease, HES: Hospital Episode Statistics, IMD: Index of Multiple Deprivation, NCKDA: National Chronic Kidney Disease Audit, MINAP: Myocardial Ischaemia National Audit Project

AMI recording in HES and MINAP

Overall, 23% of AMI hospitalisations were captured by both MINAP and HES datasets (1,552 AMI hospitalisations) (**Figure 1**). There was no substantial change in AMI case ascertainment over time (**Supplementary figure 2**). In people with moderate to severe CKD, 42% of all AMI hospitalisations were captured by both MINAP and HES (1,092 AMI hospitalisations). In people with mild CKD or at risk of CKD, 11% of all AMI hospitalisations were captured by MINAP and HES (460 AMI hospitalisations) (**Figure 1**).

For peer review only - http://bmjopen.bmj.com/site/about/guidelines.xhtml
For peer review only

Relative association between CKD stage and AMI recording

Crude and adjusted relative risk ratios (RRR) and 95% confidence intervals (CI) describing the association between CKD stage and AMI case ascertainment are presented in **Table 2**. After adjusting for key covariates, we observed weak evidence of an increased likelihood of AMI recorded in MINAP only, compared to MINAP and HES, in people with CKD stages 4-5 vs. the at risk of CKD/stages 1-2 group (RRR 1.34, 95% CI 0.97-1.85). Furthermore, compared to the at-risk of CKD/stages 1-2 group, people with CKD stages 3a, 3b, and 4-5 were less likely to have an AMI hospitalisation recorded in HES only vs. MINAP and HES. We did not observe any differences in the likelihood of recording of AMI hospitalisation when stratifying by AMI subtype (**Supplementary table 5**).

Table 2: Multinomial logistic regression comparing the relative risk ratios (RRR) of AMI recording across HES and MINAP according to CKD stage. The comparator outcome is people with AMI recorded in both HES and MINAP databases.

**AMI recording** (outcome, compared to people with AMI recorded in MINAP & HES)	**CKD stage** (exposure)	**Number of AMI admissions, n=**	**Unadjusted[1] RR (95% CI)**	**Partially adjusted[2] RRR (95% CI)**	**Adjusted[3] RRR (95% CI)**
**MINAP only** (N=742)	At risk of CKD/Stages 1-2	196	1	1	1
	Stage 3a	245	1.07 (0.85-1.34)	0.98 (0.77-1.25)	0.98 (0.77-1.25)
	Stage 3b	197	1.17 (0.92-1.49)	1.04 (0.80-1.36)	1.03 (0.79-1.34)
	Stages 4-5	104	1.50 (1.11-2.03)	1.38 (1.01-1.90)	1.34 (0.97-1.85)
**HES only** (N=4,367)	At risk of CKD/Stages 1-2	3,456	1	1	1

For peer review only - http://bmjopen.bmj.com/site/about/guidelines.xhtml

	Stage 3a	557	0.14 (0.12-0.16)	0.14 (0.12-0.17)	0.14 (0.12-0.16)
	Stage 3b	224	0.08 (0.06-0.09)	0.08 (0.07-0.10)	0.08 (0.06-0.10)
	Stages 4-5	130	0.11 (0.08-0.14)	0.11 (0.09-0.15)	0.12 (0.09-0.16)

[1]Complete cases for adjusted model
[2]Adjusted for sex, age at AMI admission, ethnicity (white, other), IMD quintile, clustering by participant
[3]Additionally adjusted for comorbidities previous AMI, heart failure, COPD, diabetes mellitus

AMI: Acute Myocardial Infarction, CKD: chronic kidney disease, COPD: Chronic Obstructive Pulmonary Disease, HES: Hospital Episode Statistics, IMD: Index of Multiple Deprivation, MINAP: Myocardial Ischaemia National Audit Project, RRR: relative risk ratio

For peer review only

Mortality during AMI hospitalisation and post-discharge

Of those with a first AMI recorded in both HES and MINAP, 209 people (15%) died during the AMI hospitalisation, compared to 151 (23%) with a first AMI recorded in MINAP only and 579 (15%) recorded in HES only (**Table 3**). After adjusting for key covariates, people with AMI recorded in MINAP only and HES only had increased odds of in-hospital death compared to people with AMI recorded in both MINAP and HES (odds ratio (OR) 1.60, 95% CI 1.26-2.04 and OR 1.61, 95% CI 1.32-1.96, respectively).

Mean follow-up among people who survived a first AMI hospitalisation was 2.4 years. The rate of death per 100 person-years (PY) during complete follow-up was 18.0 (95% CI 16.4-19.7) for AMI recorded in MINAP and HES, 23.3 (95% CI 20.6-26.5) for AMI recorded in MINAP only, and 10.3 (95% CI 9.61-11.0) for AMI recorded in HES only (**Table 3**). After adjusting for key covariates, there was no evidence of a difference in death during follow-up based on which dataset(s) captured the first AMI hospitalisation.

For peer review only - http://bmjopen.bmj.com/site/about/guidelines.xhtml

Table 3: Death during and after first AMI hospitalisation in total study population at risk of or with CKD.

Death during first AMI hospitalisation (N=5,919)[1]	Number who died, n (%)	-	Unadjusted OR (95% CI)	Adjusted[2] OR (95% CI)
MINAP & HES	209 (15)	-	1	1
MINAP only	151 (23)	-	1.67 (1.32-2.11)	1.60 (1.26-2.04)
HES only	579 (15)	-	0.98 (0.82-1.16)	1.61 (1.32-1.96)
**Death during complete follow-up in those who survive first AMI hospitalisation (N=5,009)[1]**	**Number who died during follow-up, n**	**Rate per 100 person-years (95% CI)**	**Unadjusted HR (95% CI)**	**Adjusted[2] HR (95% CI)**
MINAP & HES	456	18.0 (16.4-19.7)	1	1
MINAP only	237	23.3 (20.6-26.5)	1.27 (1.08-1.48)	1.12 (0.96-1.31)
HES only	847	10.3 (9.61-11.0)	0.59 (0.53-0.67)	1.07 (0.94-1.21)

[1]Complete cases for adjusted model
[2]Adjusted for sex, age at AMI admission, ethnicity (white, other), IMD quintile, previous AMI, heart failure, COPD, diabetes mellitus

AMI: Acute Myocardial Infarction, CKD: chronic kidney disease, COPD: Chronic Obstructive Pulmonary Disease, HES: Hospital Episode Statistics, HR: hazard ratio, IMD: Index of Multiple Deprivation, NCKDA: National Chronic Kidney Disease Audit, MINAP: Myocardial Ischaemia National Audit Project, OR: odds ratio

Agreement between eGFR derived from secondary care vs. primary care data

Of the AMI hospitalisations recorded in MINAP, 2,240 (97%) had SCr recorded within 24 hours of AMI admission (**Supplementary table 6**). Median eGFR at time of admission was 47.6 mL/min/1.73m^2 (IQR 33.5-61.6). The Bland-Altman plot comparing the primary care eGFR and

For peer review only - http://bmjopen.bmj.com/site/about/guidelines.xhtml

the secondary care eGFR indicated a negligible mean difference but wide variation (mean difference 3.35 mL/min/1.73m^2, 95% CI -23.4 to 30.1) (**Supplementary figure 3**).

The percent agreements and kappa statistics between eGFR stage derived from MINAP eGFR at AMI admission and the CKD stage derived from NCKDA using primary care data are shown in **Table 4**. Overall, there was 57.2% agreement in staging (kappa statistic (K) 0.42 (SE 0.012)). When restricting to people with NCKDA-derived CKD stages 3-5, the % agreement and K indicated worse agreement (**Table 4**). However, when looking at agreement in categorising people as having moderate to severe CKD (stages 3-5) vs mild CKD (stages 1-2), agreement improved (82.1% agreement, K 0.55 (SE 0.021)).

When stratifying by months between the primary and secondary care eGFR measures, we observed the best agreement in staging within a 0-5-month gap between the primary and secondary care eGFR measures: 61.0%, K 0.48 (SE 0.03) (**Table 4**). Agreement was worse when the time between eGFR measures increased.

Table 4: Agreement between primary care-derived CKD stage (NCKDA) and secondary care-derived eGFR stage (MINAP)

	**% agreement**	**Kappa statistic (SE)**
**Overall[1]**	57.2%	0.42 (0.012)
**CKD Stages 3a, 3b, 4-5[2]**	53.2%	0.34 (0.015)
**CKD Stages 1-2, 3a-5[3]**	82.1%	0.55 (0.021)
**Overall[1], by time from NCKDA SCr test (primary care) to MINAP SCr test (at AMI secondary care admission)**		
0-5 months	61.0%	0.48 (0.03)
6-11 months	56.7%	0.42 (0.02)
12-23 months	55.9%	0.40 (0.02)
24-36 months	56.8%	0.41 (0.04)

[1]Overall agreement when grouping as (1) Stages 1-2 (eGFR 60-120mL/min/1.73m^2), (2) Stage 3a (eGFR 45-59mL/min/1.73m^2), (3) Stage 3b (eGFR 30-44 mL/min/1.73m^2), (4) Stages 4-5 (eGFR 0-30 mL/min/1.73m^2)

For peer review only - http://bmjopen.bmj.com/site/about/guidelines.xhtml

[2]Agreement when restricting to people with CKD stages 3a-5, grouped as (1) Stage 3a (eGFR 45-59mL/min/1.73m^2), (2) Stage 3b (eGFR 30-44 mL/min/1.73m^2), (3) Stages 4-5 (eGFR 0-30 mL/min/1.73m^2)
[3]Agreement when grouping as (1) Stages 1-2 (eGFR 60-120mL/min/1.73m^2) and (2) Stages 3a-5 (eGFR 0-59mL/min/1.73m^2)

AMI: acute myocardial infarction, CKD: chronic kidney disease, eGFR: estimated glomerular filtration rate, HES: Hospital Episode Statistics, NCKDA: National Chronic Kidney Disease Audit, MINAP: Myocardial Ischaemia National Audit Project, SCr: serum creatinine, SE: standard error

For peer review only

Sensitivity analyses

AMI case ascertainment in MINAP and HES was similar in AMI hospitalisations recorded prior to the study start (sensitivity analysis) compared to after the study start (main analysis) (**Supplementary tables 7-8, Supplementary figure 4**). There were also no major differences in agreement between CKD staging derived in primary vs. secondary care when investigating AMI hospitalisations prior to the study start (**Supplementary table 9**).

After expanding the AMI definition in HES to include any hospitalisations with AMI coded in the second diagnostic position as well as the first, the proportion of AMI hospitalisations captured in both HES and MINAP decreased slightly (**Supplementary figure 5**). After combining HES AMI admissions within 30 days of each other for the same person, we observed a 1% increase in the proportion of AMI hospitalisations recorded in both MINAP and HES (**Supplementary figure 5**). Results were also similar when excluding people with a history of dialysis (**Supplementary tables 10-11**).

Finally, the ten most common diagnoses in HES matching with the MINAP only AMI hospitalisations from the main analysis are shown in **Supplementary table 12**. Eighty-eight

For peer review only - http://bmjopen.bmj.com/site/about/guidelines.xhtml

percent of unmatched MINAP AMIs had a non-AMI HES hospitalisation within 30 days. These were mainly CVD or respiratory infection-related ICD-10 diagnoses.

DISCUSSION

We compared recording of AMI hospitalisations for people with CKD between two large secondary healthcare datasets in England. In a cohort of 6,042 people, we found that both HES and MINAP missed a significant proportion of AMI hospitalisations. CKD stage influenced likelihood of AMI recording by dataset: AMI hospitalisations in people with moderate to severe CKD were more likely to be recorded in MINAP compared to people at risk of CKD or with mild CKD. We found an association between AMI hospitalisation recording by dataset and in-hospital mortality. There was marked variation between eGFR at AMI admission and preceding eGFR measurements in primary care, but no obvious systematic bias in terms of over/underestimation of eGFR at AMI admission.

Our results agree with previous research demonstrating incomplete capture of AMI events by individual healthcare datasets in the overall English population and extend them to a population with CKD. Herrett et al[12] showed 46% agreement when restricting to MINAP and HES recorded AMI hospitalisations, which is close to the 42% agreement we found in people with moderate to severe CKD. A smaller single-centre study by Torabi et al[29] found 32% agreement between MINAP and the hospital information department (responsible for HES coding).

For peer review only - http://bmjopen.bmj.com/site/about/guidelines.xhtml

In contrast to both studies, we found significantly worse agreement in case ascertainment for AMI hospitalisations between MINAP and HES for people at risk of CKD or with mild CKD. Torabi et al[29] collected data on renal function but found likelihood of AMI case recording in MINAP to reduce with advancing CKD stage. Differences in results between this and our study could be ascribed to changes in management of patients and/or event recording over time, differences in the populations studied and local practice in the single centre analysed by Torabi et al.[29]

The high prevalence of CKD risk factors in people at risk of or with mild CKD could put them at greater risk of type 2 AMI; a mismatch of myocardial oxygen supply and demand in the absence of the “classical” coronary artery plaque rupture with thrombosis reflective of type 1 AMI.[20] People with type 2 AMI are typically older, with a greater burden of comorbidities than those with type 1 AMI, and have poor outcomes.[30] HES is likely to include more type 2 AMI than MINAP as clinical coders for the latter are asked to select type 1 AMI only.

People with AMI recorded in both MINAP and HES had lower in-hospital mortality compared with those with AMI recorded in either MINAP or HES only. Our findings agree with Herrett et al;[12] patients with AMI recorded in only one source had a higher mortality than those with events recorded in more than one source. Higher in-hospital mortality in the MINAP only cases is likely to reflect the referral of severe and complex AMI cases to cardiology, including a higher STEMI to NSTEMI ratio.

Across all levels of eGFR, we found significant variation between eGFR stage derived from SCr taken within 24 hours of AMI admission (recorded in MINAP) and that derived from SCr in primary care, which is in line with reported variability of eGFR in validation studies.[23,24] As

For peer review only - http://bmjopen.bmj.com/site/about/guidelines.xhtml

expected with known limitations of using MDRD eGFR to estimate kidney function for GFRs above 60mL/min/1.73m^2, binary classification between individuals with CKD stages 3-5 and those with stages 1-2 is more reliable than classification by CKD stage. These findings suggest that although previous research[13-15] using SCr at AMI admission recorded in MINAP as a proxy for baseline CKD stage may result in misclassification, it is unlikely to have resulted in a systematic bias in either over- or under-estimation of CKD stage. Differences between SCr recorded in primary care and SCr recorded in MINAP may reflect progression of CKD, differential use of medication that affects the renin-angiotension-aldosterone system, AKI at the time of serum sampling (although changes in serum creatinine are unlikely to show within 24 hours of AMI onset), or variation around the mean.

Limitations

The NCKDA only included people with CKD and/or risk factors for CKD; therefore, we cannot generalise our results to people without risk factors for CKD. We may have incorrectly misclassified people who have no documented tests for CKD in primary care as having risk factors for CKD only; however, previous work has shown this group of people are much less likely to have CKD than those who do have CKD tests recorded in primary care.[22] Furthermore, we included people with at least one reduced kidney function test as potentially having CKD since not every patient undergoes regular CKD testing in our routine clinical datasets. Defining CKD using one eGFR measurement will have led to some misclassification. However, as people with CKD have very high cardiovascular risk and because of the infrequent serum creatinine measurement in primary care, applying the chronicity criterion would have led to a selected cohort of people who did not develop a myocardial infarction until the second measurement

For peer review only - http://bmjopen.bmj.com/site/about/guidelines.xhtml

had been done. Our results are likely impacted by residual confounding, since we were limited in the number of relevant comorbidities we could include in our multivariable models. Finally, AMI misclassification in HES data may have occurred due to the structure and level of detail available in this dataset. For example, we may have missed AMI cases by including only those recorded in the first diagnostic position of the first episode of a HES admission; however, our sensitivity analysis which included AMI hospitalisations recorded in the first or second diagnostic position showed similar results. In addition, unlike MINAP data, HES data do not include ECG results and troponin levels, which we could have used to reduce potential misclassification. Inclusion of the first diagnostic position of later episodes was undertaken in a similar study investigating AMI case ascertainment in people with malignancy, with little improvement in agreement between datasets.[31]

Future research

This study demonstrates how AMI case ascertainment in England can be improved by using linked healthcare datasets. Further research investigating cardiovascular and kidney disease incidence, prevalence and outcomes should follow this approach. Other countries with similarly rich, yet fragmented healthcare datasets would benefit from applying similar methods to evaluate the validity and completeness of cardiovascular and kidney disease capture in similar data. Optimising data quality in healthcare datasets and simplifying the process of data linkage would facilitate high-quality observational research to inform the design of future RCTs and provide estimated treatment effects where RCT data are lacking.

Conclusion

For peer review only

For peer review only - http://bmjopen.bmj.com/site/about/guidelines.xhtml

The use of linked healthcare datasets should be prioritised in observational research investigating multimorbidity.

For peer review only

For peer review only - http://bmjopen.bmj.com/site/about/guidelines.xhtml

ACKNOWLEDGEMENTS

MINAP is commissioned, as part of the ‘National Cardiac Audit Project’ by the Healthcare Quality Improvement Partnership. This work uses data that has been provided by patients and collected by the NHS as part of their care and support.

We acknowledge and thank UK cardiovascular, renal, and primary care physicians as well as hospital and community audit and coding teams whose diligent data collection has provided the data for these analyses. In particular, we would like to thank James Chal, Anil Gunesh, Andrew Harrison, and Akosua Donkor from NICOR; and Kathryn Griffith, Matthew Harker, Yvonne Silove, Tasneem Hoosain, Nick Wilson, Ronnie Moodley, Richard Fluck, Chris Gush, David Wheeler, Liam Smeeth, Ron Cullen, Andy Syme, Richard Gunn, Paul Wright, Hugh Gallagher, Sion Edwards, Fiona Loud, Nick Palmer, Richard Fluck, Anita Sharma, Kate Cheema, Andy Syme, Sally Hull, Ben Caplin, Lois Kim, and Faye Cleary from the NCKDA steering, clinical reference, and statistical groups.

AUTHOR CONTRIBUTIONS

JS, DMT, DN, DA, FC, and UU conceived the study. JS, DMT, DN, DA, FC, UU, and PB designed the study. PB and DN had access to and analysed the data. PB and JS drafted the manuscript. PB, JS, DMT, UU, FC, LT, MS, JD, MB, SD, CW, DA, DN (all authors) critically appraised the results and edited the manuscript. All authors approved the final version of the manuscript. The lead

For peer review only - http://bmjopen.bmj.com/site/about/guidelines.xhtml

authors confirm all authors meet ICJME authorship criteria, and no one who meets ICJME criteria have been excluded.

COMPETING INTERESTS

All authors have completed an ICJME form. PB, JS, DMT, FC, LT, MDB, and SD have nothing to declare. UU declares a grant from the Health Foundation to undertake quality improvement unrelated to this work. MS declares funding from Cancer Research UK (C53325/A21134) and British Heart Foundation (SP/16/5/32415) for research activities related to this work looking at acute myocardial infarction ascertainment in a cancer population. JS declares Doctoral research funding from the NIHR for related research looking at acute myocardial infarction care for people with chronic kidney disease. JD declares grants from British Heart Foundation paid to his institution unrelated to this work. JD also declares consulting fees from Novo Nordisk, and honoraria from Amgen, Boehringer Ingelheim, Merck, Pfizer, Aegerion, Novartis, Sanofi, Takeda, Novo Nordisk, and Bayer, unrelated to this work. JD is also member of a study steering committee with Novo Nordisk. CW declares he is clinical lead of the Myocardial Ischaemia National Audit Project. DA reports research funding and in-kind support from Astra Zeneca inc. for unrelated research and educational funding from Abbott Vascular inc. to support a clinical research fellow doing unrelated research. DA has conducted consultancy for General Electric inc. to support general research funds. DN is the UK Kidney Association Director of Informatics Research. DN is also on the steering group for two GlaxoSmithKline funded studies that investigate kidney function in children and adults in Sub-Saharan Africa.

For peer review only - http://bmjopen.bmj.com/site/about/guidelines.xhtml

FUNDING

This work was supported by Kidney Research UK (grant number IN_008_20180304) and the Health Foundation (grant number 1725841). JS is a Doctoral Research Fellow funded by the NIHR (NIHR 300906). DA is funded by a joint research grant from the British Heart Foundation (SP/16/5/32415) and Cancer Research UK (C53325/A21134).

DATA SHARING

Due to data sharing agreements, we are not able to share these data. Researchers interested in using these data should consult the websites for the NCKDA (https://www.lshtm.ac.uk/research/centres-projects-groups/ckdaudit#welcome) and MINAP (https://www.nicor.org.uk/national-cardiac-audit-programme/myocardial-ischaemia-minap-heart-attack-audit/).

ETHICS APPROVAL

This study was approved by the London School of Hygiene & Tropical Medicine LEO ethics committee (ID 16988) and the National Chronic Kidney Disease Audit (NCKDA) Steering Committee. Audits used in this study are covered by Section 251 approvals (NHS Act 2006)

For peer review only - http://bmjopen.bmj.com/site/about/guidelines.xhtml

which allows data to be collected without individual patient consent for medical research when it is not possible to use anonymised information and when seeking consent is not practical.

REFERENCES

1. Foundation BH. Heart statistics. Accessed 28/02/2020, 2020. https://www.bhf.org.uk/what-we-do/our-research/heart-statistics
2. Pitt B, Remme W, Zannad F, et al. Eplerenone, a selective aldosterone blocker, in patients with left ventricular dysfunction after myocardial infarction. *The New England journal of medicine*. Apr 3 2003;348(14):1309-21. doi:10.1056/NEJMoa030207
3. Januzzi JL, Cannon CP, DiBattiste PM, Murphy S, Weintraub W, Braunwald E. Effects of renal insufficiency on early invasive management in patients with acute coronary syndromes (The TACTICS-TIMI 18 Trial). *The American journal of cardiology*. Dec 1 2002;90(11):1246-9.
4. Bhatt DL, Roe MT, Peterson ED, et al. Utilization of early invasive management strategies for high-risk patients with non-ST-segment elevation acute coronary syndromes: results from the CRUSADE Quality Improvement Initiative. *Jama*. Nov 3 2004;292(17):2096-104. doi:10.1001/jama.292.17.2096
5. Kedhi E, Fabris E, van der Ent M, et al. Six months versus 12 months dual antiplatelet therapy after drug-eluting stent implantation in ST-elevation myocardial infarction (DAPT-STEMI): randomised, multicentre, non-inferiority trial. *BMJ (Clinical research ed)*. Oct 2 2018;363:k3793. doi:10.1136/bmj.k3793
6. Serruys PW, Kogame N, Katagiri Y, et al. Clinical outcomes of state-of-the-art percutaneous coronary revascularisation in patients with three-vessel disease: two-year follow-up of the SYNTAX II study. *EuroIntervention*. Jun 12 2019;15(3):e244-e252. doi:10.4244/eij-d-18-00980
7. Wright RS, Reeder GS, Herzog CA, et al. Acute myocardial infarction and renal dysfunction: a high-risk combination. *Ann Intern Med*. Oct 1 2002;137(7):563-70.
8. Santopinto JJ, Fox KA, Goldberg RJ, et al. Creatinine clearance and adverse hospital outcomes in patients with acute coronary syndromes: findings from the global registry of acute coronary events (GRACE). *Heart*. Sep 2003;89(9):1003-8. doi:10.1136/heart.89.9.1003
9. Fox CS, Muntner P, Chen AY, et al. Use of evidence-based therapies in short-term outcomes of ST-segment elevation myocardial infarction and non-ST-segment elevation myocardial infarction in patients with chronic kidney disease: a report from the National Cardiovascular Data Acute Coronary Treatment and Intervention Outcomes Network registry. *Circulation*. Jan 26 2010;121(3):357-65. doi:10.1161/circulationaha.109.865352
10. Herzog CA, Ma JZ, Collins AJ. Poor long-term survival after acute myocardial infarction among patients on long-term dialysis. *The New England journal of medicine*. Sep 17 1998;339(12):799-805. doi:10.1056/nejm199809173391203

For peer review only - http://bmjopen.bmj.com/site/about/guidelines.xhtml

11. Konstantinidis I, Nadkarni GN, Yacoub R, et al. Representation of Patients With Kidney Disease in Trials of Cardiovascular Interventions: An Updated Systematic Review. *JAMA internal medicine*. Jan 2016;176(1):121-4. doi:10.1001/jamainternmed.2015.6102
12. Herrett E, Shah AD, Boggon R, et al. Completeness and diagnostic validity of recording acute myocardial infarction events in primary care, hospital care, disease registry, and national mortality records: cohort study. *BMJ (Clinical research ed)*. May 20 2013;346:f2350. doi:10.1136/bmj.f2350
13. Gupta T, Paul N, Kolte D, et al. Association of chronic renal insufficiency with in-hospital outcomes after percutaneous coronary intervention. *J Am Heart Assoc*. Jun 16 2015;4(6):e002069. doi:10.1161/jaha.115.002069
14. Rozenbaum Z, Benchetrit S, Minha S, et al. The Effect of Admission Renal Function on the Treatment and Outcome of Patients with Acute Coronary Syndrome. *Cardiorenal Med*. Jun 2017;7(3):169-178. doi:10.1159/000455239
15. Shaw C, Nitsch D, Steenkamp R, et al. Inpatient coronary angiography and revascularisation following non-ST-elevation acute coronary syndrome in patients with renal impairment: a cohort study using the Myocardial Ischaemia National Audit Project. *PLoS One*. 2014;9(6):e99925. doi:10.1371/journal.pone.0099925
16. Grams ME, Astor BC, Bash LD, Matsushita K, Wang Y, Coresh J. Albuminuria and estimated glomerular filtration rate independently associate with acute kidney injury. *J Am Soc Nephrol*. Oct 2010;21(10):1757-64. doi:10.1681/asn.2010010128
17. Nitsch D CB HS, Wheeler D. National Chronic Kidney Disease Audit - National Report Part 1. 2017;
18. Mudie K CF CB, Wheeler D, Hull S, Nitsch D. National Chronic Kidney Disease Audit: National Report Part 2. 2017;
19. Hospital Episode Statistics. 2020. https://digital.nhs.uk/data-and-information/data-tools-and-services/data-services/hospital-episode-statistics
20. Thygesen K, Alpert JS, Jaffe AS, et al. Fourth universal definition of myocardial infarction (2018). *Eur Heart J*. Aug 25 2018;doi:10.1093/eurheartj/ehy462
21. audit) MIMHA. https://www.nicor.org.uk/national-cardiac-audit-programme/myocardial-ischaemia-minap-heart-attack-audit/,
22. Iwagami M, Tomlinson LA, Mansfield KE, et al. Validity of estimated prevalence of decreased kidney function and renal replacement therapy from primary care electronic health records compared with national survey and registry data in the United Kingdom. *Nephrol Dial Transplant*. Apr 1 2017;32(suppl_2):ii142-ii150. doi:10.1093/ndt/gfw318
23. Levey AS, Coresh J, Greene T, et al. Using standardized serum creatinine values in the modification of diet in renal disease study equation for estimating glomerular filtration rate. *Ann Intern Med*. Aug 15 2006;145(4):247-54. doi:10.7326/0003-4819-145-4-200608150-00004
24. Levey AS, Stevens LA, Schmid CH, et al. A new equation to estimate glomerular filtration rate. *Ann Intern Med*. May 5 2009;150(9):604-12. doi:10.7326/0003-4819-150-9-200905050-00006
25. Stevens PE, Levin A. Evaluation and management of chronic kidney disease: synopsis of the kidney disease: improving global outcomes 2012 clinical practice guideline. *Ann Intern Med*. Jun 4 2013;158(11):825-30. doi:10.7326/0003-4819-158-11-201306040-00007

For peer review only - http://bmjopen.bmj.com/site/about/guidelines.xhtml

26. Herbert A, Wijlaars L, Zylbersztejn A, Cromwell D, Hardelid P. Data Resource Profile: Hospital Episode Statistics Admitted Patient Care (HES APC). *Int J Epidemiol*. Aug 1 2017;46(4):1093-1093i. doi:10.1093/ije/dyx015

27. Schnier C BK NJ, Sudlow C. Definitions of Acute Myocardial Infarction and Main Myocardial Infarction Pathological Types - UK Biobank Phase 1 Outcomes Adjudication. Accessed 12 January, 2021. https://biobank.ndph.ox.ac.uk/showcase/showcase/docs/alg_outcome_mi.pdf.

28. Giavarina D. Understanding Bland Altman analysis. *Biochem Med (Zagreb)*. 2015;25(2):141-51. doi:10.11613/bm.2015.015

29. Torabi A, Cleland JG, Sherwi N, et al. Influence of case definition on incidence and outcome of acute coronary syndromes. *Open Heart*. 2016;3(2):e000487. doi:10.1136/openhrt-2016-000487

30. Baron T, Hambraeus K, Sundström J, Erlinge D, Jernberg T, Lindahl B. Type 2 myocardial infarction in clinical practice. *Heart*. Jan 2015;101(2):101-6. doi:10.1136/heartjnl-2014-306093

31. Coles B, Teece L, Weston C, et al. Case-ascertainment of acute myocardial infarction hospitalizations in cancer patients: a cohort study using English linked electronic health data. *European Heart Journal - Quality of Care and Clinical Outcomes*. 2021;doi:10.1093/ehjqcco/qcab045

For peer review only

For peer review only - http://bmjopen.bmj.com/site/about/guidelines.xhtml

FIGURE LEGEND

Figure 1: Venn diagrams illustrating AMI recording in MINAP and HES secondary care datasets. Venn diagrams presented overall, and stratified by CKD status (at-risk of or mild CKD, eGFR≥60mL/min/1.73m^2 or moderate to severe CKD, eGFR<60 mL/min/1.73m^2). Circle areas are proportional to the number of AMI events in each dataset.

TABLE LEGEND

Table 1: Baseline characteristics by CKD stage for all AMI events captured after the study start. n (column %) unless specified otherwise.

Table 2: Multinomial logistic regression comparing the relative risk ratios (RRR) of AMI recording across HES and MINAP according to CKD stage. The comparator outcome is people with AMI recorded in both HES and MINAP databases.

Table 3: Death during and after first AMI hospitalisation in total study population at risk of or with CKD.

Table 4: Agreement between primary care-derived CKD stage (NCKDA) and secondary care-derived eGFR stage (MINAP)

For peer review only - http://bmjopen.bmj.com/site/about/guidelines.xhtml

ALTERNATIVE ABSTRACT (392 words)

Objectives

Acute myocardial infarction (AMI) case ascertainment improves for the UK general population using linked health datasets. Because care pathways for people with chronic kidney disease (CKD) change based on disease severity, AMI case ascertainment for these people may differ compared to the general population. We aimed to determine the association between CKD severity and AMI case ascertainment in two secondary care datasets, and the agreement in estimated glomerular filtration rate (eGFR) between the same datasets.

Methods

We used a cohort study design. Primary care records for people with CKD or risk factors for CKD, identified using the National CKD Audit (2015-2017), were linked to the Myocardial Ischaemia National Audit Programme (MINAP, 2007-2017) and Hospital Episode Statistics (HES, 2007-2017) secondary care registries. People with an AMI recorded in either MINAP, HES, or both were included in the study cohort. CKD status was defined using estimated glomerular filtration rate (eGFR), derived from the most recent serum creatinine value recorded in primary care. Moderate-severe CKD was defined as eGFR<60mL/min/1.73m^2, and mild CKD or at risk of CKD was defined as eGFR≥60mL/min/1.73m^2 or eGFR missing. CKD stages were grouped as follows: (1) At risk of CKD & Stages 1-2 (eGFR missing or ≥60mL/min/1.73m^2), (2) Stage 3a (eGFR 45-59mL/min/1.73m^2), (3) Stage 3b (eGFR 30-44mL/min/1.73m^2), and (4) Stages 4-5 (eGFR<30mL/min/1.73m^2).

For peer review only - http://bmjopen.bmj.com/site/about/guidelines.xhtml

Results

We identified 6,748 AMIs. Of these events, 23% were recorded in both MINAP and HES, 66% in HES only and 11% in MINAP only. Compared to people at risk of CKD or with mild CKD, AMIs in people with moderate-severe CKD were more likely to be recorded in both MINAP and HES (42% vs. 11% respectively), or MINAP only (22% vs. 5%), and less likely to be recorded in HES only (36% vs. 84%). People with AMIs recorded in HES only or MINAP only had increased odds of death during hospitalisation compared to those recorded in both (adjusted odds ratio (OR) 1.61, 95% confidence interval (CI) 1.32-1.96 and OR 1.60, 95% CI 1.26-2.04, respectively). Agreement between eGFR at AMI admission (MINAP) and in primary care was poor (kappa (K) 0.42, standard error (SE) 0.012).

Conclusions

AMI case ascertainment is incomplete in both MINAP and HES, and is associated with CKD severity. Future observational research in England studying the interaction between cardiovascular and kidney disease should use linked health data to study disease incidence, prevalence, and treatments.

For peer review only

For peer review only - http://bmjopen.bmj.com/site/about/guidelines.xhtml

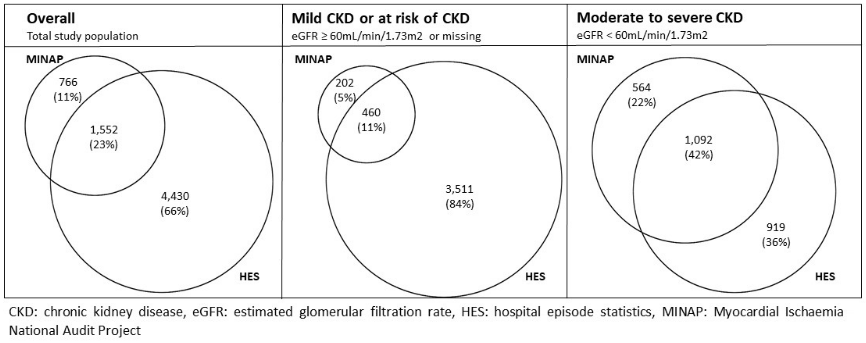

Figure 1: Venn diagrams illustrating AMI recording in MINAP and HES secondary care datasets. Venn diagrams presented overall, and stratified by CKD status (at-risk of or mild CKD, eGFR≥60mL/min/1.73m2 or moderate to severe CKD, eGFR<60 mL/min/1.73m2). Circle areas are proportional to the number of AMI events in each dataset.

338x190mm (96 x 96 DPI)

Supplementary materials

Supplementary tables

Supplementary table 1: ICD-10 codes for AMI identified in HES

Supplementary table 2: CALIBER definition of AMI subtypes (STEMI, NSTEMI) using MINAP data.

Supplementary table 3: Variables used to define death in-hospital and post-AMI discharge.

Supplementary table 4: Details on data sources for covariates.

Supplementary table 5: AMI database capture by CKD status (binary) stratified by AMI subtype (STEMI or NSTEMI)

Supplementary table 6: Serum creatinine (SCr) measured in the first 24 hours of AMI hospitalization recorded in MINAP, stratified by CKD stage defined in primary care (NCKDA)

Supplementary table 7: AMI hospitalisations occurring before study start – description of people in the NCKDA who experienced AMI hospitalisation recorded in MINAP or HES before the study start. n (column %) unless specified otherwise.

Supplementary table 8: AMI hospitalisations occurring before study start - multinomial logistic regression comparing the relative risk ratios (RRR) of AMI recording across HES and MINAP according to CKD stage. The comparator outcome is people with AMI recorded in both HES and MINAP databases.

Supplementary table 9: AMI hospitalisations occurring before study start - agreement between primary care-derived CKD stage (NCKDA) and secondary care-derived CKD stage (MINAP)

Supplementary table 10: Multinomial logistic regression comparing the relative risk ratios (RRR) of AMI recording across HES and MINAP according to CKD stage, after excluding those with a history of dialysis. The comparator outcome is people with AMI recorded in both HES and MINAP databases.

Supplementary table 11: Death during and after first AMI hospitalisation in study population at risk of or with CKD, excluding those with a history of dialysis.

Supplementary table 12: Sensitivity analysis describing the ICD-10 diagnoses (first episode, first diagnostic position) of non-AMI HES cases that are within ±30 days of MINAP only AMI cases from the main analyses

Supplementary figures

Supplementary figure 1: Flow diagram illustrating the NCKDA extracts, and resulting contribution of AMI events to this study

Supplementary figure 2: AMI case ascertainment in MINAP and HES datasets over time.

Supplementary figure 3: Bland-Altman plot comparing the mean eGFR (NCKDA eGFR and MINAP eGFR, x-axis) and the difference between the NCKDA eGFR and the MINAP eGFR (y-axis).

Supplementary figure 4: AMI hospitalisations occurring before study start - comparing AMI case ascertainment overall and by CKD status for AMI hospitalisations in the main analysis and AMI hospitalisations which occurred prior to the last NCKDA extract (sensitivity analysis).

Supplementary figure 5: Sensitivity analyses compared to main analysis, describing AMI recording in MINAP and HES in the overall study population at risk of or with CKD. Areas of Venn diagrams are proportional to the number of cases.

For peer review only

Supplementary table 1: ICD-10 codes for AMI identified in HES

**AMI subtype**	**ICD-10 codes**
Overall	I21, I22, I23
STEMI	I21.0, I21.1, I21.2, I21.3, I22.0, I22.1, I22.8
NSTEMI	I21.4, I21.9, I22.9
Missing	I23

AMI: acute myocardial infarction; HES: Hospital Episode Statistics; ICD-10: International Classification of Diseases 10th Edition; STEMI: ST elevated myocardial infarction; NSTEMI: Non-ST elevated myocardial infarction

For peer review only

Supplementary table 2: CALIBER definition of AMI subtypes (STEMI, NSTEMI) using MINAP data.

	MINAP variable		
**AMI subtype**	**Discharge diagnosis**	**Markers elevated?**	**ECG result**
Other	Threatened MI, Chest pain uncertain cause, MI unconfirmed, other diagnosis	-	-
STEMI	STEMI	Raised or missing	ST elevation, LBBB, or ST elevation
	NSTEMI/Troponin positive ACS	Raised or missing	ST elevation
	ACS troponin negative	Raised	ST elevation
	ACS troponin unspecified	Raised	ST elevation
NSTEMI	NSTEMI/Troponin positive ACS	Raised or missing	ST depression, T wave changes only, other abnormality, Normal ECG, or LBBB
	ACS troponin negative	Raised	LBBB, ST depression, T wave changes only, Other abnormality, normal ECG, or missing
	ACS troponin unspecified	Raised	LBBB, ST depression, T wave changes only, Other abnormality, normal ECG, or missing
Unstable angina	*Any remaining hospitalisations not assigned as STEMI, NSTEMI, or other diagnosis		

ACS: acute coronary syndrome, AMI: acute myocardial infarction, ECG: electrocardiogram, LBBB: left bundle branch block, MI: myocardial infarction, MINAP: Myocardial Ischaemia National Audit Project, NSTEMI: Non ST-elevation myocardial infarction, STEMI: ST-elevation myocardial infarction

For peer review only

Supplementary table 3: Variables used to define death in-hospital and post-AMI discharge.

**Data source**	**Variables to define in-hospital death**	**Variables to define death post-AMI discharge (in those who survive first AMI hospitalization)**
ONS	Date of death (falling on or in between first AMI admission and discharge dates)	Date of death (falling after first AMI discharge date)
MINAP	Reason for no angiography, no intervention, admission ward, discharge destination	-
HES	Discharge location, discharge method	-

AMI: acute myocardial infarction, HES: Hospital Episode Statistics, MINAP: Myocardial Ischaemia National Audit Project, ONS: Office of National Statistics

For peer review only

Supplementary table 4: Details on data sources for covariates.

Category	Covariate	Data source
Sociodemographic and lifestyle variables	Age at AMI admission	NCKDA
	Sex	NCKDA
	IMD quintiles	NCKDA
	Smoking status	NCKDA, MINAP
Comorbidities	Angina	MINAP and HES
	Cerebrovascular disease	MINAP and HES
	COPD	MINAP and HES
	Diabetes mellitus	MINAP and HES
	Heart failure	MINAP and HES
	Hypertension	NCKDA, MINAP, and HES
	Previous myocardial infarction	MINAP, and HES
	Peripheral vascular disease	NCKDA, MINAP, and HES
	Dialysis	NCKDA
	Kidney transplant	NCKDA

AMI: Acute Myocardial Infarction, COPD: Chronic Obstructive Pulmonary Disease, HES: Hospital Episode Statistics, IMD: Index of Multiple Deprivation, NCKDA: National Chronic Kidney Disease Audit, MINAP: Myocardial Ischaemia National Audit Project

For peer review only

Supplementary table 5: AMI database capture by CKD status (binary) stratified by AMI subtype (STEMI or NSTEMI)

AMI subtype	Database (outcome, compared to people with AMI recorded in MINAP & HES)	CKD stage (exposure)	Number of AMI admissions	Unadjusted RRR (95% CI)	Adjusted RRR[1] (95% CI)
**STEMI**	**MINAP only** (N=115)	At risk of CKD & Stages 1-2	31	1	1
		Stage 3a-5	74	1.08 (0.68-1.72)	0.88 (0.52-1.49)
	**HES only** (N=1,482)	At risk of CKD & Stages 1-2	1,306	1	1
		Stage 3a-5	194	0.07 (0.05-0.09)	0.07 (0.05-0.09)
**NSTEMI**	**MINAP only** (N=666)	At risk of CKD & Stages 1-2	167	1	1
		Stage 3a-5	474	1.16 (0.93-1.45)	1.11 (0.88-1.41)
	**HES only** (N=2,834)	At risk of CKD & Stages 1-2	2,164	1	1
		Stage 3a-5	717	0.14 (0.12-0.16)	0.15 (0.12-0.17)

[1] adjusted for sex, age group at AMI admission, ethnicity, previous AMI, and clustering by patient

AMI: Acute Myocardial Infarction, CKD: chronic kidney disease, HES: Hospital Episode Statistics, MINAP: Myocardial Ischaemia National Audit Project, NSTEMI: non-ST elevation myocardial infarction, RRR: relative risk ratio, STEMI: ST-elevation myocardial infarction

For peer review only

Supplementary table 6: Serum creatinine (SCr) measured in the first 24 hours of AMI hospitalization recorded in MINAP, stratified by CKD stage defined in primary care (NCKDA)

**CKD status (NCKDA)**	**SCr recorded in MINAP at AMI admission, n (%)**	**MINAP median SCr, umol (IQR)**	**MINAP Median SCr value*, mg/dL (IQR)**	**MINAP Median eGFR, mL/min/1.73m^2 (IQR)**
All stages	2,240 (97)	115 (91-152)	1.30 (1.03-1.72)	47.6 (33.5-61.6)
At risk of CKD & Stages 1-2	630 (97)	88 (74-104)	1.00 (0.84-1.18)	67.0 (56.0-80.2)
3a	770 (97)	109 (94-128)	1.23 (1.06-1.45)	50.8 (41.9-59.2)
3b	587 (98)	140 (117-174)	1.58 (1.32-1.97)	36.7 (29.4-43.4)
4-5	253 (95)	255 (190-407)	2.88 (2.15-4.60)	19.8 (12.0-25.5)

*Multiplied by 0.95 for SCr taken before 2013

AMI: acute myocardial infarction, CKD: chronic kidney disease, eGFR: estimated glomerular filtration rate, NCKDA: National Chronic Kidney Disease Audit, MINAP: Myocardial Ischaemia National Audit Project, SCr: serum creatinine

For peer review only

Supplementary table 7: AMI hospitalisations occurring before study start – description of people in the NCKDA who experienced AMI hospitalisation recorded in MINAP or HES before the study start. n (column %) unless specified otherwise.

**CKD status** (main exposure)	**No CKD**	**CKD**			**Total**
**CKD stage** (secondary exposure)	**At-risk of CKD & 1-2**	**3a**	**3b**	**4-5**	
**Unique individuals**	27,909	4,884	2,083	726	34,860
**Total number of AMI events, N**	32,041	5,641	2,460	926	41,068
**Age at AMI event, years, mean (SD)**	64 (13)	75 (10)	78 (10)	75 (12)	66 (13)
**Age category at AMI event, years**					
18-50	4,506 (14)	83 (1)	22 (1)	34 (4)	4,645 (11)
50-64	11,964 (37)	829 (15)	207 (8)	130 (14)	13,130 (32)
65-79	11,517 (36)	2,777 (49)	986 (40)	405 (44)	15,685 (38)
80+	3,922 (12)	1,952 (35)	1,245 (51)	357 (39)	7,476 (18)
Missing	132 (0)	0 (0)	0 (0)	0 (0)	132 (0)
**Female**	9,288 (29)	2,482 (44)	1,209 (49)	394 (43)	13,373 (33)
**Ethnicity**					
White	29,016 (91)	5,228 (93)	2,257 (92)	846 (91)	37,347 (91)
Other	2,612 (8)	295 (5)	139 (6)	71 (8)	3,117 (8)
Missing	413 (1)	118 (2)	64 (3)	9 (1)	604 (1)
**IMD quintile**					
1 (least deprived)	5,808 (18)	1,121 (20)	456 (19)	184 (20)	7,569 (18)
2	6,716 (21)	1,284 (23)	523 (21)	182 (20)	8,705 (21)
3	6,922 (22)	1,297 (23)	542 (22)	180 (19)	8,941 (22)
4	6,820 (21)	1,134 (20)	525 (21)	216 (23)	8,695 (21)
5 (most deprived)	5,531 (17)	787 (14)	406 (17)	161 (17)	6,885 (17)
Missing	244 (1)	18 (0)	8 (0)	3 (0)	273 (1)
**Dialysis in primary care**					
Peritoneal dialysis	0 (0)	0 (0)	0 (0)	12 (1)	12 (0)
Haemodialysis	0 (0)	0 (0)	0 (0)	24 (3)	24 (0)
Renal dialysis, unspecified	0 (0)	0 (0)	0 (0)	11 (1)	11 (0)
**Kidney transplant**	3 (0)	4 (0)	8 (0)	11 (1)	26 (0)
**Comorbidities**					
Angina	5,001 (16)	1,630 (29)	882 (36)	335 (36)	7,848 (19)
Cerebrovascular disease	1,405 (4)	497 (9)	309 (13)	135 (15)	2,346 (6)

For peer review only

CKD status (main exposure)	No CKD	CKD			Total
**CKD stage** (secondary exposure)	**At-risk of CKD & 1-2**	**3a**	**3b**	**4-5**	
**Unique individuals**	27,909	4,884	2,083	726	34,860
**Total number of AMI events, N**	32,041	5,641	2,460	926	41,068
COPD	2,059 (6)	680 (12)	368 (15)	147 (16)	3,254 (8)
Diabetes	5,823 (18)	1,500 (27)	873 (35)	463 (50)	8,659 (21)
Heart failure	1,228 (4)	456 (8)	353 (14)	199 (21)	2,236 (5)
Hypertension	10,556 (33)	3,287 (58)	1,721 (70)	673 (73)	16,237 (40)
Myocardial infarction	5,563 (17)	1,510 (27)	832 (34)	380 (41)	8,285 (20)
Peripheral vascular disease	799 (2)	258 (5)	181 (7)	106 (11)	1,344 (3)
**Smoking status**					
non-smoker	12,945 (40)	2,049 (36)	835 (34)	301 (33)	16,130 (39)
ever-smoker	16,626 (52)	2,477 (44)	983 (40)	382 (41)	20,468 (50)
Missing	2,470 (8)	1,115 (20)	642 (26)	243 (26)	4,470 (11)

AMI: Acute Myocardial Infarction, CKD: chronic kidney disease, COPD: Chronic Obstructive Pulmonary Disease, HES: Hospital Episode Statistics, IMD: Index of Multiple Deprivation, NCKDA: National Chronic Kidney Disease Audit, MINAP: Myocardial Ischaemia National Audit Project

For peer review only

Supplementary table 8: AMI hospitalisations occurring before study start - multinomial logistic regression comparing the relative risk ratios (RRR) of AMI recording across HES and MINAP according to CKD stage. The comparator outcome is people with AMI recorded in both HES and MINAP databases.

**AMI recording** (outcome, compared to people with AMI recorded in MINAP & HES)	**CKD stage** (exposure)	**Number of AMI admissions, n=**	**Unadjusted[1] RRR (95% CI)**	**Partially adjusted[2] RRR (95% CI)**	**Adjusted[3] RRR (95% CI)**
**MINAP only** (N=3,957)	At-risk/Stages 1-2	2,147	1	1	1
	Stage 3a	999	0.96 (0.87-1.05)	0.90 (0.82-0.99)	0.88 (0.80-0.97)
	Stage 3b	578	1.22 (1.09-1.36)	1.12 (0.99-1.26)	1.05 (0.93-1.19)
	Stages 4-5	233	1.35 (1.14-1.60)	1.27 (1.07-1.51)	1.13 (0.94-1.35)
**HES only** (N=26,697)	At-risk/Stages 1-2	23,904	1	1	1
	Stage 3a	1,911	0.16 (0.15-0.18)	0.23 (0.22-0.25)	0.24 (0.22-0.25)
	Stage 3b	630	0.12 (0.11-0.13)	0.20 (0.17-0.22)	0.21 (0.18-0.23)
	Stages 4-5	252	0.13 (0.11-0.15)	0.18 (0.15-0.22)	0.20 (0.17-0.24)

[1]Complete cases for adjusted model
[2]Adjusted for sex, age at AMI admission, ethnicity (white, other), IMD quintile, clustering by participant
[3]Additionally adjusted for comorbidities previous AMI, heart failure, COPD, diabetes mellitus

AMI: Acute Myocardial Infarction, CKD: chronic kidney disease, COPD: Chronic Obstructive Pulmonary Disease, HES: Hospital Episode Statistics, IMD: Index of Multiple Deprivation, MINAP: Myocardial Ischaemia National Audit Project, RRR: relative risk ratio

For peer review only

Supplementary table 9: AMI hospitalisations occurring before study start - agreement between primary care-derived CKD stage (NCKDA) and secondary care-derived CKD stage (MINAP)

	% agreement	Kappa statistic (SE)
**Overall[1]**	66.4%	0.51 (0.007)
**CKD stages 3a, 3b, 4-5[2]**	57.8%	0.37 (0.008)
**CKD stages 1-2, 3a-5[3]**	80.3%	0.59 (0.010)
**Overall[1], by time from NCKDA SCr test (primary care) to MINAP SCr test (at AMI secondary care admission)**		
0-5 months	66.7%	0.52 (0.009)
6-11 months	65.6%	0.49 (0.013)
12-23 months	63.3%	0.42 (0.023)
24-36 months	65.0%	0.45 (0.034)

[1]Overall agreement when grouping as (1) Stages 1-2 (eGFR 60-120mL/min/1.73m^2), (2) Stage 3a (eGFR 45-59mL/min/1.73m^2), (3) Stage 3b (eGFR 30-44 mL/min/1.73m^2), (4) Stages 4-5 (eGFR 0-30 mL/min/1.73m^2)

[2]Agreement when restricting to people with CKD stages 3a-5, grouped as (1) Stage 3a (eGFR 45-59mL/min/1.73m^2), (2) Stage 3b (eGFR 30-44 mL/min/1.73m^2), (3) Stages 4-5 (eGFR 0-30 mL/min/1.73m^2)

[3]Agreement when grouping as (1) Stages 1-2 (eGFR 60-120mL/min/1.73m^2) and (2) Stages 3a-5 (eGFR 0-59mL/min/1.73m^2)

AMI: Acute Myocardial Infarction, CKD: chronic kidney disease, NCKDA: National Chronic Kidney Disease Audit, MINAP: Myocardial Ischaemia National Audit Project, SCr: serum creatinine

For peer review only

Supplementary table 10: Multinomial logistic regression comparing the relative risk ratios (RRR) of AMI recording across HES and MINAP according to CKD stage, after excluding those with a history of dialysis. The comparator outcome is people with AMI recorded in both HES and MINAP databases.

**AMI recording** (outcome, compared to people with AMI recorded in MINAP & HES)	**CKD stage** (exposure)	**Number of AMI admissions, n=**	**Unadjusted[1] RRR (95% CI)**	**Partially adjusted[2] RRR (95% CI)**	**Adjusted[3] RRR (95% CI)**
**MINAP only** (N=731)	At-risk/Stages 1-2	196	1	1	1
	Stage 3a	245	1.07 (0.85-1.34)	0.98 (0.77-1.25)	0.98 (0.77-1.25)
	Stage 3b	197	1.17 (0.92-1.49)	1.04 (0.80-1.36)	1.03 (0.79-1.35)
	Stages 4-5	93	1.54 (1.14-1.60)	1.41 (1.01-1.97)	1.36 (0.97-1.92)
**HES only** (N=4,353)	At-risk/Stages 1-2	3,456	1	1	1
	Stage 3a	557	0.14 (0.12-0.16)	0.14 (0.12-0.17)	0.14 (0.12-0.16)
	Stage 3b	224	0.08 (0.06-0.09)	0.08 (0.07-0.10)	0.08 (0.06-0.10)
	Stages 4-5	116	0.11 (0.08-0.14)	0.12 (0.09-0.15)	0.12 (0.09-0.16)

[1]Complete cases for adjusted model
[2]Adjusted for sex, age at AMI admission, ethnicity (white, other), IMD quintile, clustering by participant
[3]Additionally adjusted for comorbidities previous AMI, heart failure, COPD, diabetes mellitus

AMI: Acute Myocardial Infarction, CKD: chronic kidney disease, COPD: Chronic Obstructive Pulmonary Disease, HES: Hospital Episode Statistics, IMD: Index of Multiple Deprivation, MINAP: Myocardial Ischaemia National Audit Project, RRR: relative risk ratio

For peer review only

Supplementary table 11: Death during and after first AMI hospitalisation in study population at risk of or with CKD, excluding those with a history of dialysis.

**Death during first AMI hospitalisation (N=5,919)[1]**	**Number who died, n (%)**	-	**Unadjusted OR (95% CI)**	**Adjusted[2] OR (95% CI)**
MINAP & HES	208 (15)	-	1	1
MINAP only	148 (23)	-	1.65 (1.31-2.09)	1.59 (1.25-2.03)
HES only	575 (15)	-	0.97 (0.82-1.15)	1.61 (1.32-1.96)
**Death during complete follow-up in those who survive first AMI hospitalisation (N=5,009)[1]**	**Number who died during follow-up, n**	**Rate per 100 person-years (95% CI)**	**Unadjusted HR (95% CI)**	**Adjusted[2] HR (95% CI)**
MINAP & HES	445	17.5 (16.0-19.2)	1	1
MINAP only	234	23.4 (20.6-26.6)	1.30 (1.11-1.52)	1.14 (0.97-1.34)
HES only	841	10.3 (9.58-11.0)	0.61 (0.54-0.68)	1.09 (0.96-1.24)

[1]Complete cases for adjusted model
[2]Adjusted for sex, age at AMI admission, ethnicity (white, other), IMD quintile, previous AMI, heart failure, COPD, diabetes mellitus

AMI: Acute Myocardial Infarction, CKD: chronic kidney disease, COPD: Chronic Obstructive Pulmonary Disease, HES: Hospital Episode Statistics, HR: hazard ratio, IMD: Index of Multiple Deprivation, NCKDA: National Chronic Kidney Disease Audit, MINAP: Myocardial Ischaemia National Audit Project, OR: odds ratio

For peer review only

Supplementary table 12: Sensitivity analysis describing the ICD-10 diagnoses (first episode, first diagnostic position) of non-AMI HES cases that are within ±30 days of MINAP only AMI cases from the main analyses

**Main analysis**		
**MINAP only AMI hospitalisations matched with non-AMI HES hospitalisations**		677/766 (88%)
**Short ICD-10 code (1st episode, 1st diagnostic position)**		**n (% of matched)**
I24	Other acute ischaemic heart diseases	114 (17)
I25	Chronic ischaemic heart disease	84 (12)
R07	Pain in throat and chest	63 (9)
J18	Pneumonia, organism unspecified	56 (8)
I20	Angina pectoris	51 (8)
I50	Heart failure	39 (6)
S72	Fracture of femur	18 (3)
N39	Other disorders of urinary system	14 (2)
A41	Other sepsis	11 (2)
R06	Abnormalities of breathing	11 (2)
**Sensitivity analysis – AMI hospitalisations occurring before study start**		
**MINAP only AMI hospitalisations matched with non-AMI HES hospitalisations**		3,908/4,470 (87%)
**Short ICD-10 code (1st episode, 1st diagnostic position)**		**n (% of matched)**
I25	Chronic ischaemic heart disease	996 (25)
I20	Angina pectoris	760 (19)
R07	Pain in throat and chest	588 (15)
I24	Other acute ischaemic heart diseases	271 (7)
I50	Heart failure	129 (3)

For peer review only

J18	Pneumonia, organism unspecified	86 (2)
I48	Atrial fibrillation and flutter	73 (2)
R06	Abnormalities of breathing	49 (1)
I46	Cardiac arrest	42 (1)
S72	Fracture of femur	40 (1)

AMI: Acute Myocardial Infarction, HES: Hospital Episode Statistics, ICD-10: International Classification of Diseases – 10th Edition, MINAP: Myocardial Ischaemia National Audit Project

For peer review only

Supplementary figure 1: Flow diagram illustrating the NCKDA extracts, and resulting contribution of AMI events to this study

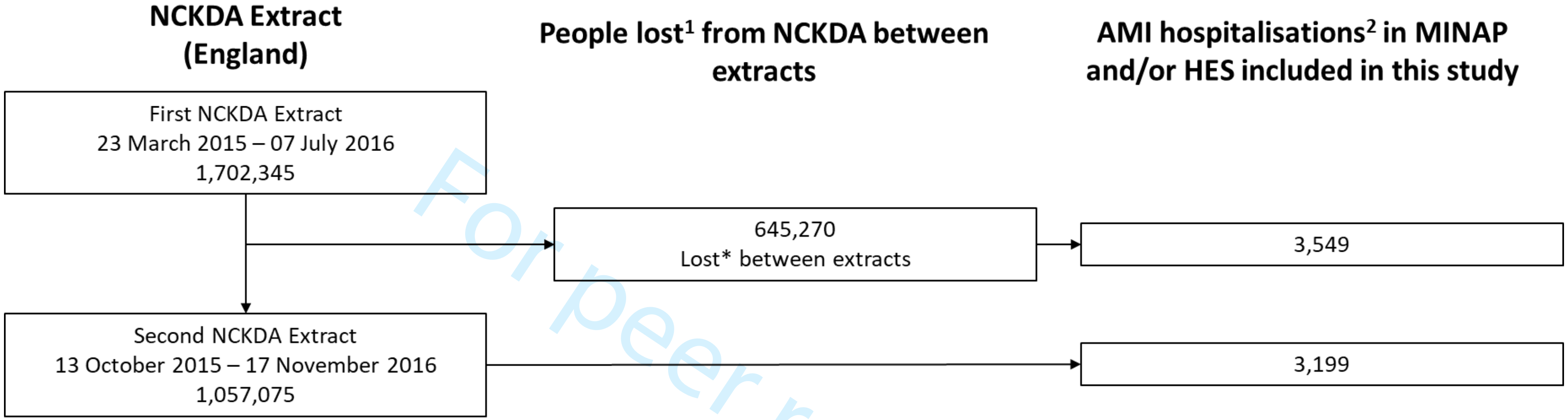

[1]Lost due to death, GP opted out, or person moved GPs

[2]AMI hospitalisations occurring after final NCKDA extract in which each person appears

AMI: acute myocardial infarction, GP: General Practice, NCKDA: National Chronic Kidney Disease Audit, MINAP: Myocardial Ischaemia National Audit Project

Supplementary figure 2: AMI case ascertainment in MINAP and HES datasets over time.

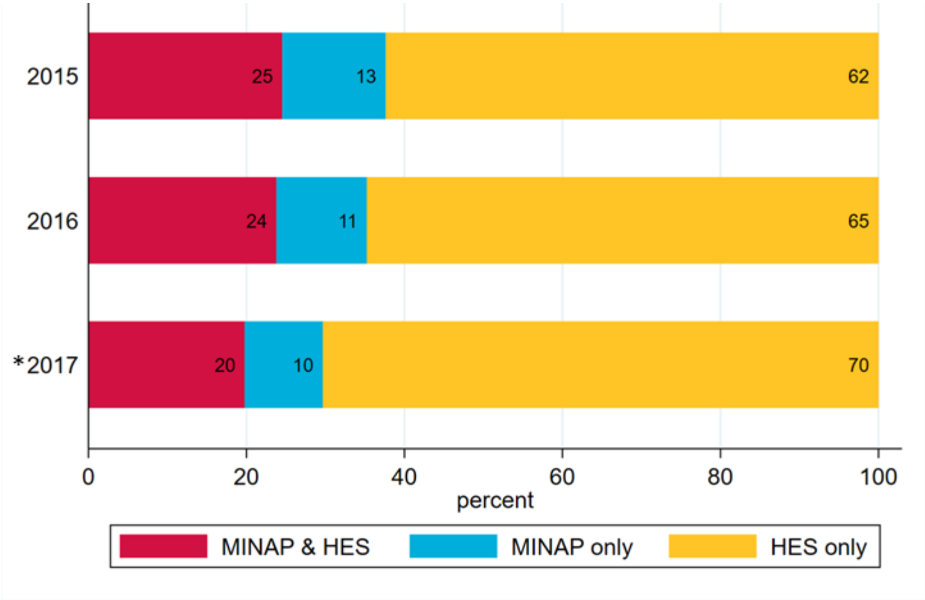

*2017 includes AMI hospitalisations up to 31 March only

AMI: acute myocardial infarction, HES: Hospital Episode Statistics, MINAP: Myocardial Ischaemia National Audit Project

Supplementary figure 3: Bland-Altman plot comparing the mean eGFR (NCKDA eGFR and MINAP eGFR, x-axis) and the difference between the NCKDA eGFR and the MINAP eGFR (y-axis).

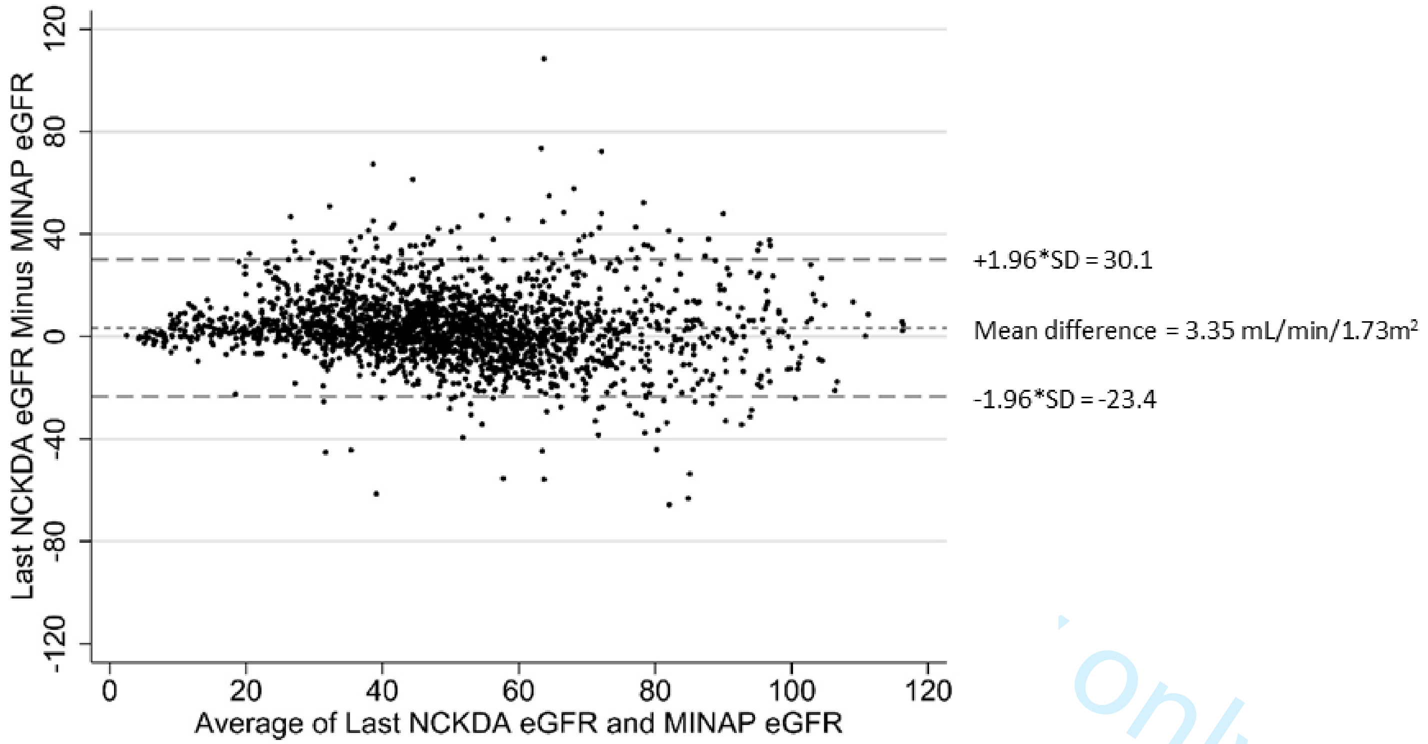

eGFR: estimated glomerular filtration rate, NCKDA: National Chronic Kidney Disease Audit, MINAP: Myocardial Ischaemia National Audit Project

Supplementary figure 4: AMI hospitalisations occurring before study start - comparing AMI case ascertainment overall and by CKD status for AMI hospitalisations in the main analysis and AMI hospitalisations which occurred prior to the last NCKDA extract (sensitivity analysis).

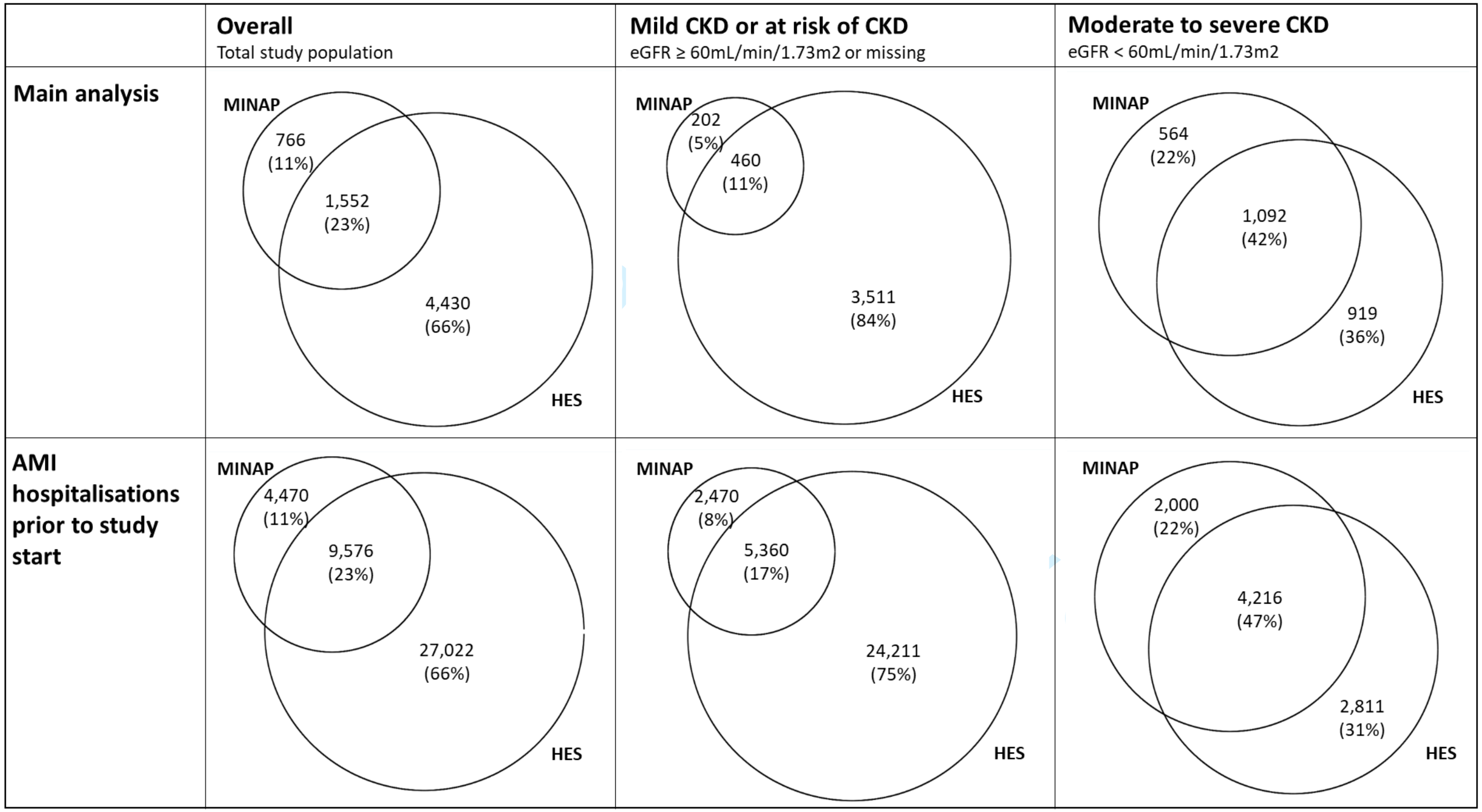

AMI: Acute Myocardial Infarction, CKD: chronic kidney disease, eGFR: estimated glomerular filtration rate, NCKDA: National Chronic Kidney Disease Audit, MINAP: Myocardial Ischaemia National Audit Project

Supplementary figure 5: Sensitivity analyses compared to main analysis, describing AMI recording in MINAP and HES in the overall study population at risk of or with CKD. Areas of Venn diagrams are proportional to the number of cases.

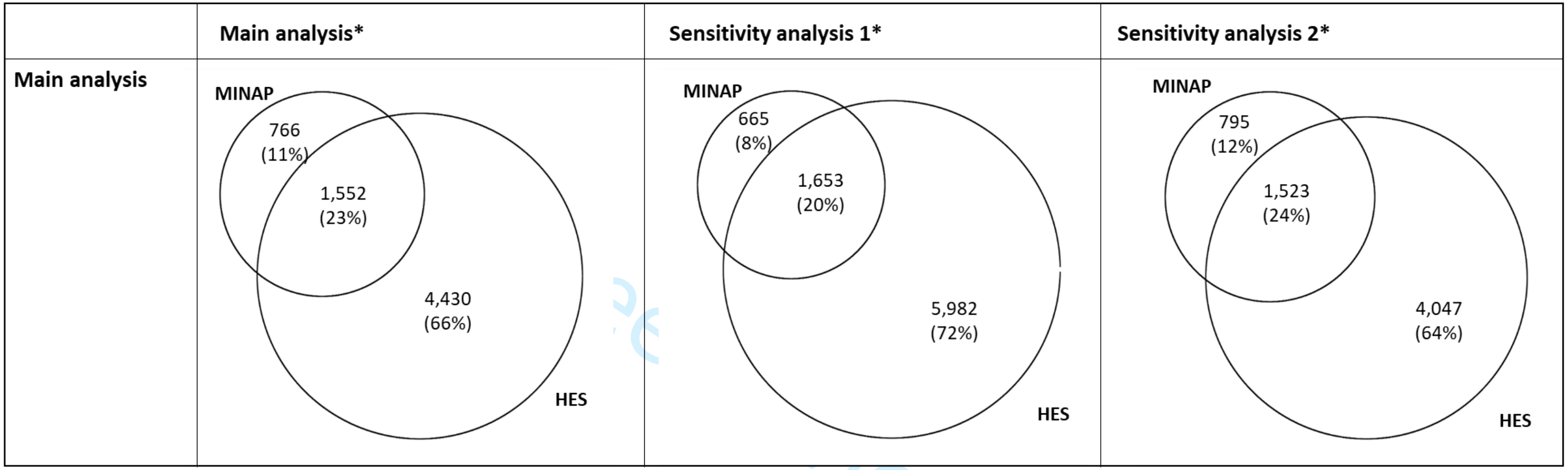

***Main analysis:** HES AMI cases defined as AMI recorded in 1st diagnostic position, 1st episode of hospitalisation
***Sensitivity analysis 1:** HES AMI cases defined as AMI recorded in 1st or 2nd diagnostic position, 1st episode of hospitalisation
***Sensitivity analysis 2:** HES AMI cases in 1st diagnostic position, 1st episode of hospitalisation (as per main analysis) within 30 days of each other counted as a single AMI case, then matched to MINAP AMI cases

AMI: Acute Myocardial Infarction, CKD: chronic kidney disease, HES: Hospital Episode Statistics, MINAP: Myocardial Ischaemia National Audit Project

STROBE 2007 (v4) Statement—Checklist of items that should be included in reports of *cohort studies*

Section/Topic	Item #	Recommendation	Reported on page #
**Title and abstract**	1	(*a*) Indicate the study's design with a commonly used term in the title or the abstract	1
		(*b*) Provide in the abstract an informative and balanced summary of what was done and what was found	4-5
**Introduction**			
Background/rationale	2	Explain the scientific background and rationale for the investigation being reported	7-8
Objectives	3	State specific objectives, including any prespecified hypotheses	8
**Methods**			
Study design	4	Present key elements of study design early in the paper	8-10
Setting	5	Describe the setting, locations, and relevant dates, including periods of recruitment, exposure, follow-up, and data collection	8-9
Participants	6	(*a*) Give the eligibility criteria, and the sources and methods of selection of participants. Describe methods of follow-up	9-10
		(*b*) For matched studies, give matching criteria and number of exposed and unexposed	N/a
Variables	7	Clearly define all outcomes, exposures, predictors, potential confounders, and effect modifiers. Give diagnostic criteria, if applicable	10-13
Data sources/ measurement	8*	For each variable of interest, give sources of data and details of methods of assessment (measurement). Describe comparability of assessment methods if there is more than one group	12-13
Bias	9	Describe any efforts to address potential sources of bias	13-15, 21-22
Study size	10	Explain how the study size was arrived at	8-9
Quantitative variables	11	Explain how quantitative variables were handled in the analyses. If applicable, describe which groupings were chosen and why	12
Statistical methods	12	(*a*) Describe all statistical methods, including those used to control for confounding	13-16
		(*b*) Describe any methods used to examine subgroups and interactions	13-16
		(*c*) Explain how missing data were addressed	15-16
		(*d*) If applicable, explain how loss to follow-up was addressed	14
		(*e*) Describe any sensitivity analyses	15
**Results**			

For peer review only

Participants	13*	(a) Report numbers of individuals at each stage of study—eg numbers potentially eligible, examined for eligibility, confirmed eligible, included in the study, completing follow-up, and analysed	17
		(b) Give reasons for non-participation at each stage	N/a
		(c) Consider use of a flow diagram	Supp figure 1
Descriptive data	14*	(a) Give characteristics of study participants (eg demographic, clinical, social) and information on exposures and potential confounders	17, Table 1
		(b) Indicate number of participants with missing data for each variable of interest	Table 1
		(c) Summarise follow-up time (eg, average and total amount)	18, supp figure 1
Outcome data	15*	Report numbers of outcome events or summary measures over time	17-18
Main results	16	(*a*) Give unadjusted estimates and, if applicable, confounder-adjusted estimates and their precision (eg, 95% confidence interval). Make clear which confounders were adjusted for and why they were included	17-18, Table 2
		(*b*) Report category boundaries when continuous variables were categorized	Table 2
		(*c*) If relevant, consider translating estimates of relative risk into absolute risk for a meaningful time period	N/a
Other analyses	17	Report other analyses done—eg analyses of subgroups and interactions, and sensitivity analyses	18-20
**Discussion**			
Key results	18	Summarise key results with reference to study objectives	20-21
**Limitations**			
Interpretation	20	Give a cautious overall interpretation of results considering objectives, limitations, multiplicity of analyses, results from similar studies, and other relevant evidence	23-24
Generalisability	21	Discuss the generalisability (external validity) of the study results	23-24
**Other information**			
Funding	22	Give the source of funding and the role of the funders for the present study and, if applicable, for the original study on which the present article is based	27

*Give information separately for cases and controls in case-control studies and, if applicable, for exposed and unexposed groups in cohort and cross-sectional studies.

Note: An Explanation and Elaboration article discusses each checklist item and gives methodological background and published examples of transparent reporting. The STROBE checklist is best used in conjunction with this article (freely available on the Web sites of PLoS Medicine at http://www.plosmedicine.org/, Annals of Internal Medicine at http://www.annals.org/, and Epidemiology at http://www.epidem.com/). Information on the STROBE Initiative is available at www.strobe-statement.org.

For peer review only

BMJ Open

The impact of chronic kidney disease on case ascertainment for hospitalised acute myocardial infarction: An English cohort study

Journal:	*BMJ Open*
Manuscript ID	bmjopen-2021-057909.R2
Article Type:	Original research
Date Submitted by the Author:	18-Feb-2022
Complete List of Authors:	Bidulka, Patrick; London School of Hygiene and Tropical Medicine Faculty of Epidemiology and Population Health, Non-Communicable Disease Epidemiology<br>Scott, Jemima; University of Bristol, Population Health Sciences; North Bristol NHS Trust, Richard Bright Renal Service<br>Taylor, Dominic ; University of Bristol, Population Health Sciences; North Bristol NHS Trust<br>Udayaraj, Udaya; Churchill Hospital, Oxford Kidney Unit; University of Oxford, Nuffield Department of Medicine<br>Caskey, Fergus; University of Bristol, Population Health Sciences; North Bristol NHS Trust, Richard Bright Renal Service<br>Teece, Lucy; University of Leicester, Biostatistics Research Group<br>Sweeting, Michael; University of Leicester, Biostatistics Research Group<br>Deanfield, John; Barts Health NHS Trust, National Institute for Cardiovascular Outcomes Research (NICOR); University College London, Institute of Cardiovascular Sciences<br>de Belder, Mark; Barts Health NHS Trust, National Institute for Cardiovascular Outcomes Research (NICOR)<br>Denaxas, S; University College London, Institute of Health Informatics, Faculty of Population Health Sciences; Health Data Research UK<br>Weston, Clive; Glangwili General Hospital<br>Adlam, David; University of Leicester, Department of Cardiovascular Sciences and NIHR Leicester Biomedical Research Centre<br>Nitsch, Dorothea; London School of Hygiene & Tropical Medicine, Department of Non-Communicable Disease Epidemiology
**Primary Subject Heading**:	Renal medicine
Secondary Subject Heading:	Cardiovascular medicine, Epidemiology, General practice / Family practice, Research methods
Keywords:	CARDIOLOGY, Audit < CARDIOLOGY, Myocardial infarction < CARDIOLOGY, NEPHROLOGY, STATISTICS & RESEARCH METHODS

For peer review only - http://bmjopen.bmj.com/site/about/guidelines.xhtml

I, the Submitting Author has the right to grant and does grant on behalf of all authors of the Work (as defined in the below author licence), an exclusive licence and/or a non-exclusive licence for contributions from authors who are: i) UK Crown employees; ii) where BMJ has agreed a CC-BY licence shall apply, and/or iii) in accordance with the terms applicable for US Federal Government officers or employees acting as part of their official duties; on a worldwide, perpetual, irrevocable, royalty-free basis to BMJ Publishing Group Ltd ("BMJ") its licensees and where the relevant Journal is co-owned by BMJ to the co-owners of the Journal, to publish the Work in this journal and any other BMJ products and to exploit all rights, as set out in our licence.

The Submitting Author accepts and understands that any supply made under these terms is made by BMJ to the Submitting Author unless you are acting as an employee on behalf of your employer or a postgraduate student of an affiliated institution which is paying any applicable article publishing charge ("APC") for Open Access articles. Where the Submitting Author wishes to make the Work available on an Open Access basis (and intends to pay the relevant APC), the terms of reuse of such Open Access shall be governed by a Creative Commons licence – details of these licences and which Creative Commons licence will apply to this Work are set out in our licence referred to above.

Other than as permitted in any relevant BMJ Author's Self Archiving Policies, I confirm this Work has not been accepted for publication elsewhere, is not being considered for publication elsewhere and does not duplicate material already published. I confirm all authors consent to publication of this Work and authorise the granting of this licence.

For peer review only

Title:

The impact of chronic kidney disease on case ascertainment for hospitalised acute myocardial infarction: An English cohort study

Authors:

Patrick Bidulka* (1), Jemima Scott* (2,3), Dominic M Taylor (2,3), Udaya Udayaraj (4), Fergus Caskey (2,3), Lucy Teece (5), Michael Sweeting (5), John Deanfield (6,7), Mark de Belder (6), Spiros Denaxas (8,9), Clive Weston (10), David Adlam* (11), Dorothea Nitsch* (1)

*Equal contribution/joint-authorship

Order	Author	Affiliation	Job title at time of writing	Email
1	Bidulka P*	1	Research Fellow	patrick.bidulka1@lshtm.ac.uk
2	Scott J*	2,3	NIHR Doctoral Research Fellow	jemima.scott@bristol.ac.uk
3	Taylor DM	2,3	Consultant Nephrologist (NBT), Honorary Lecturer (UoB)	dominic.taylor@nbt.nhs.uk
4	Udayarai U	4,5	Consultant Nephrologist Honorary Senior Lecturer	Udaya.Udayaraj@ouh.nhs.uk
5	Caskey FJ	2,3	Consultant senior lecturer	fergus.caskey@bristol.ac.uk
6	Teece L	6	Research Fellow in Medical Statistics	lucy.teece@leicester.ac.uk
7	Sweeting MJ	6	Associate Professor in Biostatistics	michael.sweeting@leicester.ac.uk
8	Deanfield J	7,8	Professor of Cardiology	j.deanfield@ucl.ac.uk
9	de Belder M	7	Professor of Cardiology	mark.debelder@nhs.net
10	Denaxas S	9,10	Professor of Biomedical Informatics	s.denaxas@ucl.ac.uk
11	Weston C	11	Consultant Cardiologist	cliveweston@doctors.org.uk
12	Adlam D	12	Associate Professor in acute and interventional cardiology; Honorary consultant	da134@leicester.ac.uk
13	Nitsch D	1	Professor	dorothea.nitsch@lshtm.ac.uk

For peer review only - http://bmjopen.bmj.com/site/about/guidelines.xhtml

Affiliations:

(1) Department of Non-Communicable Disease Epidemiology, London School of Hygiene & Tropical Medicine, Keppel Street, London UK WC1E 7HT

(2) Population Health Sciences, University of Bristol, Bristol, UK BS8 2PS

(3) Richard Bright Renal Service, North Bristol NHS Trust, Southmead Hospital, Bristol BS10 5NB

(4) Oxford Kidney Unit, Churchill Hospital, Oxford OX3 7LE

(5) Nuffield Department of Medicine, University of Oxford, Oxford, OX3 7BN

(6) Biostatistics Research Group, Department of Health Sciences, University of Leicester, University Road, Leicester, UK, LE1 7RH

(7) National Institute for Cardiovascular Outcomes Research (NICOR), Barts Health NHS Trust, 2nd Floor, 1 St Martin's-Le-Grand, London EC1A 4AS

(8) Institute of Cardiovascular Sciences, University College London. London, UK

(9) Institute of Health Informatics, Faculty of Population Health Sciences, University College London, London UK NW1 2DA

(10) Health Data Research UK, London UK

(11) Glangwili General Hospital, Dolgwili Road, Carmarthen, Wales SA31 2AF

(12) Department of Cardiovascular Sciences, University of Leicester, and NIHR Leicester Biomedical Research Centre, Leicester UK

For peer review only

Word count (abstract): 300

Word count (main text): 4441

Figures: 1

Tables: 4

Corresponding author: patrick.bidulka1@lshtm.ac.uk

For peer review only

For peer review only - http://bmjopen.bmj.com/site/about/guidelines.xhtml

ABSTRACT

Objectives

Acute myocardial infarction (AMI) case ascertainment improves for the UK general population using linked health datasets. Because care pathways for people with chronic kidney disease (CKD) change based on disease severity, AMI case ascertainment for these people may differ compared to the general population. We aimed to determine the association between CKD severity and AMI case ascertainment in two secondary care datasets, and the agreement in estimated glomerular filtration rate (eGFR) between the same datasets.

Methods

We used a cohort study design. Primary care records for people with CKD or risk factors for CKD, identified using the National CKD Audit (2015-2017), were linked to the Myocardial Ischaemia National Audit Programme (MINAP, 2007-2017) and Hospital Episode Statistics (HES, 2007-2017) secondary care registries. People with an AMI recorded in either MINAP, HES, or both were included in the study cohort. CKD status was defined using estimated glomerular filtration rate (eGFR), derived from the most recent serum creatinine value recorded in primary care. Moderate-severe CKD was defined as eGFR<60mL/min/1.73m^2, and mild CKD or at risk of CKD was defined as eGFR≥60mL/min/1.73m^2 or eGFR missing. CKD stages were grouped as (1) At risk of CKD & Stages 1-2 (eGFR missing or ≥60mL/min/1.73m^2), (2) Stage 3a (eGFR 45-59mL/min/1.73m^2), (3) Stage 3b (eGFR 30-44mL/min/1.73m^2), and (4) Stages 4-5 (eGFR<30mL/min/1.73m^2).

For peer review only - http://bmjopen.bmj.com/site/about/guidelines.xhtml

Results

We identified 6,748 AMIs: 23% were recorded in both MINAP and HES, 66% in HES only and 11% in MINAP only. Compared to people at risk of CKD or with mild CKD, AMIs in people with moderate-severe CKD were more likely to be recorded in both MINAP and HES (42% vs. 11% respectively), or MINAP only (22% vs. 5%), and less likely to be recorded in HES only (36% vs. 84%). People with AMIs recorded in HES only or MINAP only had increased odds of death during hospitalisation compared to those recorded in both (adjusted odds ratio (OR) 1.61, 95% confidence interval (CI) 1.32-1.96 and OR 1.60, 95% CI 1.26-2.04, respectively). Agreement between eGFR at AMI admission (MINAP) and in primary care was poor (kappa (K) 0.42, standard error (SE) 0.012).

Conclusions

AMI case ascertainment is incomplete in both MINAP and HES, and is associated with CKD severity.

For peer review only

ARTICLE SUMMARY

- Our study includes a large sample size of 6,748 acute myocardial infarction (AMI) events.
- We have assessed the completeness of AMI hospitalisations recorded in two healthcare datasets widely used in observational research in England.
- We evaluated, for the first time, the validity of using serum creatinine recorded in secondary care at the time of an AMI to estimate pre-AMI CKD stage.
- Generalisability to the general population is limited as the National Chronic Kidney Disease Audit only included people with CKD and/or risk factors for CKD.

For peer review only

For peer review only - http://bmjopen.bmj.com/site/about/guidelines.xhtml

INTRODUCTION

Prognosis following acute myocardial infarction (AMI) has improved considerably over the past 50 years such that 85% of individuals now live longer than one year post-AMI.[1] Improved survival is the result of advances in AMI management, driven by evidence from large-scale randomised controlled trials (RCTs).[2-6] Of those admitted to hospital with AMI, 30-40% have chronic kidney disease (CKD):[7] a sustained reduction in kidney function associated with poor outcomes.[8,9] Amongst those with dialysis-dependent CKD only 40% will survive their first year post-AMI.[10] These inferior outcomes may result from higher prevalence of comorbidity,[2] calcific coronary artery disease[3] and the pro-inflammatory effects of uraemia.[4]

Most major RCTs investigating AMI interventions excluded patients with advanced CKD.[11] However, current AMI guidelines from Europe and the United States apply the results of these RCTs to those with or without CKD.[5-7] Clinicians' unease with the dearth of evidence may explain diversion from these AMI guidelines when treating people with CKD.[2,10,11] In the absence of specific RCTs in CKD populations, well-conducted observational analyses can contribute significantly to our understanding and improved management of AMI.

In the UK, data on AMI treatment and outcomes is collected in unlinked, disease-specific registries or in broad registration databases. While there are known differences in the reliability and validity of AMI case ascertainment using these resources in the general population,[12] it is unclear to what extent these differences persist in people with underlying CKD. Multimorbidity and differences in admission and treatment pathways in people with CKD may influence AMI case recording. Reliably identifying which AMI patients have CKD using AMI audit data is also

For peer review only - http://bmjopen.bmj.com/site/about/guidelines.xhtml

difficult; previous studies used admission serum creatinine (SCr) as a proxy for pre-admission CKD stage.[13-15] This unvalidated method risks misclassifying people as having CKD because of the co-incidence of acute kidney injury (AKI) and AMI.[16]

In this study we linked records from the National Chronic Kidney Disease Audit (NCKDA) to the Myocardial Ischaemia National Audit Project (MINAP) and Hospital Episode Statistics (HES) to determine the reliability of these data sources to investigate cardiovascular disease comorbidity and outcomes in people with or at risk of CKD in England. Our objectives were to: (1) Compare case ascertainment of AMI hospitalisations in secondary care datasets (MINAP and HES); (2) determine if MINAP and/or HES case ascertainment defines populations of CKD patients with different risks of death during and after AMI; and (3) compare CKD stage classification using admission serum creatinine recorded in secondary care (MINAP) versus primary care (NCKDA).

For peer review only

METHODS

Data sources

Data from all sources were restricted to patients treated in England. People with or at risk of CKD were identified using primary care data from the NCKDA.[17,18] The NCKDA aimed to optimise the identification and management of people with CKD and/or risk factors for CKD in primary care, and included 10% of English General Practices (GP).[17,18] NCKDA data were collected between 2015 and 2016 in two main cross-sectional data extracts for people with either blood or urine laboratory results indicating CKD and/or risk factors for CKD (prevalent

For peer review only - http://bmjopen.bmj.com/site/about/guidelines.xhtml

hypertension, diabetes mellitus, cardiovascular disease, connective tissue disorders, kidney stones, prostatic disease, family history of kidney disease, previous acute kidney injury (AKI), and users of kidney-damaging medications such as lithium or calcineurin inhibitors).[17] People without an eGFR recorded in primary care were included in the NCKDA only if they were at risk of CKD.

The population identified from NCKDA were linked with Office of National Statistics (ONS) data, January 1998 to September 2019, as well as secondary care data from HES Admitted patient Care (APC) and MINAP, April 2007 to April 2017. HES APC includes hospital admission data for National Health Service (NHS)-treated patients in England, including admission and discharge dates, and diagnoses recorded using International Classification of Diseases 10th Edition (ICD-10) codes.[19] MINAP is an on-going AMI audit in England, Wales, and Northern Ireland which was designed to optimise the care of patients with type one AMI by evaluating the patient pathway from hospital admission with AMI[20] to discharge. MINAP includes admission and discharge dates, treatments, and comorbidities.[21]

Study design

Cohort study.

Study participants

We included people in the NCKDA registered with a GP in England, with one or more AMI hospitalisation recorded in HES or MINAP. People in each NCKDA extract must have been alive according to GP records at the time of that extract. We therefore included people with an AMI

For peer review only - http://bmjopen.bmj.com/site/about/guidelines.xhtml

hospitalisation recorded in MINAP or HES only after the date of their GP's final NCKDA extract. People with an AMI hospitalisation that started prior to the extract date and ended after the extract date were added to the cohort, since they were at risk of death (n=183). In addition, people with an ONS death date indicating death during an AMI hospitalisation that occurred within 90 days prior to the NCKDA extract date were included (n=96), since they were likely misclassified as alive at the time of the extract because of delays in updating the death date in the GP systems. People with an ONS death date earlier than 90 days prior to the extract were excluded (n=4).

Exposures

Our main exposure variable was moderate to severe CKD (eGFR<60mL/min/1.73m^2, CKD stages 3-5), defined using the most recent eGFR recorded in primary care (NCKDA data) prior to the AMI hospitalisation. People with no eGFR recorded in primary care or an eGFR≥60mL/min/1.73m^2 were categorised as at risk of CKD or having mild CKD, respectively. We assumed people with no eGFR recorded in primary care did not have moderate to severe CKD since these people are much less likely to have CKD than those with eGFR recorded.[22] eGFR was calculated using primary care SCr measures and the revised Modification of Diet in Renal Disease (MDRD) equation.[23,24]

Our secondary exposure was CKD stage, defined by the Kidney Disease Improving Global Outcomes CKD staging, based on a single eGFR record without the requirement for two measures three months apart.[25] We combined some CKD stages due to low numbers of AMI

For peer review only - http://bmjopen.bmj.com/site/about/guidelines.xhtml

cases: (1) At risk of CKD & Stages 1-2 (eGFR missing or ≥60mL/min/1.73m^2), (2) Stage 3a (eGFR 45-59mL/min/1.73m^2), (3) Stage 3b (eGFR 30-44mL/min/1.73m^2), and (4) Stages 4-5 (eGFR<30mL/min/1.73m^2).

We used the latest eGFR recorded prior to the AMI hospitalisation to categorise people with a history of kidney transplant into the primary and secondary exposure groups. We categorised people with a history of dialysis prior to the AMI hospitalisation as moderate to severe CKD for the main exposure and CKD stages 4-5 for the secondary exposure, even if the latest eGFR did not agree.

As the use of a single serum creatinine test at the time of AMI hospitalisation to determine CKD stage has not previously been validated, we have used the term "eGFR stage" in place of CKD stage to refer to the eGFR level calculated from this test.

Outcomes

Primary outcome

The primary outcome was AMI case ascertainment, defined as the dataset(s) in which the AMI hospitalisation was recorded. We defined an AMI as being recorded in both HES and MINAP if an AMI hospitalisation in HES was within 30 days of an AMI hospitalisation in MINAP. Where multiple HES AMI hospitalisations fell within 30 days of a MINAP AMI hospitalisation, the HES AMI hospitalisation closest in time to the MINAP AMI admission was selected as the single

For peer review only - http://bmjopen.bmj.com/site/about/guidelines.xhtml

matched event. AMI hospitalisations without a match were categorised as HES or MINAP only. Study participants could contribute multiple AMI hospitalisations.

We defined an AMI in HES data using ICD-10 codes I.21, I.22, or I.23 in the primary admission diagnosis field (first diagnostic position in the first episode of an admission).[26] We categorised AMI subtype (ST-segment elevation myocardial infarction (STEMI) and non-ST-segment elevation myocardial infarction (NSTEMI)) using the UK Biobank coding definitions[27] (**Supplementary table 1**). We defined AMI in MINAP using a previously developed algorithm which uses the discharge diagnosis, electrocardiogram (ECG) results, and the presence of elevated cardiac markers to identify acute coronary syndrome events and subtypes (**Supplementary table 2**). We excluded MINAP hospitalisations classified as unstable angina or other from the analysis.[12]

Secondary outcomes

We investigated in-hospital mortality during each person's first AMI hospitalisation within the study period. In those who survived and were discharged from their first AMI hospitalisation, we also investigated post-discharge mortality using the ONS death date (up to 15 September 2019). Variables in HES, MINAP, and ONS used to define death are described in **Supplementary table 3**. People were considered to have died during AMI hospitalisation if any of these variables indicated in-hospital death, or the ONS death date fell on or between the admission and discharge dates. We used the earliest of the HES or MINAP admission dates and the latest of the HES or MINAP discharge dates to define these dates for AMI hospitalisations recorded in both datasets.

For peer review only - http://bmjopen.bmj.com/site/about/guidelines.xhtml

We investigated the agreement between CKD stage derived from the most recent primary care SCr test (NCKDA data) and eGFR stage derived from the secondary care SCr test conducted within 24 hours of AMI hospitalisation (MINAP data). We used the same methods to determine eGFR stage in MINAP data as we did for NCKDA data.

Covariates

We described age at AMI admission (mean and standard deviation (SD) as well as age category in years: 18-49, 50-64, 65-79, 80+), sex, ethnicity (white or other), index of multiple deprivation quintiles (IMD, as a proxy for socioeconomic status), and relevant comorbidities including angina, cerebrovascular disease, chronic obstruction pulmonary disease (COPD), diabetes mellitus (type 1 and 2), heart failure, hypertension, previous myocardial infarction, and peripheral vascular disease. We also described dialysis and transplant status, and smoking status. Data sources for each key covariate are described in **Supplementary table 4**.

Data analysis

Objective 1 – AMI case ascertainment

We summarised key covariates by CKD status. We used Venn diagrams to describe AMI case ascertainment overall and stratified by CKD status (at risk of CKD or mild CKD vs. moderate to severe CKD). We used multinomial, multivariable logistic regression to quantify the association between CKD stage and AMI case ascertainment. We used the 'HES and MINAP' category as the

For peer review only - http://bmjopen.bmj.com/site/about/guidelines.xhtml

base outcome and reported crude and adjusted relative risk ratios (RRR) and 95% confidence intervals (CI), using 'At risk of CKD & Stages 1-2' as the reference exposure category. We adjusted for sex, age category, ethnicity, IMD quintile, previous AMI, heart failure, COPD, diabetes mellitus, and clustering by participant (using cluster-robust standard errors (SE)). We used a complete case analysis since we could not assume that missing values for ethnicity were missing at random. In a secondary analysis, we stratified these regressions by AMI subtype (STEMI and NSTEMI).

Objective 2 – Risk of death

We used multivariable logistic regression to calculate the odds of death in hospital during each person's first AMI hospitalisation in people with AMI recorded in MINAP only or HES only, relative to MINAP and HES. After confirming the proportional hazards assumption with a Schoenfeld Residuals test on the full multivariable model (p=0.35), we used multivariable Cox regression to estimate hazards ratios (HR) for death during total follow-up in those who survived their first AMI hospitalisation with AMI recorded in MINAP only or HES only, relative to MINAP and HES.

Objective 3 – Agreement between eGFR in primary and secondary care

Finally, to assess the validity of using MINAP-recorded eGFR at AMI admission as a proxy for pre-admission CKD status, we compared eGFR and its corresponding eGFR stage within 24 hours of AMI admission (MINAP data) to the most recent eGFR and its corresponding CKD stage in primary care (NCKDA data). In this analysis, we excluded people with eGFR measures greater than 120mL/min/1.73m^2 in either NCKDA or MINAP as these are unlikely to be true values. We

For peer review only - http://bmjopen.bmj.com/site/about/guidelines.xhtml

drew a Bland-Altman plot to describe differences in the distribution of eGFR measures in primary and secondary care[28] and calculated the percent agreement and kappa agreement statistics between CKD and eGFR stage derived using primary and secondary care eGFRs, respectively. Secondary analyses re-calculated agreements and kappa statistics restricting to people with stages 3a-5 (eGFR<60mL/min/1.73m^2) in primary care and grouped by time between the most recent primary care eGFR measure and the AMI hospitalisation (0-5, 6-11, 12-23, 24-36-month gaps).

Sensitivity analyses

We repeated the main analyses for AMI events occurring prior to the study start date (the latest NCKDA extract). People who experienced AMI hospitalisation before the study start were survivors, since only people alive at the time of the NCKDA were included in the study.

In addition, we re-drew the Venn diagrams after including HES AMI hospitalisations recorded in both the first and second diagnostic positions of the first episode to include AMIs recorded as co-primary diagnoses.[26] We also repeated the matching process between MINAP and HES AMI hospitalisations after combining all HES AMIs within 30 days of each other into a single HES hospitalisation, since it is likely that some AMI events in our dataset have multiple HES hospitalisations recorded if, for example, a patient is transferred between hospitals for treatment. Furthermore, we repeated our multivariable analyses after excluding people with a history of dialysis. Finally, to understand why people may have an AMI hospitalisation recorded in MINAP but not HES, we searched for non-AMI HES hospitalisations within 30 days of the AMI

For peer review only - http://bmjopen.bmj.com/site/about/guidelines.xhtml

recorded in MINAP and described the ICD-10 diagnoses in the first episode of the first diagnostic position.

Missing data

We did a complete case analysis when building our multivariable models. People with missing ethnicity (~1%) and IMD data (<1%) were excluded prior to building our unadjusted, partially adjusted, and adjusted multinomial models.

We used discharge dates to help re-categorise people who were in-hospital at the time of the NCKDA extract into the cohort, as well as to determine death in hospital and the start of follow-up in those who survived their first AMI hospitalisation. Discharge date was missing in 19% and 1% of the MINAP and HES datasets, respectively. We assumed these dates were missing at random and used the median length of admissions in those without missing admission and discharge dates (5 and 4 days in MINAP and HES, respectively) to impute the missing discharge dates.

Patient and public involvement

The Kidney Care UK patient organisation (https://www.kidneycareuk.org/) supported the research questions, grant applications, and the related record linkage application for section 251 permissions critical to the development of the National Chronic Kidney Disease Audit (NCKDA). Patient members of the UK Renal Registry Patient Council

For peer review only - http://bmjopen.bmj.com/site/about/guidelines.xhtml

For peer review only

(https://renal.org/patients/patient-council) reviewed the study results. Their feedback supported a further planned record linkage of renal and cardiac data to look at patient outcomes.

For peer review only

For peer review only - http://bmjopen.bmj.com/site/about/guidelines.xhtml

RESULTS

Study population and baseline characteristics

From 1,702,345 people in England included in the NCKDA, we identified 6,042 (0.4%) people with or at risk of CKD who experienced 6,748 AMIs between the final NCKDA extract and 1 April 2017 (**Supplementary figure 1**). Baseline characteristics stratified by CKD stage are described in **Table 1**. People with moderate to severe CKD accounted for 38% of AMI hospitalisations (2,575). Average age at the time of AMI was 73 years (SD 13). People with moderate to severe CKD were older on average than people with mild CKD or at risk of CKD. Most people were white (92%) and male (61%). The most prevalent comorbidities were hypertension (61%) and diabetes mellitus (35%).

Table 1: Baseline characteristics by CKD stage for all AMI events captured after the study start. n (column %) unless specified otherwise.

**CKD status** (main exposure)	**At risk of CKD or mild CKD**	**Moderate to severe CKD**			**Total**
**CKD stage** (secondary exposure)	**At risk of CKD & 1-2**	**3a**	**3b**	**4-5**	
**Unique individuals**	3,751	1,210	732	349	6,042
**Total number of AMI events, N**	4,173	1,353	825	397	6,748
**Age at AMI event, years, mean (SD)**	70 (13)	79 (10)	82 (9)	79 (12)	73 (13)
**Age category at AMI event, years**					
18-50	299 (7)	20 (1)	1 (0)	10 (3)	330 (5)
50-64	1,163 (28)	91 (7)	44 (5)	38 (10)	1,336 (20)
65-79	1,655 (40)	531 (39)	251 (30)	122 (31)	2,559 (38)
80+	1,056 (25)	711 (53)	529 (64)	227 (57)	2,523 (37)
**Female**	1,430 (34)	638 (47)	416 (50)	168 (42)	2,652 (39)
**Ethnicity**					
White	3,807 (91)	1,263 (93)	773 (94)	361 (91)	6,204 (92)
Other	323 (8)	73 (5)	44 (5)	34 (9)	474 (7)
Missing	43 (1)	17 (1)	8 (1)	2 (1)	70 (1)
**IMD quintile**					
1 (least deprived)	732 (18)	252 (19)	152 (18)	68 (17)	1,204 (18)
2	843 (20)	306 (23)	176 (21)	85 (21)	1,410 (21)

For peer review only - http://bmjopen.bmj.com/site/about/guidelines.xhtml

CKD status (main exposure)	At risk of CKD or mild CKD	Moderate to severe CKD			Total
**CKD stage** (secondary exposure)	**At risk of CKD & 1-2**	**3a**	**3b**	**4-5**	
**Unique individuals**	3,751	1,210	732	349	6,042
**Total number of AMI events, N**	4,173	1,353	825	397	6,748
3	934 (22)	331 (24)	193 (23)	79 (20)	1,537 (23)
4	951 (23)	255 (19)	185 (22)	96 (24)	1,487 (22)
5 (most deprived)	690 (17)	206 (15)	114 (14)	69 (17)	1,079 (16)
Missing	23 (1)	3 (0)	5 (1)	0 (0)	31 (0)
**Dialysis in primary care**					
Peritoneal dialysis	0 (0)	0 (0)	0 (0)	15 (4)	15 (0)
Haemodialysis	0 (0)	0 (0)	0 (0)	24 (6)	24 (0)
Renal dialysis, unspecified	0 (0)	0 (0)	0 (0)	7 (2)	7 (0)
**Kidney transplant**	5 (0)	0 (0)	4 (0)	17 (4)	26 (0)
**Comorbidities**					
Angina	959 (23)	399 (29)	275 (33)	155 (39)	1,788 (26)
Cerebrovascular disease	390 (9)	178 (13)	139 (17)	81 (20)	788 (12)
COPD	514 (12)	209 (15)	168 (20)	58 (15)	949 (14)
Diabetes mellitus	1,293 (31)	465 (34)	356 (43)	233 (59)	2,347 (35)
Heart failure	400 (10)	234 (17)	211 (26)	123 (31)	968 (14)
Hypertension	2,333 (56)	884 (65)	583 (71)	322 (81)	4,122 (61)
Myocardial infarction	1,050 (25)	430 (32)	274 (33)	163 (41)	1,917 (28)
Peripheral vascular disease	229 (5)	108 (8)	74 (9)	47 (12)	458 (7)
**Smoking status**					
non-smoker	1,953 (47)	566 (42)	306 (37)	151 (38)	2,976 (44)
ever-smoker	2,018 (48)	530 (39)	318 (39)	140 (35)	3,006 (45)
Missing	202 (5)	257 (19)	201 (24)	106 (27)	766 (11)

AMI: Acute Myocardial Infarction, CKD: chronic kidney disease, COPD: Chronic Obstructive Pulmonary Disease, HES: Hospital Episode Statistics, IMD: Index of Multiple Deprivation, NCKDA: National Chronic Kidney Disease Audit, MINAP: Myocardial Ischaemia National Audit Project

For peer review only

AMI recording in HES and MINAP

Overall, 23% of AMI hospitalisations were captured by both MINAP and HES datasets (1,552 AMI hospitalisations) (**Figure 1**). There was no substantial change in AMI case ascertainment over time (**Supplementary figure 2**). In people with moderate to severe CKD, 42% of all AMI hospitalisations were captured by both MINAP and HES (1,092 AMI hospitalisations). In people with mild CKD or at risk of CKD, 11% of all AMI hospitalisations were captured by MINAP and HES (460 AMI hospitalisations) (**Figure 1**).

For peer review only - http://bmjopen.bmj.com/site/about/guidelines.xhtml

Relative association between CKD stage and AMI recording

Crude and adjusted relative risk ratios (RRR) and 95% confidence intervals (CI) describing the association between CKD stage and AMI case ascertainment are presented in **Table 2**. After adjusting for key covariates, we observed weak evidence of an increased likelihood of AMI recorded in MINAP only, compared to MINAP and HES, in people with CKD stages 4-5 vs. the at risk of CKD/stages 1-2 group (RRR 1.34, 95% CI 0.97-1.85). Furthermore, compared to the at-risk of CKD/stages 1-2 group, people with CKD stages 3a, 3b, and 4-5 were less likely to have an AMI hospitalisation recorded in HES only vs. MINAP and HES. We did not observe any differences in the likelihood of recording of AMI hospitalisation when stratifying by AMI subtype (**Supplementary table 5**).

Table 2: Multinomial logistic regression comparing the relative risk ratios (RRR) of AMI recording across HES and MINAP according to CKD stage. The comparator outcome is people with AMI recorded in both HES and MINAP databases.

**AMI recording** (outcome, compared to people with AMI recorded in MINAP & HES)	**CKD stage** (exposure)	**Number of AMI admissions, n=**	**Unadjusted[1] RR (95% CI)**	**Partially adjusted[2] RRR (95% CI)**	**Adjusted[3] RRR (95% CI)**
**MINAP only** (N=742)	At risk of CKD/Stages 1-2	196	1	1	1
	Stage 3a	245	1.07 (0.85-1.34)	0.98 (0.77-1.25)	0.98 (0.77-1.25)
	Stage 3b	197	1.17 (0.92-1.49)	1.04 (0.80-1.36)	1.03 (0.79-1.34)
	Stages 4-5	104	1.50 (1.11-2.03)	1.38 (1.01-1.90)	1.34 (0.97-1.85)
**HES only** (N=4,367)	At risk of CKD/Stages 1-2	3,456	1	1	1

For peer review only - http://bmjopen.bmj.com/site/about/guidelines.xhtml

	Stage 3a	557	0.14 (0.12-0.16)	0.14 (0.12-0.17)	0.14 (0.12-0.16)
	Stage 3b	224	0.08 (0.06-0.09)	0.08 (0.07-0.10)	0.08 (0.06-0.10)
	Stages 4-5	130	0.11 (0.08-0.14)	0.11 (0.09-0.15)	0.12 (0.09-0.16)

[1]Complete cases for adjusted model
[2]Adjusted for sex, age at AMI admission, ethnicity (white, other), IMD quintile, clustering by participant
[3]Additionally adjusted for comorbidities previous AMI, heart failure, COPD, diabetes mellitus

AMI: Acute Myocardial Infarction, CKD: chronic kidney disease, COPD: Chronic Obstructive Pulmonary Disease, HES: Hospital Episode Statistics, IMD: Index of Multiple Deprivation, MINAP: Myocardial Ischaemia National Audit Project, RRR: relative risk ratio

For peer review only

Mortality during AMI hospitalisation and post-discharge

Of those with a first AMI recorded in both HES and MINAP, 209 people (15%) died during the AMI hospitalisation, compared to 151 (23%) with a first AMI recorded in MINAP only and 579 (15%) recorded in HES only (**Table 3**). After adjusting for key covariates, people with AMI recorded in MINAP only and HES only had increased odds of in-hospital death compared to people with AMI recorded in both MINAP and HES (odds ratio (OR) 1.60, 95% CI 1.26-2.04 and OR 1.61, 95% CI 1.32-1.96, respectively).

Mean follow-up among people who survived a first AMI hospitalisation was 2.4 years. The rate of death per 100 person-years (PY) during complete follow-up was 18.0 (95% CI 16.4-19.7) for AMI recorded in MINAP and HES, 23.3 (95% CI 20.6-26.5) for AMI recorded in MINAP only, and 10.3 (95% CI 9.61-11.0) for AMI recorded in HES only (**Table 3**). After adjusting for key covariates, there was no evidence of a difference in death during follow-up based on which dataset(s) captured the first AMI hospitalisation.

For peer review only - http://bmjopen.bmj.com/site/about/guidelines.xhtml

Table 3: Death during and after first AMI hospitalisation in total study population at risk of or with CKD.

**Death during first AMI hospitalisation (N=5,919)[1]**	**Number who died, n (%)**	-	**Unadjusted OR (95% CI)**	**Adjusted[2] OR (95% CI)**
MINAP & HES	209 (15)	-	1	1
MINAP only	151 (23)	-	1.67 (1.32-2.11)	1.60 (1.26-2.04)
HES only	579 (15)	-	0.98 (0.82-1.16)	1.61 (1.32-1.96)
**Death during complete follow-up in those who survive first AMI hospitalisation (N=5,009)[1]**	**Number who died during follow-up, n**	**Rate per 100 person-years (95% CI)**	**Unadjusted HR (95% CI)**	**Adjusted[2] HR (95% CI)**
MINAP & HES	456	18.0 (16.4-19.7)	1	1
MINAP only	237	23.3 (20.6-26.5)	1.27 (1.08-1.48)	1.12 (0.96-1.31)
HES only	847	10.3 (9.61-11.0)	0.59 (0.53-0.67)	1.07 (0.94-1.21)

[1]Complete cases for adjusted model
[2]Adjusted for sex, age at AMI admission, ethnicity (white, other), IMD quintile, previous AMI, heart failure, COPD, diabetes mellitus

AMI: Acute Myocardial Infarction, CKD: chronic kidney disease, COPD: Chronic Obstructive Pulmonary Disease, HES: Hospital Episode Statistics, HR: hazard ratio, IMD: Index of Multiple Deprivation, NCKDA: National Chronic Kidney Disease Audit, MINAP: Myocardial Ischaemia National Audit Project, OR: odds ratio

Agreement between eGFR derived from secondary care vs. primary care data

Of the AMI hospitalisations recorded in MINAP, 2,240 (97%) had SCr recorded within 24 hours of AMI admission (**Supplementary table 6**). Median eGFR at time of admission was 47.6 mL/min/1.73m^2 (IQR 33.5-61.6). The Bland-Altman plot comparing the primary care eGFR and

For peer review only - http://bmjopen.bmj.com/site/about/guidelines.xhtml

the secondary care eGFR indicated a negligible mean difference but wide variation (mean difference 3.35 mL/min/1.73m^2, 95% CI -23.4 to 30.1) (**Supplementary figure 3**).

The percent agreements and kappa statistics between eGFR stage derived from MINAP eGFR at AMI admission and the CKD stage derived from NCKDA using primary care data are shown in **Table 4**. Overall, there was 57.2% agreement in staging (kappa statistic (K) 0.42 (SE 0.012)). When restricting to people with NCKDA-derived CKD stages 3-5, the % agreement and K indicated worse agreement (**Table 4**). However, when looking at agreement in categorising people as having moderate to severe CKD (stages 3-5) vs mild CKD (stages 1-2), agreement improved (82.1% agreement, K 0.55 (SE 0.021)).

When stratifying by months between the primary and secondary care eGFR measures, we observed the best agreement in staging within a 0-5-month gap between the primary and secondary care eGFR measures: 61.0%, K 0.48 (SE 0.03) (**Table 4**). Agreement was worse when the time between eGFR measures increased.

Table 4: Agreement between primary care-derived CKD stage (NCKDA) and secondary care-derived eGFR stage (MINAP)

	**% agreement**	**Kappa statistic (SE)**
**Overall[1]**	57.2%	0.42 (0.012)
**CKD Stages 3a, 3b, 4-5[2]**	53.2%	0.34 (0.015)
**CKD Stages 1-2, 3a-5[3]**	82.1%	0.55 (0.021)
**Overall[1], by time from NCKDA SCr test (primary care) to MINAP SCr test (at AMI secondary care admission)**		
0-5 months	61.0%	0.48 (0.03)
6-11 months	56.7%	0.42 (0.02)
12-23 months	55.9%	0.40 (0.02)
24-36 months	56.8%	0.41 (0.04)

[1]Overall agreement when grouping as (1) Stages 1-2 (eGFR 60-120mL/min/1.73m^2), (2) Stage 3a (eGFR 45-59mL/min/1.73m^2), (3) Stage 3b (eGFR 30-44 mL/min/1.73m^2), (4) Stages 4-5 (eGFR 0-30 mL/min/1.73m^2)

For peer review only - http://bmjopen.bmj.com/site/about/guidelines.xhtml

[2]Agreement when restricting to people with CKD stages 3a-5, grouped as (1) Stage 3a (eGFR 45-59mL/min/1.73m^2), (2) Stage 3b (eGFR 30-44 mL/min/1.73m^2), (3) Stages 4-5 (eGFR 0-30 mL/min/1.73m^2)
[3]Agreement when grouping as (1) Stages 1-2 (eGFR 60-120mL/min/1.73m^2) and (2) Stages 3a-5 (eGFR 0-59mL/min/1.73m^2)

AMI: acute myocardial infarction, CKD: chronic kidney disease, eGFR: estimated glomerular filtration rate, HES: Hospital Episode Statistics, NCKDA: National Chronic Kidney Disease Audit, MINAP: Myocardial Ischaemia National Audit Project, SCr: serum creatinine, SE: standard error

Sensitivity analyses

AMI case ascertainment in MINAP and HES was similar in AMI hospitalisations recorded prior to the study start (sensitivity analysis) compared to after the study start (main analysis) (**Supplementary tables 7-8, Supplementary figure 4**). There were also no major differences in agreement between CKD staging derived in primary vs. secondary care when investigating AMI hospitalisations prior to the study start (**Supplementary table 9**).

After expanding the AMI definition in HES to include any hospitalisations with AMI coded in the second diagnostic position as well as the first, the proportion of AMI hospitalisations captured in both HES and MINAP decreased slightly (**Supplementary figure 5**). After combining HES AMI admissions within 30 days of each other for the same person, we observed a 1% increase in the proportion of AMI hospitalisations recorded in both MINAP and HES (**Supplementary figure 5**). Results were also similar when excluding people with a history of dialysis (**Supplementary tables 10-11**).

Finally, the ten most common diagnoses in HES matching with the MINAP only AMI hospitalisations from the main analysis are shown in **Supplementary table 12**. Eighty-eight

For peer review only - http://bmjopen.bmj.com/site/about/guidelines.xhtml

For peer review only

percent of unmatched MINAP AMIs had a non-AMI HES hospitalisation within 30 days. These were mainly CVD or respiratory infection-related ICD-10 diagnoses.

DISCUSSION

We compared recording of AMI hospitalisations for people with CKD between two large secondary healthcare datasets in England. In a cohort of 6,042 people, we found that both HES and MINAP missed a significant proportion of AMI hospitalisations. CKD stage influenced likelihood of AMI recording by dataset: AMI hospitalisations in people with moderate to severe CKD were more likely to be recorded in MINAP compared to people at risk of CKD or with mild CKD. We found an association between AMI hospitalisation recording by dataset and in-hospital mortality. There was marked variation between eGFR at AMI admission and preceding eGFR measurements in primary care, but no obvious systematic bias in terms of over/underestimation of eGFR at AMI admission.

Our results agree with previous research demonstrating incomplete capture of AMI events by individual healthcare datasets in the overall English population and extend them to a population with CKD. Herrett et al[12] showed 46% agreement when restricting to MINAP and HES recorded AMI hospitalisations, which is close to the 42% agreement we found in people with moderate to severe CKD. A smaller single-centre study by Torabi et al[29] found 32% agreement between MINAP and the hospital information department (responsible for HES coding).

For peer review only - http://bmjopen.bmj.com/site/about/guidelines.xhtml

In contrast to both studies, we found significantly worse agreement in case ascertainment for AMI hospitalisations between MINAP and HES for people at risk of CKD or with mild CKD. Torabi et al[29] collected data on renal function but found likelihood of AMI case recording in MINAP to reduce with advancing CKD stage. Differences in results between this and our study could be ascribed to changes in management of patients and/or event recording over time, differences in the populations studied and local practice in the single centre analysed by Torabi et al.[29]

The high prevalence of CKD risk factors in people at risk of or with mild CKD could put them at greater risk of type 2 AMI; a mismatch of myocardial oxygen supply and demand in the absence of the "classical" coronary artery plaque rupture with thrombosis reflective of type 1 AMI.[20] People with type 2 AMI are typically older, with a greater burden of comorbidities than those with type 1 AMI, and have poor outcomes.[30] HES is likely to include more type 2 AMI than MINAP as clinical coders for the latter are asked to select type 1 AMI only.

People with AMI recorded in both MINAP and HES had lower in-hospital mortality compared with those with AMI recorded in either MINAP or HES only. Our findings agree with Herrett et al;[12] patients with AMI recorded in only one source had a higher mortality than those with events recorded in more than one source. Higher in-hospital mortality in the MINAP only cases is likely to reflect the referral of severe and complex AMI cases to cardiology, including a higher STEMI to NSTEMI ratio.

Across all levels of eGFR, we found significant variation between eGFR stage derived from SCr taken within 24 hours of AMI admission (recorded in MINAP) and that derived from SCr in primary care, which is in line with reported variability of eGFR in validation studies.[23,24] As

For peer review only - http://bmjopen.bmj.com/site/about/guidelines.xhtml

expected with known limitations of using MDRD eGFR to estimate kidney function for GFRs above 60mL/min/1.73m^2, binary classification between individuals with CKD stages 3-5 and those with stages 1-2 is more reliable than classification by CKD stage. These findings suggest that although previous research[13-15] using SCr at AMI admission recorded in MINAP as a proxy for baseline CKD stage may result in misclassification, it is unlikely to have resulted in a systematic bias in either over- or under-estimation of CKD stage, despite our initial hypothesis that there would be systematic underestimation of kidney function due to the substantially increased risk of AKI during an AMI hospitalisation.[31] Differences between SCr recorded in primary care and SCr recorded in MINAP may reflect progression of CKD, differential use of medication that affects the renin-angiotension-aldosterone system, AKI at the time of serum sampling (although changes in serum creatinine are unlikely to show within 24 hours of AMI onset), or variation around the mean.

Limitations

The NCKDA only included people with CKD and/or risk factors for CKD; therefore, we cannot generalise our results to people without risk factors for CKD. We may have incorrectly misclassified people who have no documented tests for CKD in primary care as having risk factors for CKD only; however, previous work has shown this group of people are much less likely to have CKD than those who do have CKD tests recorded in primary care.[22] Furthermore, we included people with at least one reduced kidney function test as potentially having CKD since not every patient undergoes regular CKD testing in our routine clinical datasets. Defining CKD using one eGFR measurement will have led to some misclassification. However, as people with CKD have very high cardiovascular risk and because of the infrequent serum creatinine

For peer review only - http://bmjopen.bmj.com/site/about/guidelines.xhtml

measurement in primary care, applying the chronicity criterion would have led to a selected cohort of people who did not develop a myocardial infarction until the second measurement had been done. Our results are likely impacted by residual confounding, since we were limited in the number of relevant comorbidities we could include in our multivariable models. Finally, AMI misclassification in HES data may have occurred due to the structure and level of detail available in this dataset. For example, we may have missed AMI cases by including only those recorded in the first diagnostic position of the first episode of a HES admission; however, our sensitivity analysis which included AMI hospitalisations recorded in the first or second diagnostic position showed similar results. In addition, unlike MINAP data, HES data do not include ECG results and troponin levels, which we could have used to reduce potential misclassification. Inclusion of the first diagnostic position of later episodes was undertaken in a similar study investigating AMI case ascertainment in people with malignancy, with little improvement in agreement between datasets.[32]

Future research

This study demonstrates how AMI case ascertainment in England can be improved by using linked healthcare datasets. Further research investigating cardiovascular and kidney disease incidence, prevalence and outcomes should follow this approach. Other countries with similarly rich, yet fragmented healthcare datasets would benefit from applying similar methods to evaluate the validity and completeness of cardiovascular and kidney disease capture in similar data. Optimising data quality in healthcare datasets and simplifying the process of data linkage would facilitate high-quality observational research to inform the design of future RCTs and provide estimated treatment effects where RCT data are lacking.

For peer review only - http://bmjopen.bmj.com/site/about/guidelines.xhtml

Conclusion

The use of linked healthcare datasets should be prioritised in observational research investigating multimorbidity.

For peer review only

For peer review only - http://bmjopen.bmj.com/site/about/guidelines.xhtml

ACKNOWLEDGEMENTS

MINAP is commissioned, as part of the 'National Cardiac Audit Project' by the Healthcare Quality Improvement Partnership. This work uses data that has been provided by patients and collected by the NHS as part of their care and support.

We acknowledge and thank UK cardiovascular, renal, and primary care physicians as well as hospital and community audit and coding teams whose diligent data collection has provided the data for these analyses. In particular, we would like to thank James Chal, Anil Gunesh, Andrew Harrison, and Akosua Donkor from NICOR; and Kathryn Griffith, Matthew Harker, Yvonne Silove, Tasneem Hoosain, Nick Wilson, Ronnie Moodley, Richard Fluck, Chris Gush, David Wheeler, Liam Smeeth, Ron Cullen, Andy Syme, Richard Gunn, Paul Wright, Hugh Gallagher, Sion Edwards, Fiona Loud, Nick Palmer, Richard Fluck, Anita Sharma, Kate Cheema, Andy Syme, Sally Hull, Ben Caplin, Lois Kim, and Faye Cleary from the NCKDA steering, clinical reference, and statistical groups.

AUTHOR CONTRIBUTIONS

JS, DMT, DN, DA, FC, and UU conceived the study. JS, DMT, DN, DA, FC, UU, and PB designed the study. PB and DN had access to and analysed the data. PB and JS drafted the manuscript. PB, JS, DMT, UU, FC, LT, MS, JD, MB, SD, CW, DA, DN (all authors) critically appraised the results and edited the manuscript. All authors approved the final version of the manuscript. The lead

For peer review only - http://bmjopen.bmj.com/site/about/guidelines.xhtml

authors confirm all authors meet ICJME authorship criteria, and no one who meets ICJME criteria have been excluded.

COMPETING INTERESTS

All authors have completed an ICJME form. PB, JS, DMT, FC, LT, MDB, and SD have nothing to declare. UU declares a grant from the Health Foundation to undertake quality improvement unrelated to this work. MS declares funding from Cancer Research UK (C53325/A21134) and British Heart Foundation (SP/16/5/32415) for research activities related to this work looking at acute myocardial infarction ascertainment in a cancer population. JS declares Doctoral research funding from the NIHR for related research looking at acute myocardial infarction care for people with chronic kidney disease. JD declares grants from British Heart Foundation paid to his institution unrelated to this work. JD also declares consulting fees from Novo Nordisk, and honoraria from Amgen, Boehringer Ingelheim, Merck, Pfizer, Aegerion, Novartis, Sanofi, Takeda, Novo Nordisk, and Bayer, unrelated to this work. JD is also member of a study steering committee with Novo Nordisk. CW declares he is clinical lead of the Myocardial Ischaemia National Audit Project. DA reports research funding and in-kind support from Astra Zeneca inc. for unrelated research and educational funding from Abbott Vascular inc. to support a clinical research fellow doing unrelated research. DA has conducted consultancy for General Electric inc. to support general research funds. DN is the UK Kidney Association Director of Informatics Research. DN is also on the steering group for two GlaxoSmithKline funded studies that investigate kidney function in children and adults in Sub-Saharan Africa.

For peer review only - http://bmjopen.bmj.com/site/about/guidelines.xhtml

FUNDING

This work was supported by Kidney Research UK (grant number IN_008_20180304) and the Health Foundation (grant number 1725841). JS is a Doctoral Research Fellow funded by the NIHR (NIHR 300906). DA is funded by a joint research grant from the British Heart Foundation (SP/16/5/32415) and Cancer Research UK (C53325/A21134).

DATA SHARING

Due to data sharing agreements, we are not able to share these data. Researchers interested in using these data should consult the websites for the NCKDA (https://www.lshtm.ac.uk/research/centres-projects-groups/ckdaudit#welcome) and MINAP (https://www.nicor.org.uk/national-cardiac-audit-programme/myocardial-ischaemia-minap-heart-attack-audit/).

ETHICS APPROVAL

This study was approved by the London School of Hygiene & Tropical Medicine LEO ethics committee (ID 16988) and the National Chronic Kidney Disease Audit (NCKDA) Steering Committee. Audits used in this study are covered by Section 251 approvals (NHS Act 2006)

For peer review only - http://bmjopen.bmj.com/site/about/guidelines.xhtml

which allows data to be collected without individual patient consent for medical research when it is not possible to use anonymised information and when seeking consent is not practical.

REFERENCES

1. Foundation BH. Heart statistics. Accessed 28/02/2020, 2020. https://www.bhf.org.uk/what-we-do/our-research/heart-statistics

2. Pitt B, Remme W, Zannad F, et al. Eplerenone, a selective aldosterone blocker, in patients with left ventricular dysfunction after myocardial infarction. *The New England journal of medicine*. Apr 3 2003;348(14):1309-21. doi:10.1056/NEJMoa030207

3. Januzzi JL, Cannon CP, DiBattiste PM, Murphy S, Weintraub W, Braunwald E. Effects of renal insufficiency on early invasive management in patients with acute coronary syndromes (The TACTICS-TIMI 18 Trial). *The American journal of cardiology*. Dec 1 2002;90(11):1246-9.

4. Bhatt DL, Roe MT, Peterson ED, et al. Utilization of early invasive management strategies for high-risk patients with non-ST-segment elevation acute coronary syndromes: results from the CRUSADE Quality Improvement Initiative. *Jama*. Nov 3 2004;292(17):2096-104. doi:10.1001/jama.292.17.2096

5. Kedhi E, Fabris E, van der Ent M, et al. Six months versus 12 months dual antiplatelet therapy after drug-eluting stent implantation in ST-elevation myocardial infarction (DAPT-STEMI): randomised, multicentre, non-inferiority trial. *BMJ (Clinical research ed)*. Oct 2 2018;363:k3793. doi:10.1136/bmj.k3793

6. Serruys PW, Kogame N, Katagiri Y, et al. Clinical outcomes of state-of-the-art percutaneous coronary revascularisation in patients with three-vessel disease: two-year follow-up of the SYNTAX II study. *EuroIntervention*. Jun 12 2019;15(3):e244-e252. doi:10.4244/eij-d-18-00980

7. Wright RS, Reeder GS, Herzog CA, et al. Acute myocardial infarction and renal dysfunction: a high-risk combination. *Ann Intern Med*. Oct 1 2002;137(7):563-70.

8. Santopinto JJ, Fox KA, Goldberg RJ, et al. Creatinine clearance and adverse hospital outcomes in patients with acute coronary syndromes: findings from the global registry of acute coronary events (GRACE). *Heart*. Sep 2003;89(9):1003-8. doi:10.1136/heart.89.9.1003

9. Fox CS, Muntner P, Chen AY, et al. Use of evidence-based therapies in short-term outcomes of ST-segment elevation myocardial infarction and non-ST-segment elevation myocardial infarction in patients with chronic kidney disease: a report from the National Cardiovascular Data Acute Coronary Treatment and Intervention Outcomes Network registry. *Circulation*. Jan 26 2010;121(3):357-65. doi:10.1161/circulationaha.109.865352

10. Herzog CA, Ma JZ, Collins AJ. Poor long-term survival after acute myocardial infarction among patients on long-term dialysis. *The New England journal of medicine*. Sep 17 1998;339(12):799-805. doi:10.1056/nejm199809173391203

For peer review only - http://bmjopen.bmj.com/site/about/guidelines.xhtml

11. Konstantinidis I, Nadkarni GN, Yacoub R, et al. Representation of Patients With Kidney Disease in Trials of Cardiovascular Interventions: An Updated Systematic Review. *JAMA internal medicine*. Jan 2016;176(1):121-4. doi:10.1001/jamainternmed.2015.6102
12. Herrett E, Shah AD, Boggon R, et al. Completeness and diagnostic validity of recording acute myocardial infarction events in primary care, hospital care, disease registry, and national mortality records: cohort study. *BMJ (Clinical research ed)*. May 20 2013;346:f2350. doi:10.1136/bmj.f2350
13. Gupta T, Paul N, Kolte D, et al. Association of chronic renal insufficiency with in-hospital outcomes after percutaneous coronary intervention. *J Am Heart Assoc*. Jun 16 2015;4(6):e002069. doi:10.1161/jaha.115.002069
14. Rozenbaum Z, Benchetrit S, Minha S, et al. The Effect of Admission Renal Function on the Treatment and Outcome of Patients with Acute Coronary Syndrome. *Cardiorenal Med*. Jun 2017;7(3):169-178. doi:10.1159/000455239
15. Shaw C, Nitsch D, Steenkamp R, et al. Inpatient coronary angiography and revascularisation following non-ST-elevation acute coronary syndrome in patients with renal impairment: a cohort study using the Myocardial Ischaemia National Audit Project. *PLoS One*. 2014;9(6):e99925. doi:10.1371/journal.pone.0099925
16. Grams ME, Astor BC, Bash LD, Matsushita K, Wang Y, Coresh J. Albuminuria and estimated glomerular filtration rate independently associate with acute kidney injury. *J Am Soc Nephrol*. Oct 2010;21(10):1757-64. doi:10.1681/asn.2010010128
17. Nitsch D CB HS, Wheeler D. National Chronic Kidney Disease Audit - National Report Part 1. 2017;
18. Mudie K CF CB, Wheeler D, Hull S, Nitsch D. National Chronic Kidney Disease Audit: National Report Part 2. 2017;
19. Hospital Episode Statistics. 2020. https://digital.nhs.uk/data-and-information/data-tools-and-services/data-services/hospital-episode-statistics
20. Thygesen K, Alpert JS, Jaffe AS, et al. Fourth universal definition of myocardial infarction (2018). *Eur Heart J*. Aug 25 2018;doi:10.1093/eurheartj/ehy462
21. audit) MIMHA. https://www.nicor.org.uk/national-cardiac-audit-programme/myocardial-ischaemia-minap-heart-attack-audit/,
22. Iwagami M, Tomlinson LA, Mansfield KE, et al. Validity of estimated prevalence of decreased kidney function and renal replacement therapy from primary care electronic health records compared with national survey and registry data in the United Kingdom. *Nephrol Dial Transplant*. Apr 1 2017;32(suppl_2):ii142-ii150. doi:10.1093/ndt/gfw318
23. Levey AS, Coresh J, Greene T, et al. Using standardized serum creatinine values in the modification of diet in renal disease study equation for estimating glomerular filtration rate. *Ann Intern Med*. Aug 15 2006;145(4):247-54. doi:10.7326/0003-4819-145-4-200608150-00004
24. Levey AS, Stevens LA, Schmid CH, et al. A new equation to estimate glomerular filtration rate. *Ann Intern Med*. May 5 2009;150(9):604-12. doi:10.7326/0003-4819-150-9-200905050-00006
25. Stevens PE, Levin A. Evaluation and management of chronic kidney disease: synopsis of the kidney disease: improving global outcomes 2012 clinical practice guideline. *Ann Intern Med*. Jun 4 2013;158(11):825-30. doi:10.7326/0003-4819-158-11-201306040-00007

For peer review only - http://bmjopen.bmj.com/site/about/guidelines.xhtml

26. Herbert A, Wijlaars L, Zylbersztejn A, Cromwell D, Hardelid P. Data Resource Profile: Hospital Episode Statistics Admitted Patient Care (HES APC). *Int J Epidemiol*. Aug 1 2017;46(4):1093-1093i. doi:10.1093/ije/dyx015
27. Schnier C BK NJ, Sudlow C. Definitions of Acute Myocardial Infarction and Main Myocardial Infarction Pathological Types - UK Biobank Phase 1 Outcomes Adjudication. Accessed 12 January, 2021. https://biobank.ndph.ox.ac.uk/showcase/showcase/docs/alg_outcome_mi.pdf.
28. Giavarina D. Understanding Bland Altman analysis. *Biochem Med (Zagreb)*. 2015;25(2):141-51. doi:10.11613/bm.2015.015
29. Torabi A, Cleland JG, Sherwi N, et al. Influence of case definition on incidence and outcome of acute coronary syndromes. *Open Heart*. 2016;3(2):e000487. doi:10.1136/openhrt-2016-000487
30. Baron T, Hambraeus K, Sundström J, Erlinge D, Jernberg T, Lindahl B. Type 2 myocardial infarction in clinical practice. *Heart*. Jan 2015;101(2):101-6. doi:10.1136/heartjnl-2014-306093
31. Pickering JW, Blunt IRH, Than MP. Acute Kidney Injury and mortality prognosis in Acute Coronary Syndrome patients: A meta-analysis. *Nephrology*. 2018;23(3):237-246. doi:https://doi.org/10.1111/nep.12984
32. Coles B, Teece L, Weston C, et al. Case-ascertainment of acute myocardial infarction hospitalizations in cancer patients: a cohort study using English linked electronic health data. *European Heart Journal - Quality of Care and Clinical Outcomes*. 2021;doi:10.1093/ehjqcco/qcab045

For peer review only

For peer review only - http://bmjopen.bmj.com/site/about/guidelines.xhtml

FIGURE LEGEND

Figure 1: Venn diagrams illustrating AMI recording in MINAP and HES secondary care datasets. Venn diagrams presented overall, and stratified by CKD status (at-risk of or mild CKD, eGFR≥60mL/min/1.73m^2 or moderate to severe CKD, eGFR<60 mL/min/1.73m^2). Circle areas are proportional to the number of AMI events in each dataset.

TABLE LEGEND

Table 1: Baseline characteristics by CKD stage for all AMI events captured after the study start. n (column %) unless specified otherwise.

Table 2: Multinomial logistic regression comparing the relative risk ratios (RRR) of AMI recording across HES and MINAP according to CKD stage. The comparator outcome is people with AMI recorded in both HES and MINAP databases.

Table 3: Death during and after first AMI hospitalisation in total study population at risk of or with CKD.

Table 4: Agreement between primary care-derived CKD stage (NCKDA) and secondary care-derived eGFR stage (MINAP)

For peer review only

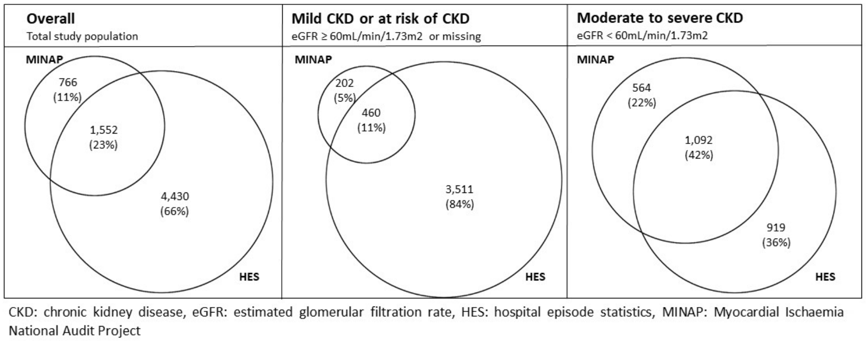

Figure 1: Venn diagrams illustrating AMI recording in MINAP and HES secondary care datasets. Venn diagrams presented overall, and stratified by CKD status (at-risk of or mild CKD, eGFR≥60mL/min/1.73m2 or moderate to severe CKD, eGFR<60 mL/min/1.73m2). Circle areas are proportional to the number of AMI events in each dataset.

338x190mm (96 x 96 DPI)

Supplementary materials

Supplementary tables

Supplementary table 1: ICD-10 codes for AMI identified in HES

Supplementary table 2: CALIBER definition of AMI subtypes (STEMI, NSTEMI) using MINAP data.

Supplementary table 3: Variables used to define death in-hospital and post-AMI discharge.

Supplementary table 4: Details on data sources for covariates.

Supplementary table 5: AMI database capture by CKD status (binary) stratified by AMI subtype (STEMI or NSTEMI)

Supplementary table 6: Serum creatinine (SCr) measured in the first 24 hours of AMI hospitalization recorded in MINAP, stratified by CKD stage defined in primary care (NCKDA)

Supplementary table 7: AMI hospitalisations occurring before study start – description of people in the NCKDA who experienced AMI hospitalisation recorded in MINAP or HES before the study start. n (column %) unless specified otherwise.

Supplementary table 8: AMI hospitalisations occurring before study start - multinomial logistic regression comparing the relative risk ratios (RRR) of AMI recording across HES and MINAP according to CKD stage. The comparator outcome is people with AMI recorded in both HES and MINAP databases.

Supplementary table 9: AMI hospitalisations occurring before study start - agreement between primary care-derived CKD stage (NCKDA) and secondary care-derived CKD stage (MINAP)

Supplementary table 10: Multinomial logistic regression comparing the relative risk ratios (RRR) of AMI recording across HES and MINAP according to CKD stage, after excluding those with a history of dialysis. The comparator outcome is people with AMI recorded in both HES and MINAP databases.

Supplementary table 11: Death during and after first AMI hospitalisation in study population at risk of or with CKD, excluding those with a history of dialysis.

Supplementary table 12: Sensitivity analysis describing the ICD-10 diagnoses (first episode, first diagnostic position) of non-AMI HES cases that are within ±30 days of MINAP only AMI cases from the main analyses

Supplementary figures

Supplementary figure 1: Flow diagram illustrating the NCKDA extracts, and resulting contribution of AMI events to this study

Supplementary figure 2: AMI case ascertainment in MINAP and HES datasets over time.

Supplementary figure 3: Bland-Altman plot comparing the mean eGFR (NCKDA eGFR and MINAP eGFR, x-axis) and the difference between the NCKDA eGFR and the MINAP eGFR (y-axis).

Supplementary figure 4: AMI hospitalisations occurring before study start - comparing AMI case ascertainment overall and by CKD status for AMI hospitalisations in the main analysis and AMI hospitalisations which occurred prior to the last NCKDA extract (sensitivity analysis).

Supplementary figure 5: Sensitivity analyses compared to main analysis, describing AMI recording in MINAP and HES in the overall study population at risk of or with CKD. Areas of Venn diagrams are proportional to the number of cases.

Supplementary table 1: ICD-10 codes for AMI identified in HES

**AMI subtype**	**ICD-10 codes**
Overall	I21, I22, I23
STEMI	I21.0, I21.1, I21.2, I21.3, I22.0, I22.1, I22.8
NSTEMI	I21.4, I21.9, I22.9
Missing	I23

AMI: acute myocardial infarction; HES: Hospital Episode Statistics; ICD-10: International Classification of Diseases 10th Edition; STEMI: ST elevated myocardial infarction; NSTEMI: Non-ST elevated myocardial infarction

Supplementary table 2: CALIBER definition of AMI subtypes (STEMI, NSTEMI) using MINAP data.

	MINAP variable		
**AMI subtype**	**Discharge diagnosis**	**Markers elevated?**	**ECG result**
Other	Threatened MI, Chest pain uncertain cause, MI unconfirmed, other diagnosis	-	-
STEMI	STEMI	Raised or missing	ST elevation, LBBB, or ST elevation
	NSTEMI/Troponin positive ACS	Raised or missing	ST elevation
	ACS troponin negative	Raised	ST elevation
	ACS troponin unspecified	Raised	ST elevation
NSTEMI	NSTEMI/Troponin positive ACS	Raised or missing	ST depression, T wave changes only, other abnormality, Normal ECG, or LBBB
	ACS troponin negative	Raised	LBBB, ST depression, T wave changes only, Other abnormality, normal ECG, or missing
	ACS troponin unspecified	Raised	LBBB, ST depression, T wave changes only, Other abnormality, normal ECG, or missing
Unstable angina	*Any remaining hospitalisations not assigned as STEMI, NSTEMI, or other diagnosis		

ACS: acute coronary syndrome, AMI: acute myocardial infarction, ECG: electrocardiogram, LBBB: left bundle branch block, MI: myocardial infarction, MINAP: Myocardial Ischaemia National Audit Project, NSTEMI: Non ST-elevation myocardial infarction, STEMI: ST-elevation myocardial infarction

Supplementary table 3: Variables used to define death in-hospital and post-AMI discharge.

**Data source**	**Variables to define in-hospital death**	**Variables to define death post-AMI discharge (in those who survive first AMI hospitalization)**
ONS	Date of death (falling on or in between first AMI admission and discharge dates)	Date of death (falling after first AMI discharge date)
MINAP	Reason for no angiography, no intervention, admission ward, discharge destination	-
HES	Discharge location, discharge method	-

AMI: acute myocardial infarction, HES: Hospital Episode Statistics, MINAP: Myocardial Ischaemia National Audit Project, ONS: Office of National Statistics

Supplementary table 4: Details on data sources for covariates.

Category	Covariate	Data source
Sociodemographic and lifestyle variables	Age at AMI admission	NCKDA
	Sex	NCKDA
	IMD quintiles	NCKDA
	Smoking status	NCKDA, MINAP
Comorbidities	Angina	MINAP and HES
	Cerebrovascular disease	MINAP and HES
	COPD	MINAP and HES
	Diabetes mellitus	MINAP and HES
	Heart failure	MINAP and HES
	Hypertension	NCKDA, MINAP, and HES
	Previous myocardial infarction	MINAP, and HES
	Peripheral vascular disease	NCKDA, MINAP, and HES
	Dialysis	NCKDA
	Kidney transplant	NCKDA

AMI: Acute Myocardial Infarction, COPD: Chronic Obstructive Pulmonary Disease, HES: Hospital Episode Statistics, IMD: Index of Multiple Deprivation, NCKDA: National Chronic Kidney Disease Audit, MINAP: Myocardial Ischaemia National Audit Project

Supplementary table 5: AMI database capture by CKD status (binary) stratified by AMI subtype (STEMI or NSTEMI)

AMI subtype	Database (outcome, compared to people with AMI recorded in MINAP & HES)	CKD stage (exposure)	Number of AMI admissions	Unadjusted RRR (95% CI)	Adjusted RRR[1] (95% CI)
**STEMI**	**MINAP only**	At risk of CKD & Stages 1-2	31	1	1
	(N=115)	Stage 3a-5	74	1.08 (0.68-1.72)	0.88 (0.52-1.49)
	**HES only**	At risk of CKD & Stages 1-2	1,306	1	1
	(N=1,482)	Stage 3a-5	194	0.07 (0.05-0.09)	0.07 (0.05-0.09)
**NSTEMI**	**MINAP only**	At risk of CKD & Stages 1-2	167	1	1
	(N=666)	Stage 3a-5	474	1.16 (0.93-1.45)	1.11 (0.88-1.41)
	**HES only**	At risk of CKD & Stages 1-2	2,164	1	1
	(N=2,834)	Stage 3a-5	717	0.14 (0.12-0.16)	0.15 (0.12-0.17)

[1] adjusted for sex, age group at AMI admission, ethnicity, previous AMI, and clustering by patient

AMI: Acute Myocardial Infarction, CKD: chronic kidney disease, HES: Hospital Episode Statistics, MINAP: Myocardial Ischaemia National Audit Project, NSTEMI: non-ST elevation myocardial infarction, RRR: relative risk ratio, STEMI: ST-elevation myocardial infarction

Supplementary table 6: Serum creatinine (SCr) measured in the first 24 hours of AMI hospitalization recorded in MINAP, stratified by CKD stage defined in primary care (NCKDA)

CKD status (NCKDA)	SCr recorded in MINAP at AMI admission, n (%)	MINAP median SCr, umol (IQR)	MINAP Median SCr value*, mg/dL (IQR)	MINAP Median eGFR, mL/min/1.73m^2 (IQR)
All stages	2,240 (97)	115 (91-152)	1.30 (1.03-1.72)	47.6 (33.5-61.6)
At risk of CKD & Stages 1-2	630 (97)	88 (74-104)	1.00 (0.84-1.18)	67.0 (56.0-80.2)
3a	770 (97)	109 (94-128)	1.23 (1.06-1.45)	50.8 (41.9-59.2)
3b	587 (98)	140 (117-174)	1.58 (1.32-1.97)	36.7 (29.4-43.4)
4-5	253 (95)	255 (190-407)	2.88 (2.15-4.60)	19.8 (12.0-25.5)

*Multiplied by 0.95 for SCr taken before 2013

AMI: acute myocardial infarction, CKD: chronic kidney disease, eGFR: estimated glomerular filtration rate, NCKDA: National Chronic Kidney Disease Audit, MINAP: Myocardial Ischaemia National Audit Project, SCr: serum creatinine

Supplementary table 7: AMI hospitalisations occurring before study start – description of people in the NCKDA who experienced AMI hospitalisation recorded in MINAP or HES before the study start. n (column %) unless specified otherwise.

**CKD status** (main exposure)	**No CKD**	**CKD**			**Total**
**CKD stage** (secondary exposure)	**At-risk of CKD & 1-2**	**3a**	**3b**	**4-5**	
**Unique individuals**	27,909	4,884	2,083	726	34,860
**Total number of AMI events, N**	32,041	5,641	2,460	926	41,068
**Age at AMI event, years, mean (SD)**	64 (13)	75 (10)	78 (10)	75 (12)	66 (13)
**Age category at AMI event, years**					
18-50	4,506 (14)	83 (1)	22 (1)	34 (4)	4,645 (11)
50-64	11,964 (37)	829 (15)	207 (8)	130 (14)	13,130 (32)
65-79	11,517 (36)	2,777 (49)	986 (40)	405 (44)	15,685 (38)
80+	3,922 (12)	1,952 (35)	1,245 (51)	357 (39)	7,476 (18)
Missing	132 (0)	0 (0)	0 (0)	0 (0)	132 (0)
**Female**	9,288 (29)	2,482 (44)	1,209 (49)	394 (43)	13,373 (33)
**Ethnicity**					
White	29,016 (91)	5,228 (93)	2,257 (92)	846 (91)	37,347 (91)
Other	2,612 (8)	295 (5)	139 (6)	71 (8)	3,117 (8)
Missing	413 (1)	118 (2)	64 (3)	9 (1)	604 (1)
**IMD quintile**					
1 (least deprived)	5,808 (18)	1,121 (20)	456 (19)	184 (20)	7,569 (18)
2	6,716 (21)	1,284 (23)	523 (21)	182 (20)	8,705 (21)
3	6,922 (22)	1,297 (23)	542 (22)	180 (19)	8,941 (22)
4	6,820 (21)	1,134 (20)	525 (21)	216 (23)	8,695 (21)
5 (most deprived)	5,531 (17)	787 (14)	406 (17)	161 (17)	6,885 (17)
Missing	244 (1)	18 (0)	8 (0)	3 (0)	273 (1)
**Dialysis in primary care**					
Peritoneal dialysis	0 (0)	0 (0)	0 (0)	12 (1)	12 (0)
Haemodialysis	0 (0)	0 (0)	0 (0)	24 (3)	24 (0)
Renal dialysis, unspecified	0 (0)	0 (0)	0 (0)	11 (1)	11 (0)
**Kidney transplant**	3 (0)	4 (0)	8 (0)	11 (1)	26 (0)
**Comorbidities**					
Angina	5,001 (16)	1,630 (29)	882 (36)	335 (36)	7,848 (19)
Cerebrovascular disease	1,405 (4)	497 (9)	309 (13)	135 (15)	2,346 (6)

CKD status (main exposure)	No CKD	CKD			Total
**CKD stage (secondary exposure)**	**At-risk of CKD & 1-2**	**3a**	**3b**	**4-5**	
**Unique individuals**	27,909	4,884	2,083	726	34,860
**Total number of AMI events, N**	32,041	5,641	2,460	926	41,068
COPD	2,059 (6)	680 (12)	368 (15)	147 (16)	3,254 (8)
Diabetes	5,823 (18)	1,500 (27)	873 (35)	463 (50)	8,659 (21)
Heart failure	1,228 (4)	456 (8)	353 (14)	199 (21)	2,236 (5)
Hypertension	10,556 (33)	3,287 (58)	1,721 (70)	673 (73)	16,237 (40)
Myocardial infarction	5,563 (17)	1,510 (27)	832 (34)	380 (41)	8,285 (20)
Peripheral vascular disease	799 (2)	258 (5)	181 (7)	106 (11)	1,344 (3)
**Smoking status**					
non-smoker	12,945 (40)	2,049 (36)	835 (34)	301 (33)	16,130 (39)
ever-smoker	16,626 (52)	2,477 (44)	983 (40)	382 (41)	20,468 (50)
Missing	2,470 (8)	1,115 (20)	642 (26)	243 (26)	4,470 (11)

AMI: Acute Myocardial Infarction, CKD: chronic kidney disease, COPD: Chronic Obstructive Pulmonary Disease, HES: Hospital Episode Statistics, IMD: Index of Multiple Deprivation, NCKDA: National Chronic Kidney Disease Audit, MINAP: Myocardial Ischaemia National Audit Project

Supplementary table 8: AMI hospitalisations occurring before study start - multinomial logistic regression comparing the relative risk ratios (RRR) of AMI recording across HES and MINAP according to CKD stage. The comparator outcome is people with AMI recorded in both HES and MINAP databases.

**AMI recording** (outcome, compared to people with AMI recorded in MINAP & HES)	**CKD stage** (exposure)	**Number of AMI admissions, n=**	**Unadjusted[1] RRR (95% CI)**	**Partially adjusted[2] RRR (95% CI)**	**Adjusted[3] RRR (95% CI)**
**MINAP only** (N=3,957)	At-risk/Stages 1-2	2,147	1	1	1
	Stage 3a	999	0.96 (0.87-1.05)	0.90 (0.82-0.99)	0.88 (0.80-0.97)
	Stage 3b	578	1.22 (1.09-1.36)	1.12 (0.99-1.26)	1.05 (0.93-1.19)
	Stages 4-5	233	1.35 (1.14-1.60)	1.27 (1.07-1.51)	1.13 (0.94-1.35)
**HES only** (N=26,697)	At-risk/Stages 1-2	23,904	1	1	1
	Stage 3a	1,911	0.16 (0.15-0.18)	0.23 (0.22-0.25)	0.24 (0.22-0.25)
	Stage 3b	630	0.12 (0.11-0.13)	0.20 (0.17-0.22)	0.21 (0.18-0.23)
	Stages 4-5	252	0.13 (0.11-0.15)	0.18 (0.15-0.22)	0.20 (0.17-0.24)

[1]Complete cases for adjusted model
[2]Adjusted for sex, age at AMI admission, ethnicity (white, other), IMD quintile, clustering by participant
[3]Additionally adjusted for comorbidities previous AMI, heart failure, COPD, diabetes mellitus

AMI: Acute Myocardial Infarction, CKD: chronic kidney disease, COPD: Chronic Obstructive Pulmonary Disease, HES: Hospital Episode Statistics, IMD: Index of Multiple Deprivation, MINAP: Myocardial Ischaemia National Audit Project, RRR: relative risk ratio

Supplementary table 9: AMI hospitalisations occurring before study start - agreement between primary care-derived CKD stage (NCKDA) and secondary care-derived CKD stage (MINAP)

	% agreement	Kappa statistic (SE)
**Overall[1]**	66.4%	0.51 (0.007)
**CKD stages 3a, 3b, 4-5[2]**	57.8%	0.37 (0.008)
**CKD stages 1-2, 3a-5[3]**	80.3%	0.59 (0.010)
**Overall[1], by time from NCKDA SCr test (primary care) to MINAP SCr test (at AMI secondary care admission)**		
0-5 months	66.7%	0.52 (0.009)
6-11 months	65.6%	0.49 (0.013)
12-23 months	63.3%	0.42 (0.023)
24-36 months	65.0%	0.45 (0.034)

[1]Overall agreement when grouping as (1) Stages 1-2 (eGFR 60-120mL/min/1.73m^2), (2) Stage 3a (eGFR 45-59mL/min/1.73m^2), (3) Stage 3b (eGFR 30-44 mL/min/1.73m^2), (4) Stages 4-5 (eGFR 0-30 mL/min/1.73m^2)
[2]Agreement when restricting to people with CKD stages 3a-5, grouped as (1) Stage 3a (eGFR 45-59mL/min/1.73m^2), (2) Stage 3b (eGFR 30-44 mL/min/1.73m^2), (3) Stages 4-5 (eGFR 0-30 mL/min/1.73m^2)
[3]Agreement when grouping as (1) Stages 1-2 (eGFR 60-120mL/min/1.73m^2) and (2) Stages 3a-5 (eGFR 0-59mL/min/1.73m^2)

AMI: Acute Myocardial Infarction, CKD: chronic kidney disease, NCKDA: National Chronic Kidney Disease Audit, MINAP: Myocardial Ischaemia National Audit Project, SCr: serum creatinine

Supplementary table 10: Multinomial logistic regression comparing the relative risk ratios (RRR) of AMI recording across HES and MINAP according to CKD stage, after excluding those with a history of dialysis. The comparator outcome is people with AMI recorded in both HES and MINAP databases.

**AMI recording** (outcome, compared to people with AMI recorded in MINAP & HES)	**CKD stage** (exposure)	**Number of AMI admissions, n=**	**Unadjusted[1] RRR (95% CI)**	**Partially adjusted[2] RRR (95% CI)**	**Adjusted[3] RRR (95% CI)**
**MINAP only** (N=731)	At-risk/Stages 1-2	196	1	1	1
	Stage 3a	245	1.07 (0.85-1.34)	0.98 (0.77-1.25)	0.98 (0.77-1.25)
	Stage 3b	197	1.17 (0.92-1.49)	1.04 (0.80-1.36)	1.03 (0.79-1.35)
	Stages 4-5	93	1.54 (1.14-1.60)	1.41 (1.01-1.97)	1.36 (0.97-1.92)
**HES only** (N=4,353)	At-risk/Stages 1-2	3,456	1	1	1
	Stage 3a	557	0.14 (0.12-0.16)	0.14 (0.12-0.17)	0.14 (0.12-0.16)
	Stage 3b	224	0.08 (0.06-0.09)	0.08 (0.07-0.10)	0.08 (0.06-0.10)
	Stages 4-5	116	0.11 (0.08-0.14)	0.12 (0.09-0.15)	0.12 (0.09-0.16)

[1]Complete cases for adjusted model

[2]Adjusted for sex, age at AMI admission, ethnicity (white, other), IMD quintile, clustering by participant

[3]Additionally adjusted for comorbidities previous AMI, heart failure, COPD, diabetes mellitus

AMI: Acute Myocardial Infarction, CKD: chronic kidney disease, COPD: Chronic Obstructive Pulmonary Disease, HES: Hospital Episode Statistics, IMD: Index of Multiple Deprivation, MINAP: Myocardial Ischaemia National Audit Project, RRR: relative risk ratio

Supplementary table 11: Death during and after first AMI hospitalisation in study population at risk of or with CKD, excluding those with a history of dialysis.

**Death during first AMI hospitalisation (N=5,919)[1]**	**Number who died, n (%)**	-	**Unadjusted OR (95% CI)**	**Adjusted[2] OR (95% CI)**
MINAP & HES	208 (15)	-	1	1
MINAP only	148 (23)	-	1.65 (1.31-2.09)	1.59 (1.25-2.03)
HES only	575 (15)	-	0.97 (0.82-1.15)	1.61 (1.32-1.96)
**Death during complete follow-up in those who survive first AMI hospitalisation (N=5,009)[1]**	**Number who died during follow-up, n**	**Rate per 100 person-years (95% CI)**	**Unadjusted HR (95% CI)**	**Adjusted[2] HR (95% CI)**
MINAP & HES	445	17.5 (16.0-19.2)	1	1
MINAP only	234	23.4 (20.6-26.6)	1.30 (1.11-1.52)	1.14 (0.97-1.34)
HES only	841	10.3 (9.58-11.0)	0.61 (0.54-0.68)	1.09 (0.96-1.24)

[1]Complete cases for adjusted model

[2]Adjusted for sex, age at AMI admission, ethnicity (white, other), IMD quintile, previous AMI, heart failure, COPD, diabetes mellitus

AMI: Acute Myocardial Infarction, CKD: chronic kidney disease, COPD: Chronic Obstructive Pulmonary Disease, HES: Hospital Episode Statistics, HR: hazard ratio, IMD: Index of Multiple Deprivation, NCKDA: National Chronic Kidney Disease Audit, MINAP: Myocardial Ischaemia National Audit Project, OR: odds ratio

Supplementary table 12: Sensitivity analysis describing the ICD-10 diagnoses (first episode, first diagnostic position) of non-AMI HES cases that are within ±30 days of MINAP only AMI cases from the main analyses

**Main analysis**		
**MINAP only AMI hospitalisations matched with non-AMI HES hospitalisations**		677/766 (88%)
**Short ICD-10 code (1st episode, 1st diagnostic position)**		**n (% of matched)**
I24	Other acute ischaemic heart diseases	114 (17)
I25	Chronic ischaemic heart disease	84 (12)
R07	Pain in throat and chest	63 (9)
J18	Pneumonia, organism unspecified	56 (8)
I20	Angina pectoris	51 (8)
I50	Heart failure	39 (6)
S72	Fracture of femur	18 (3)
N39	Other disorders of urinary system	14 (2)
A41	Other sepsis	11 (2)
R06	Abnormalities of breathing	11 (2)
**Sensitivity analysis – AMI hospitalisations occurring before study start**		
**MINAP only AMI hospitalisations matched with non-AMI HES hospitalisations**		3,908/4,470 (87%)
**Short ICD-10 code (1st episode, 1st diagnostic position)**		**n (% of matched)**
I25	Chronic ischaemic heart disease	996 (25)
I20	Angina pectoris	760 (19)
R07	Pain in throat and chest	588 (15)
I24	Other acute ischaemic heart diseases	271 (7)
I50	Heart failure	129 (3)

J18	Pneumonia, organism unspecified	86 (2)
I48	Atrial fibrillation and flutter	73 (2)
R06	Abnormalities of breathing	49 (1)
I46	Cardiac arrest	42 (1)
S72	Fracture of femur	40 (1)

AMI: Acute Myocardial Infarction, HES: Hospital Episode Statistics, ICD-10: International Classification of Diseases – 10th Edition, MINAP: Myocardial Ischaemia National Audit Project

Supplementary figure 1: Flow diagram illustrating the NCKDA extracts, and resulting contribution of AMI events to this study

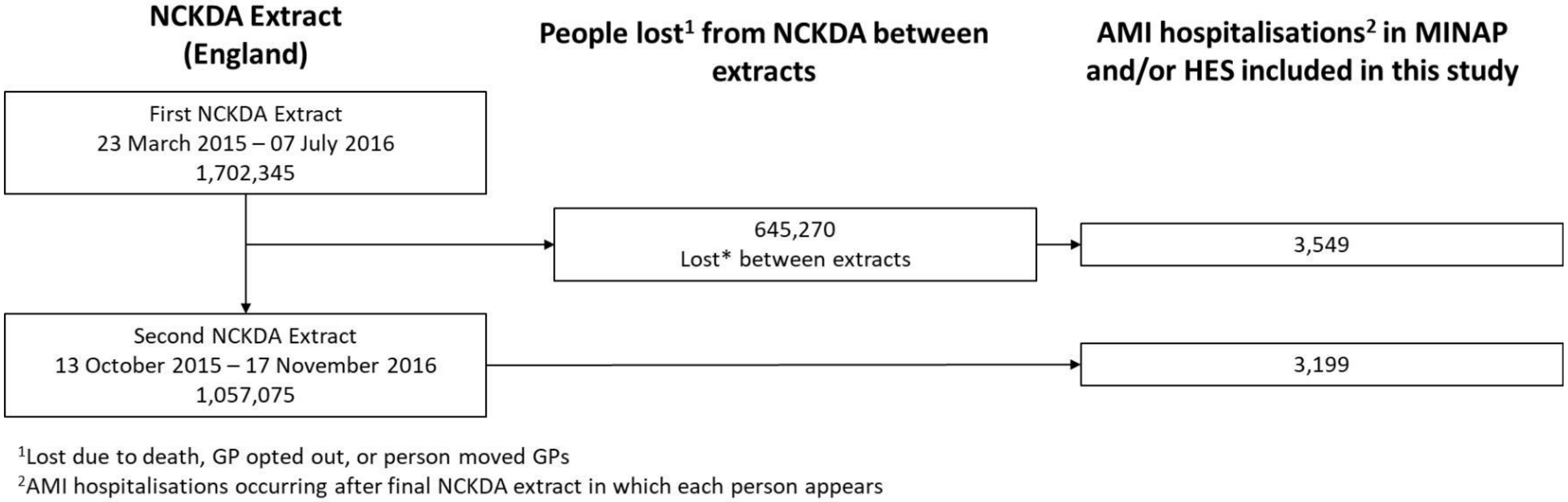

AMI: acute myocardial infarction, GP: General Practice, NCKDA: National Chronic Kidney Disease Audit, MINAP: Myocardial Ischaemia National Audit Project

Supplementary figure 2: AMI case ascertainment in MINAP and HES datasets over time.

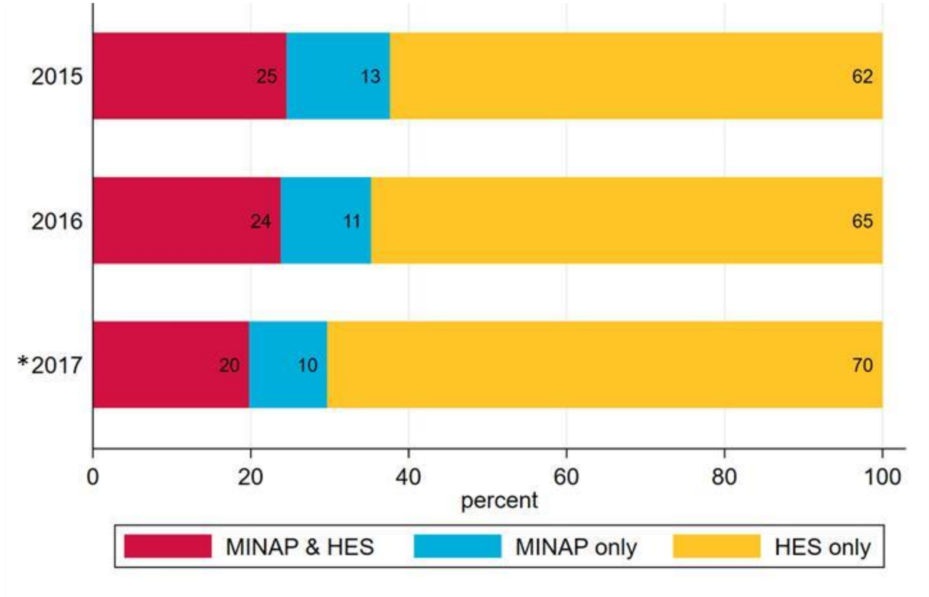

*2017 includes AMI hospitalisations up to 31 March only

AMI: acute myocardial infarction, HES: Hospital Episode Statistics, MINAP: Myocardial Ischaemia National Audit Project

Supplementary figure 3: Bland-Altman plot comparing the mean eGFR (NCKDA eGFR and MINAP eGFR, x-axis) and the difference between the NCKDA eGFR and the MINAP eGFR (y-axis).

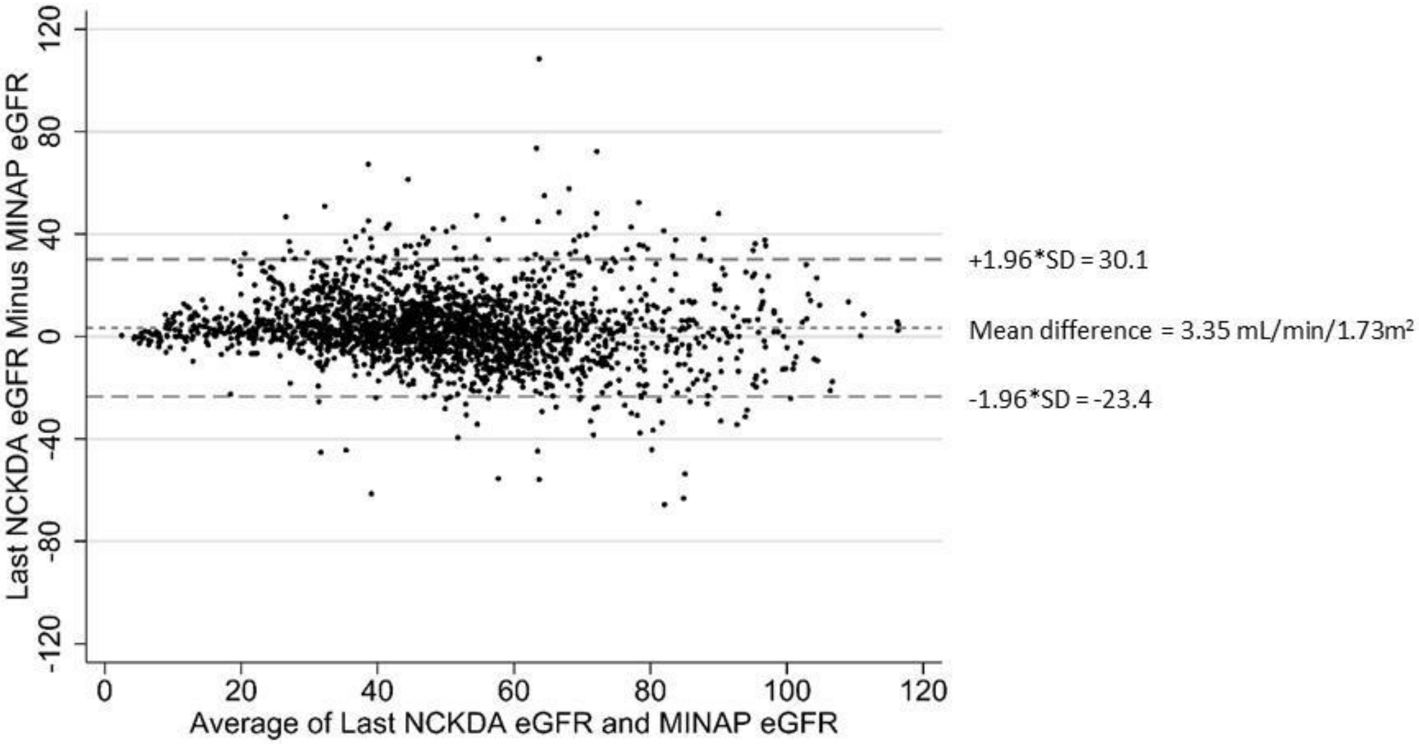

eGFR: estimated glomerular filtration rate, NCKDA: National Chronic Kidney Disease Audit, MINAP: Myocardial Ischaemia National Audit Project

Supplementary figure 4: AMI hospitalisations occurring before study start - comparing AMI case ascertainment overall and by CKD status for AMI hospitalisations in the main analysis and AMI hospitalisations which occurred prior to the last NCKDA extract (sensitivity analysis).

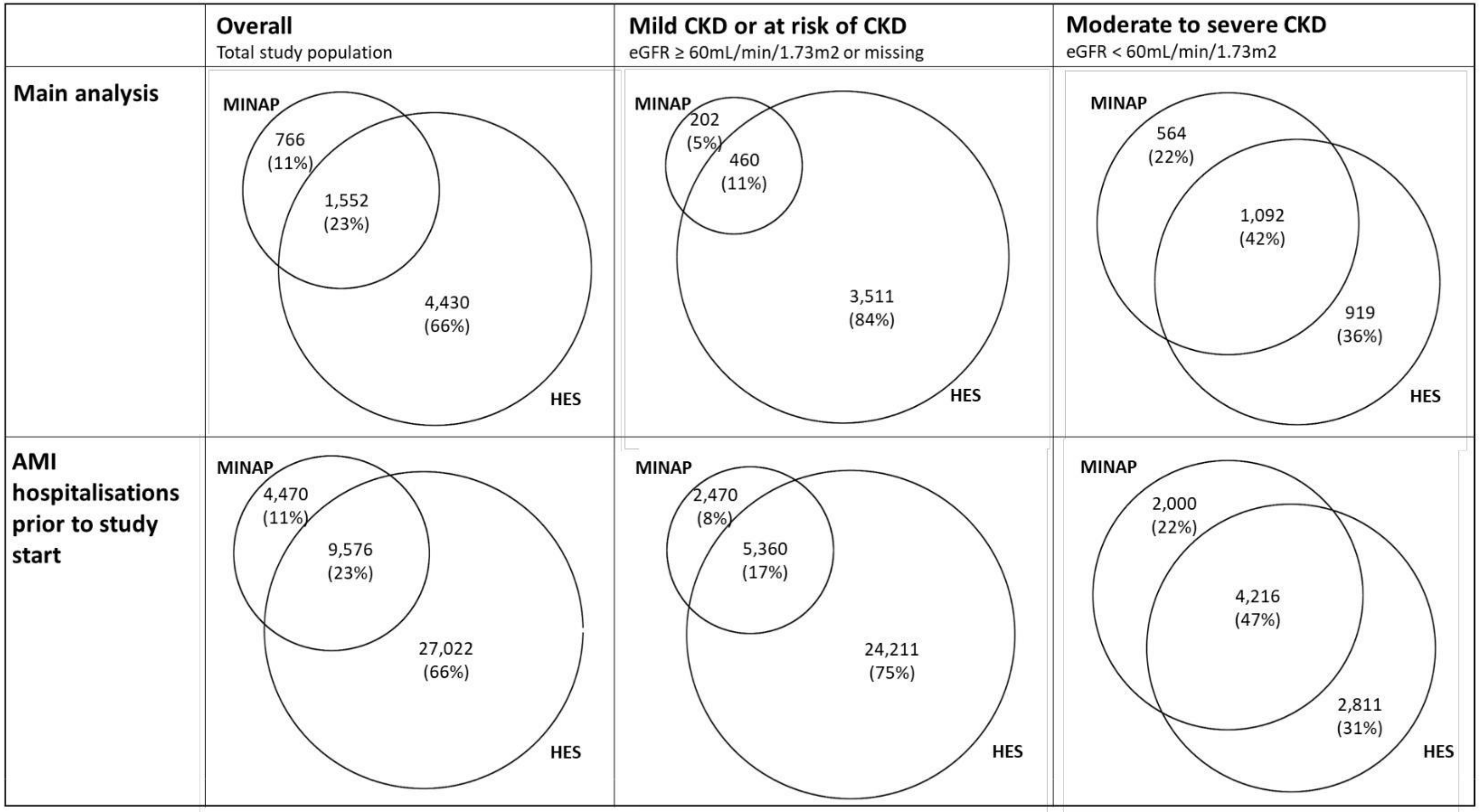

AMI: Acute Myocardial Infarction, CKD: chronic kidney disease, eGFR: estimated glomerular filtration rate, NCKDA: National Chronic Kidney Disease Audit, MINAP: Myocardial Ischaemia National Audit Project

Supplementary figure 5: Sensitivity analyses compared to main analysis, describing AMI recording in MINAP and HES in the overall study population at risk of or with CKD. Areas of Venn diagrams are proportional to the number of cases.

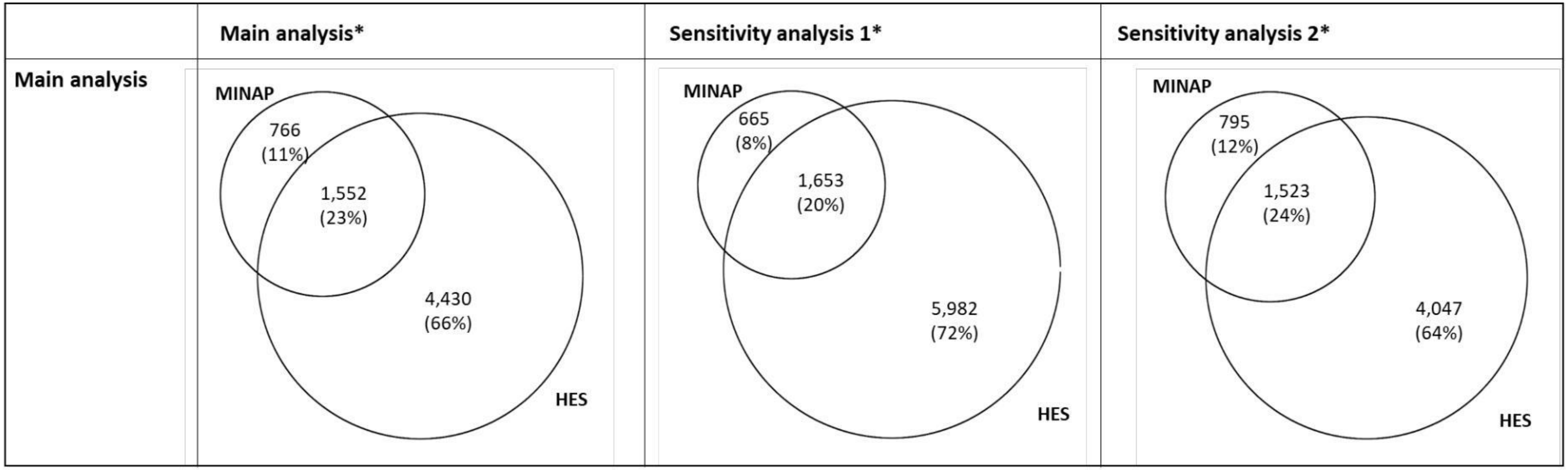

***Main analysis:** HES AMI cases defined as AMI recorded in 1st diagnostic position, 1st episode of hospitalisation
***Sensitivity analysis 1:** HES AMI cases defined as AMI recorded in 1st or 2nd diagnostic position, 1st episode of hospitalisation
***Sensitivity analysis 2:** HES AMI cases in 1st diagnostic position, 1st episode of hospitalisation (as per main analysis) within 30 days of each other counted as a single AMI case, then matched to MINAP AMI cases

AMI: Acute Myocardial Infarction, CKD: chronic kidney disease, HES: Hospital Episode Statistics, MINAP: Myocardial Ischaemia National Audit Project

STROBE 2007 (v4) Statement—Checklist of items that should be included in reports of *cohort studies*

Section/Topic	Item #	Recommendation	Reported on page #
**Title and abstract**	1	(*a*) Indicate the study's design with a commonly used term in the title or the abstract	1
		(*b*) Provide in the abstract an informative and balanced summary of what was done and what was found	4-5
**Introduction**			
Background/rationale	2	Explain the scientific background and rationale for the investigation being reported	7-8
Objectives	3	State specific objectives, including any prespecified hypotheses	8
**Methods**			
Study design	4	Present key elements of study design early in the paper	8-10
Setting	5	Describe the setting, locations, and relevant dates, including periods of recruitment, exposure, follow-up, and data collection	8-9
Participants	6	(*a*) Give the eligibility criteria, and the sources and methods of selection of participants. Describe methods of follow-up	9-10
		(*b*) For matched studies, give matching criteria and number of exposed and unexposed	N/a
Variables	7	Clearly define all outcomes, exposures, predictors, potential confounders, and effect modifiers. Give diagnostic criteria, if applicable	10-13
Data sources/ measurement	8*	For each variable of interest, give sources of data and details of methods of assessment (measurement). Describe comparability of assessment methods if there is more than one group	12-13
Bias	9	Describe any efforts to address potential sources of bias	13-15, 21-22
Study size	10	Explain how the study size was arrived at	8-9
Quantitative variables	11	Explain how quantitative variables were handled in the analyses. If applicable, describe which groupings were chosen and why	12
Statistical methods	12	(*a*) Describe all statistical methods, including those used to control for confounding	13-16
		(*b*) Describe any methods used to examine subgroups and interactions	13-16
		(*c*) Explain how missing data were addressed	15-16
		(*d*) If applicable, explain how loss to follow-up was addressed	14
		(*e*) Describe any sensitivity analyses	15
**Results**			

For peer review only

Participants	13*	(a) Report numbers of individuals at each stage of study—eg numbers potentially eligible, examined for eligibility, confirmed eligible, included in the study, completing follow-up, and analysed	17
		(b) Give reasons for non-participation at each stage	N/a
		(c) Consider use of a flow diagram	Supp figure 1
Descriptive data	14*	(a) Give characteristics of study participants (eg demographic, clinical, social) and information on exposures and potential confounders	17, Table 1
		(b) Indicate number of participants with missing data for each variable of interest	Table 1
		(c) Summarise follow-up time (eg, average and total amount)	18, supp figure 1
Outcome data	15*	Report numbers of outcome events or summary measures over time	17-18
Main results	16	(*a*) Give unadjusted estimates and, if applicable, confounder-adjusted estimates and their precision (eg, 95% confidence interval). Make clear which confounders were adjusted for and why they were included	17-18, Table 2
		(*b*) Report category boundaries when continuous variables were categorized	Table 2
		(*c*) If relevant, consider translating estimates of relative risk into absolute risk for a meaningful time period	N/a
Other analyses	17	Report other analyses done—eg analyses of subgroups and interactions, and sensitivity analyses	18-20
**Discussion**			
Key results	18	Summarise key results with reference to study objectives	20-21
**Limitations**			
Interpretation	20	Give a cautious overall interpretation of results considering objectives, limitations, multiplicity of analyses, results from similar studies, and other relevant evidence	23-24
Generalisability	21	Discuss the generalisability (external validity) of the study results	23-24
**Other information**			
Funding	22	Give the source of funding and the role of the funders for the present study and, if applicable, for the original study on which the present article is based	27

*Give information separately for cases and controls in case-control studies and, if applicable, for exposed and unexposed groups in cohort and cross-sectional studies.

Note: An Explanation and Elaboration article discusses each checklist item and gives methodological background and published examples of transparent reporting. The STROBE checklist is best used in conjunction with this article (freely available on the Web sites of PLoS Medicine at http://www.plosmedicine.org/, Annals of Internal Medicine at http://www.annals.org/, and Epidemiology at http://www.epidem.com/). Information on the STROBE Initiative is available at www.strobe-statement.org.

For peer review only
